# Genes and pathways monotonically dysregulated during progression from normal through leukoplakia to gingivo-buccal oral cancer

**DOI:** 10.1038/s41525-021-00195-8

**Published:** 2021-05-12

**Authors:** Debodipta Das, Arindam Maitra, Chinmay K. Panda, Sandip Ghose, Bidyut Roy, Rajiv Sarin, Partha P. Majumder

**Affiliations:** 1 National Institute of Biomedical Genomics, Kalyani, India; 2 Chittaranjan National Cancer Institute, Kolkata, India; 3 Department of Oral Pathology, Dr. R Ahmed Dental College and Hospital, Kolkata, India; 4 Indian Statistical Institute, Kolkata, India; 5 Advanced Centre for Treatment Research and Education in Cancer, Mumbai, India; 6 Present Address: University of Texas Health at San Antonio, San Antonio, TX USA

**Keywords:** Cancer genomics, Cancer genomics

## Abstract

Oral squamous cell carcinoma of the gingivo-buccal region (OSCC-GB) accounts for the highest cancer morbidity and mortality among men in India. It has been observed that about one-third of individuals with oral leukoplakia, a dysplastic precancerous lesion in the oral cavity, progress to oral cancer. We aimed to identify systematic transcriptomic changes as a normal tissue in the oral cavity progresses to frank OSCC-GB. Seventy-two OSCC-GB patients, from multiple hospitals, were recruited, and transcriptome analysis of tumor and adjacent normal tissue (of all patients) and adjacent leukoplakia tissue (of a subset of 25 unselected patients with concomitant leukoplakia) was performed. We have identified many differences in the transcriptomic profiles between OSCC-GB and squamous cell carcinoma of the head and neck regions. Compared to the normal/precancerous tissue, significant enrichment of ECM−receptor interaction, PI3K-Akt signaling, cytokine−cytokine receptor interaction, focal adhesion, and cell cycle pathways were observed in OSCC-GB. Using gene set enrichment analysis, we identified a profound role of interferon receptor signaling in tumor growth by activating immune evasion mechanisms. The role of tumor-infiltrating immune cells further supported the growth and immunosuppressive mechanism of tumor tissues. Some immune evasion genes—*CD274, CD80*, and *IDO1*—were found to be activated even in the precancerous tissue. Taken together, our findings provide a clear insight into the sequential genetic dysregulation associated with progression to oral cancer. This insight provides a window to the development of predictive biomarkers and therapeutic targets for gingivo-buccal oral cancer.

## Introduction

Of all cancers, cancer of the oral cavity and lip is known to be associated with the highest morbidity and mortality among men in India^1^. Squamous cell carcinoma is the predominant histologic form in the oral cavity^2^. The most commonly affected site of oral squamous cell carcinoma, in the Indian subcontinent, is the gingivo-buccal region (OSCC-GB), which includes buccal mucosa, gingivo-buccal sulcus, lower gingiva, and retromolar trigone^3,4^. The widespread use of smokeless chewing tobacco and betel-quid containing areca nut (*areca catechu*) with or without tobacco are the main precipitating agents^4^. OSCC-GB is associated with a poor prognosis due to delayed presentation because of fear of being forced to quit tobacco on detection of disease. Despite recent advancements in cancer treatment, the overall 5-year survival rate for all stages of oral cancer is low (~60%)—which further lowers if there are regional or distant metastases^5^. The treatment of choice for OSCC-GB is surgical resection. Adjuvant postoperative radiotherapy with or without chemotherapy is offered while considering multiple risk factors including comorbidities, pathologic staging, and nodal involvement^2^.

Oral leukoplakia is the most common precancerous lesion in the oral cavity with a global prevalence of 2–3%^6^. A recent study^7^ on about 5000 individuals with leukoplakia has estimated that (a) only about one-third of individuals with leukoplakia develop oral cancer, and (b) the vast majority of individuals with leukoplakia who are found to progress to oral cancer develop the disease within 1 year of diagnosis of leukoplakia. This study^7^ has estimated that compared to the general population, individuals with leukoplakia have ~40-fold higher risk of developing oral cancer and an absolute risk of 3.3% of developing within 5 years of diagnosis of leukoplakia.

To complement the clinico-pathological findings, we have identified the landscape of genomic and epigenomic alterations in OSCC-GB patients^8,9^. Although some studies based on transcriptomic profiling of patients with head and neck cancer (HNSCC) or OSCC have been published^10–12^, these did not pertain to the GB form of OSCC that is highly prevalent in the Indian subcontinent, except for one in 12 patients^13^. Earlier, we had discovered^8^ that the landscape of genomic alterations in OSCC-GB is different from that of HNSCC or OSCC. Therefore, we sought to identify dysregulated genes in the tumor tissue based on transcriptomic analysis. Further, to identify an early biomarker, here we have studied dysregulation of gene expression in leukoplakia (precancer) tissue and in the cancer tissue of OSCC-GB patients.

## Results

### Transcriptomic alterations significantly associated with OSCC-GB tumorigenesis and comparison with head and neck cancer (TCGA)

**Fig. 1:**
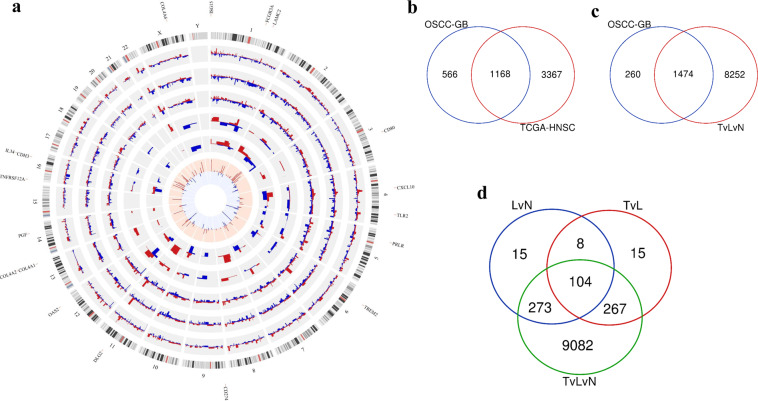
Transcriptomic landscape of development and progression of OSCC-GB. **a** Circos plot depicting differential gene expression profiles in relation to gradual malignant transformation and development of gingivo-buccal oral cancer from normal. The outermost track represents the ideogram of the human reference genome (hg19). Second and third tracks provide transcriptomic profiles of significantly differentially expressed coding genes identified and validated from the independent sets of discovery and validation cohort patients, respectively. The fourth track depicts chromosome-wise distribution of 1168 genes found significantly deregulated in the TCGA-HNSC patients in addition to OSCC-GB patients. Fifth and sixth tracks represent fold changes of 104 significantly deregulated genes, with potential involvement in malignant progression to OSCC-GB, from tumor vs. leukoplakia and leukoplakia vs. normal sample comparisons, respectively. For all six outer tracks (excluding the outermost), red and blue histograms depict fold changes in upregulated and downregulated genes, respectively. The innermost track shows red and blue bars for 175 upregulated and 93 downregulated genes from one or more KEGG pathways significantly enriched in OSCC-GB patients, respectively. The height of each bar represents the number of enriched pathway(s) in which a gene is involved. Eighteen genes, with potential involvement in oral cancer progression, from the enriched KEGG pathways, are shown outside the ideogram. Overlap of genes, significantly differentially expressed between (**b**) patients derived from gingivo-buccal oral cancer (OSCC-GB) and head and neck cancer (TCGA-HNSC). **c** OSCC-GB development and three-group (TvLvN) comparisons **d** leukoplakia vs. normal (LvN), tumor vs. leukoplakia (TvL), and three-group (TvLvN) comparisons.

Seventy-two treatment-naive OSCC-GB patients, who reported consecutively to one of the three hospitals participating in this study, were recruited into this study. The tumor purity of all the 72 tumor samples was identified in silico using the ESTIMATE algorithm^14^. The ESTIMATE scores of all, but one (which was retained), of the OSCC-GB tumor samples of this study are within the range of ESTIMATE scores of the head and neck squamous cell carcinoma (HNSC) tumor samples included in the TCGA study (Supplemental Table S1). This indicates that the tumor purity of the samples of this study is comparable to those of the TCGA-HNSC study. No evidence of HPV infection was found in any of the tumor tissues (Supplemental Table S1). Careful clinical evaluations, including histopathological assessments (see Supplemental Fig. S1 for representative microphotographs), revealed the concomitant presence of leukoplakia in 25 (35%) of the recruited patients. Transcriptomic sequencing of RNA isolated from the tumor, adjacent normal (from all patients), and adjacent leukoplakia (from 25 patients in whom present) tissue samples was performed. To enhance the robustness of the inferences, the entire cohort of 72 patients was randomly split into discovery and validation subcohorts of equal size (36 patients in each subcohort; see Supplemental Table S2 and Supplemental Fig. S2). We identified, using the cuffdiff software package^15^, 2207 protein-coding genes to be significantly (corrected *p*-value < 0.05) differentially expressed by ≥2-fold (i.e. average |log_2_ (fold-change)| ≥1) in tumor compared to paired normal samples in patients belonging to the discovery subcohort. These findings could be validated, using the paired *t*-test, in respect of 1734 of the 2207 genes using the samples from the validation subcohort. Of the 1734 genes, 804 genes (~46%) were found upregulated and the remaining 930 genes (~54%) downregulated (Fig. 1a and Supplemental Table S3). The 10 genes that were most differentially expressed in tumors compared to normal included *MMP1*, *MFAP2, MMP13, PARP12, C1QTNF6, COL4A1*, *DFNA5* (all upregulated), and *ARHGEF26*, *NFIA*, and *CTTNBP2* (all downregulated).

We have analyzed, using edgeR package (implemented in TCGAbiolinks)^16^ differential gene expression in all primary tumor samples compared to normal from TCGA-HNSC patients in whom tumor sites were mostly located in regions other than the gingivo-buccal complex. Differential gene expression profiling of the TCGA-HNSC cohort identified a total of 4535 genes significantly differentially expressed by at least 2-fold (FDR < 0.05; average |log2 (fold change)| ≥ 1) in tumor compared to normal. Of these 4535 genes, 2030 genes were upregulated and 2505 were downregulated. Of the genes that were found significantly dysregulated in OSCC-GB patients, 67% (1168 of 1734 genes) was common with the 4535 dysregulated genes of the TGCA-HNSCC patients (Fig. 1a, b, and Supplemental Table S4). Thus, one-third of the significantly dysregulated genes in OSCC-GB are unique to this form of oral cancer. Of the 1168 significantly dysregulated genes in OSCC-GB patients, ~99.5% (= 597 of 600) of all up- and ~99.7% (= 566 of 568) of all downregulated genes were found to follow similar directionality in TCGA-HNSC patients (Supplemental Fig. S3). Comparison of data on 1712 (of 1734) genes showed a significant (*p*-value < 2.2 × 10^−16^) positive correlation (*r* = 0.72) between log2-transformed average fold changes of gingivo-buccal patients included in this study (*n* = 72 paired samples) and patients (*n* = 172, unpaired samples) included in the TCGA-HNSC study (Supplemental Fig. S4).

**Fig. 2:**
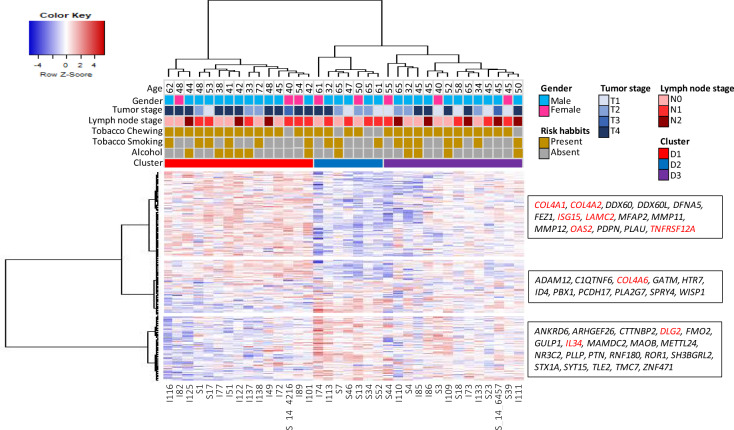
Bivariate clustering of gingivo-bucal oral cancer patients and genes based on log_2_ FPKM values of the 200 most significantly differentially expressed genes in the tumor samples of the 36 patients belonging to the validation cohort. Unsupervised hierarchical clustering of 36 patients identified three major clusters—D1 (*n* = 15), D2 (*n* = 7), and D3 (*n* = 14). The 200 genes were also clustered into three groups. (From the top) The expressions of 15 (of 85), 11 (of 52), and 21 (of 63) genes (shown inside each of the three boxes) were found to progress (increase or decrease) monotonically from normal to leukoplakia to tumor. The genes marked in red belong to one or more KEGG pathways found significantly enriched in OSCC-GB patients.

Unsupervised clustering—using Euclidean distance and Ward’s method—of 36 OSCC-GB patients (from the validation cohort), based on the 200 most significantly (with lowest corrected *p*-values among the *p*-values pertaining to the 1734 identified genes) differentially expressed genes identified three major clusters of patients—D1, D2, and D3 (Fig. 2). These clusters were associated with age [Fisher’s Exact Test *p*-value = 0.0696; old age (>60 years) vs. young age (≤ 60 years)], tumor stage [Fisher’s Exact Test *p*-value = 0.0972; higher stage (= T3 + T4) vs. lower stage (= T1 + T2)], but no lymph node involvement [Fisher’s Exact Test *p*-value = 0.999; node presence (i.e. N+) vs. absent (i.e. N0)]. The proportions of patients of T3 and T4 stages belonging to the three clusters D1, D2 and, D3 were, respectively, ~73%, ~29%, and ~43%. The proportion of patients with lymph node involvement (N+) was similar (57−64%) among the three clusters. To investigate the pattern of clustering of those TCGA-HNSC patients (*n* = 172) for whom there was malignancy in the gingivo-buccal (GB) region, we have performed unsupervised hierarchical clustering using the expression levels (FPKM values) of the same genes used to cluster the OSCC-GB patients included in our study. The tumor samples of patients in the TCGA study also corresponded to the three clusters D1, D2, and D3 (Supplemental Fig. S5); based on the TCGA data, there was no statistically significant difference in the mean periods of survival of individuals belonging to the three clusters (*p* > 0.05).

### Gene expression profiles associated with gingivo-buccal oral cancer progression

We identified 9726 genes significantly (FDR < 0.05) dysregulated in three-group comparisons, from 25 OSCC-GB patients with triad (tumor−leukoplakia−normal) data, using the R/Bioconductor-based TCC package^17^. Approximately 85% of the 1734 genes were found significantly dysregulated in the triad comparisons (Fig. 1c and Supplemental Table S5).

The 1734 genes identified significantly dysregulated in OSCC-GB patients were further tested, using paired *t*-test on 25 patients with triad data, to find potential involvement in the progression to cancer from normal oral tissue (Supplemental Fig. S2). Among these genes, the number of genes that were found to be significantly (corrected *p*-value < 0.05) dysregulated by at least 2-fold were (a) 478 in leukoplakia compared to normal, and (b) 548 in tumor compared to leukoplakia. We identified 112 genes to be significantly (corrected *p*-value < 0.05) monotonically dysregulated by ≥2-fold both in (a) leukoplakia compared to normal, and in (b) tumor compared to leukoplakia. Of the 112 genes, 104 were validated to be significantly dysregulated in the three-group (tumor− leukoplakia−normal) comparison (Fig. 1d). Fifty-eight (~56%) and forty-six (~44%) genes were found to be up- or downregulated, respectively, by >2-fold, during the progression from normal to leukoplakia to cancer (Supplemental Table S6).

### Pathways enriched for development and progression of OSCC-GB

**Fig. 3:**
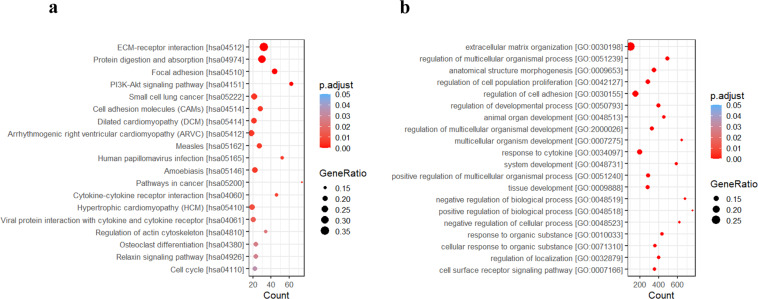
Visualization of results of functional enrichment analysis of 1734 significantly differentially expressed genes in OSCC-GB patients. **a** The list of KEGG pathways (*n* = 19) found significantly enriched. **b** Twenty most significantly enriched GO BP (biological process) terms. Both the lists are arranged from the top in ascending order of the *p*-value adjusted for multiple testing.

Pathway enrichment analysis was done separately using the genes identified to be significantly dysregulated during (a) OSCC-GB development (1734 genes), and (b) during progression from normal through leukoplakia to cancer (104 genes). A total of 19 KEGG pathways and 813 GO BP (biological process) terms were found significantly enriched in OSCC-GB patients during the development of tumor. Extracellular matrix organization (GO0030198)−receptor interaction (hsa04512) was found as the most significantly enriched GO BP term and KEGG pathway among all pathways (Fig. 3). This pathway was also found to be significantly enriched when the analysis was done using only the 104 genes that showed significant and monotonic direction of dysregulation from normal through leukoplakia to cancer tissue (Supplemental Fig. S6). Other significant pathways/interactions included the PI3K-Akt signaling pathway (hsa04151), cytokine−cytokine receptor interaction (hsa04060), viral protein interaction with cytokine, and cytokine receptor (hsa04061), cell cycle (hsa04110), relaxin signaling pathway (hsa04926), and human papillomavirus infection (hsa05165). Apart from the ECM−receptor interaction, pathways related to cell adhesion, viz. focal adhesion (hsa04510), cell adhesion molecules (hsa04514), and regulation of actin cytoskeleton (hsa04810), were also found to be enriched. The response to cytokine (GO:0034097), regulation of cell population proliferation (GO:0042127), and regulation of cell adhesion (GO:0030155) were among the 20 most significantly enriched GO BP terms.

Among the 104 genes identified and validated to be associated with progression to cancer, 18 genes also belong to one or more pathways significantly enriched for OSCC-GB development (Fig. 1a). Of these 18 genes, 15 were monotonically upregulated while three were monotonically downregulated from normal to leukoplakia to cancer tissues. The upregulated genes—*COL4A1, COL4A2, COL4A6, LAMC2, PGF, TLR2, CXCL10, and TNFRSF12A*—were found linked with ECM−receptor interaction (first four genes among those listed above), PI3K-Akt signaling pathway (first six genes), and cytokine−cytokine receptor interaction (last two genes) pathways. Most of the remaining upregulated genes—*CD274, CD80, ISG15, FCGR3A, TREM2*, and *OAS2*—are immune-related. Similarly, among the three monotonically downregulated genes, *PRLR* and *IL34* belong to the cytokine−cytokine receptor interaction pathway; *PRLR* also belongs to the PI3K-Akt signaling pathway (Supplemental Figs. S7–S9).

### Enriched gene sets in gingivo-buccal tumor samples

**Fig. 4:**
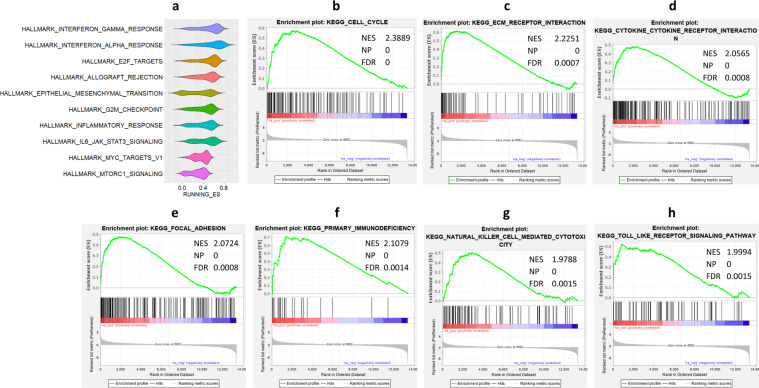
Significant enrichment of Hallmark gene sets and KEGG pathways in OSCC-GB tumor samples using GSEA. **a** Violin plot showing the distribution of enrichment scores (ES) of all ranked genes that belong to the 10 most significantly enriched Hallmark gene sets. **b**–**h** Enrichment plots of 7 of the 10 most significantly enriched KEGG pathways. These include **b** cell cycle, **c** ECM receptor interaction, **d** cytokine−cytokine receptor interaction, **e** focal adhesion, **f** primary immunodeficiency, **g** natural killer cell-mediated cytotoxicity, and **h** Toll-like receptor signaling pathway.

We have performed gene set enrichment analysis (GSEA) using genes ranked based on average log2 (fold change) values in tumor compared to paired normal samples from all 72 patients. Thirty-five Hallmark and 57 KEGG pathways were found to be significantly enriched by GSEA analysis. The ten most significantly enriched Hallmark gene sets in tumor samples from OSCC-GB patients are depicted in Fig. 4a. Of all significantly enriched KEGG pathways, the 10 most significantly enriched pathways include cell cycle, ECM receptor interaction, cytokine−cytokine receptor interaction, focal adhesion, primary immunodeficiency, natural killer cell-mediated cytotoxicity, Toll-like receptor signaling pathway; all of these pathways are found enriched in tumor tissues compared to normal (Fig. 4b–h). Of these 10 pathways, the first four were also identified to be significantly enriched KEGG pathways based on pathway enrichment analysis using significantly differentially expressed genes in OSCC-GB patients (Fig. 3a).

### Landscape of infiltrating immune cells in OSCC-GB patients

Profiles of infiltrating immune cells were identified for the development (from all 72 patients) and progression (from 25 patients with concomitant presence of leukoplakia) of OSCC-GB using CIBERSORT (http://cibersort.stanford.edu/). Paired-sample *t*-test, based on log2 transformed proportions, was used to identify significant differences in infiltrating immune cells between tumor and adjacent normal samples, from 72 patients. Significantly different (higher or lower) proportions of eight (of a total of 22) infiltrating immune cell types were identified in tumor compared to paired normal tissues (Supplemental Fig. S10). The proportions of T cells CD4 memory activated, NK cells activated, macrophages M1, and mast cells activated were found to be >2-fold higher, whereas those of plasma cells, monocytes, and neutrophils were identified to be >2-fold lower in the microenvironment of tumor compared to that of paired normal tissues. The proportions of B naive cells were significantly (*p* < 0.05) downregulated by <2-fold in the microenvironment of tumor vs. normal tissues (Supplemental Table S7 and Supplemental Fig. S10). For leukoplakia vs. normal and tumor vs. leukoplakia comparisons, we have considered only eight immune cell types (Supplemental Table S7) with significant infiltration in tumor compared to normal tissue microenvironments. Significantly (*p* = 0.008) lower infiltration of the plasma cells was observed in leukoplakia compared with paired normal tissue microenvironments in 25 patients (Supplemental Fig. S11). Differences in the degree of infiltration of both macrophages M1 (*p* = 0.011) and activated mast cells (*p* = 0.012) were significantly higher in tumor compared to leukoplakia microenvironments (Supplemental Fig. S11 and Supplemental Table S7).

## Discussion

In OSCC-GB, we have identified and validated 1734 coding genes that are significantly (≥2 fold) dysregulated in tumor tissue compared to adjacent normal. Nearly 70% of these genes were also detected as significantly differentially expressed in head and neck squamous cell carcinoma (TCGA-HNSC) patients. The correlation of fold changes for the significantly differentially expressed genes was also positive (*r* = 0.72) and significant (*p* = 2.2 × 10^−16^) between Indian, non-Caucasian patients (our study), and Caucasian patients (TCGA-HNSC study). By analyzing the tissue from precancerous lesions (leukoplakia) in a set of individuals, we have shown here that ~6% of the significantly dysregulated genes (104 of 1734) were also significantly associated with progression from leukoplakia to cancer.

The ten most significantly differentially expressed genes in OSCC-GB tumor tissue included seven upregulated—*MMP1*, *MFAP2, MMP13, PARP12, C1QTNF6, COL4A1*, *DFNA5*—and three downregulated genes—*ARHGEF26*, *NFIA*, and *CTTNBP2*. Earlier studies had found similar features in HNSCC and/or OSCC. These include upregulation of *MMP1* and *MMP13*^18,19^, and, *MFAP2, COL4A1,* and *DFNA5*^20–22^. We had earlier^8^ identified significant copy number amplification of *MMP1* and *MMP13* in OSCC-GB patients. Some of the other upregulated genes have been found to be similarly associated with features of other cancers. For example, overexpression of *C1QTNF6* was found in gastric carcinoma^23^ (although it has been reported that the *C1QTNF6* protein significantly suppresses the proliferation of oral squamous cell carcinoma cells)^24^; *PARP12* upregulation was associated with the inhibition of metastasis in hepatocellular carcinoma cell lines^25^.

Of the downregulated genes in OSCC-GB, a significant decrease in the expression of *NFIA* was noted in tongue squamous cell carcinoma that was associated with poor overall survival in head and neck cancer^26^. Contrary to our findings in oral cancer, upregulation of *ARHGEF26* (also known as *SGEF*) and *CTTNBP2* was observed in glioblastoma^27^.

The overall picture that emerges is that there are significant changes in the expression of genes involved in tissue remodeling, the interaction of tumor cells with vascular and endothelial cells, cell death, and migration.

We and others have found the ECM−receptor pathway to be significantly enriched in OSCC-GB patients^13^. A noteworthy finding of this study is that significant alterations in expressions of genes in this pathway are seen early in the transition of normal tissue to oral cancer. If validated in an independent study with a larger number of samples, expressions of some key genes (e.g., *COL4A1, COL4A2, COL4A6, LAMC2*) on this pathway may be used as early biomarkers of OSCC-GB. We have, based on mutational profiling, earlier identified alterations of genes in these, and other pathways such as the PI3K-Akt signaling pathway, to significantly enhance the risk of OSCC-GB^8^. The PI3K-Akt signaling pathway was also found to be activated in oral epithelial dysplasia and early tongue cancer^28^. Not surprisingly, therefore, PI3K inhibitors were shown to be effective in the treatment of OSCC patients^29^. Relaxin is known to stimulate multiple signaling pathways including the PI3K^30^. Thus, our observation of enrichment of the relaxin signaling pathway may be indicative of an additional link with the PI3K/Akt pathway in OSCC-GB patients. Another significant observation in our study, also noted earlier^31^, was the enrichment of genes in the cytokine−cytokine receptor interaction pathway (KEGG) and response to cytokine (GO BP) in OSCC-GB tumor tissues compared to normal tissues (Figs. 3 and 4). As salivary biomarkers, cytokines have been associated with early diagnosis^32^ and progression of oral cancer^33^. Products of two key genes—*CXCL10* and *IL34*—on this pathway are known to help in the stimulation of monocytes, natural killer, and T-cell migration, and in promoting the differentiation and viability of monocytes and macrophages. As in many other cancers, the cell cycle-related pathway was found dysregulated (Fig. 4b) in oral cancer^34^. However, even though human papillomavirus (HPV) infection was commonly found in TCGA-HNSC patients^10^, HPV was absent in most OSCC-GB tumor tissues. No significant enrichment of HPV infection-associated genes was identified by GSEA analysis.

Of the 18 genes that we found consistently and significantly associated with progression from normal to precancer state (leukoplakia) to frank cancer state, many are type IV collagen genes and associated with the ECM−receptor interaction pathway. Upregulation of collagen type IV alpha chain (1, 2, 6) family of genes and the *LAMC2* gene that we found on the enriched ECM−receptor pathway have been noted earlier^35,36^. The Toll-like receptor gene, *TLR2*, found associated with the PI3K-Akt signaling pathway, is known to be expressed on the keratinocytes of dysplastic epithelium and OSCC^37^.

GSEA revealed significant enrichment of hallmark immune—interferon gamma (IFN-γ), interferon alpha (IFN-α), and inflammatory—response pathways and the hallmark IL6/JAK/STAT3 signaling pathway in OSCC-GB (Fig. 4a). We have observed significant upregulation of *IFNG*, *JAK3*, *STAT1*, and *STAT2* in tumor compared to normal samples (Supplemental Table S3). Type II interferons (IFN-γ) released from activated T-cells induce activation of JAK, which plays a role in signal-transduction and activation of the STAT signaling pathway. This results in the activation of *PDL1* transcription in tumor cells, which in turn negatively regulates the anti-tumor T cell response by binding with the PD-1 receptor on the surface of T cells. Binding of PD-1 with its ligands PD-L1 and PD-L2 expressed on tumor cell surfaces upon exposure to interferons activates tumor immune evasion^38^. We have observed significant upregulation of *CD274* (also known as *PDL1*) and *PDCD1LG2* (also known as *PDL2*) in OSCC-GB patients. Type I interferons (including IFN-α) can also trigger phosphorylation and subsequent activation of STAT3^39^. High levels of certain inflammatory cytokines like IL-6 in the tumor microenvironment can bind to membrane-bound receptor molecules (IL-6R) in tumor cells. This complex subsequently leads to activation of the JAK/STAT3 signaling pathway^40^. Therefore, IFN-γ supports the promotion of tumorigenesis by immune evasion, in OSCC-GB patients, through activation of *PDL1* transcription in tumor cells via the IL6/JAK/STAT3 signaling pathway.

We have also observed significant enrichment in the Hallmark gene sets—E2F targets, G2M checkpoint, and MYC targets v1—associated with cell proliferation^41^. Epithelial to mesenchymal transition enrichment, observed in OSCC-GB, is an indicator of tumor cells losing their cell–cell junctions and adherence to the basement membrane^42^. Activation of mTOR complex 1 (MTORC1) signaling helps in protecting cancer cells from autophagy-induced apoptosis. PI3K signaling was also found indirectly activating MTORC1, primarily through AKT. We have observed enrichment of MTORC1 signaling (Fig. 4a) and the KEGG PI3K-Akt signaling pathway (Fig. 3a) in OSCC-GB patients. Interestingly, clinical trials are ongoing to target mTOR in HNSCC or premalignant lesions of the oral cavity^43^. These observations indicate the probable role of activated MTORC1 signaling in protecting gingivo-buccal oral cancer cells from autophagy-induced apoptosis.

Earlier studies have shown that cancer cells, during their progression, can modulate the normal developmental processes to create an immunosuppressive tumor microenvironment (comprising immune cells)^44^. Tumor-associated macrophages orchestrate angiogenesis and thus promote tumor initiation and growth. They also inhibit the function of tumor-infiltrating CD8 T cells through PDL1/PD1 interaction. Tumor-associated macrophages stimulate the generation of induced Tregs (regulatory T cells) from CD4+ T cells^44^. Mutant p53-induced JAK-STAT signaling has been shown to increase macrophage and CD4+ T cell frequencies in the tumor microenvironment, and reduce CD8+ T cell population^45^. Thus, significant enrichment of macrophages M1 activated CD4 memory T cells in the tumor microenvironment and that of the hallmark IL6/JAK/STAT3 signaling pathway in tumor tissues indicate initiation, growth, and escape of OSCC-GB tumor cells from host immune response.

Among genes potentially responsible for OSCC-GB progression, upregulation of immune checkpoint genes—*CD274, CD80*, and *IDO1*—was also observed in head and neck cancer patients^46^. We have already found significant upregulation of *CD274* and *CD80* in OSCC-GB patients to be associated with significant hypomethylation in the promoter region of these genes^9^. Similar to our observation, a recent study has found these genes to be involved in early immune evasion in the pre-invasive stages of lung squamous carcinogenesis^47^. The biomarkers for immunomodulatory treatments in head and neck cancer patients are now emerging^48^. Expression based on mRNA and immunohistochemical studies has identified ISG15 to upregulate consistently during oral tumorigenesis^49^. Of the genes coding matrix metallopeptidases, differential expression of *MMP11* was found as an early event in oral tumor progression^50^, but that of *MMP12* could not be associated with oral cancer progression from previous studies. An immunohistochemical analysis of KRT13 had shown a loss of expression in OSCC cells while it was expressed in normal differentiated basal and suprabasal oral mucosa epithelial cells^51^. Thus, *KRT13* can also be treated as a biomarker for progression to OSCC-GB.

In summary, based on profiling of the transcriptomic landscape of gingivo-buccal oral cancer and precancer, we have identified genes that putatively enhance the risk of transition from precancer to frank oral cancer. While there were many similarities, some differences were also found in the transcriptomic profiles between OSCC-GB and HNSCC tumors. Pathways such as ECM−receptor interaction, cytokine−cytokine receptor interaction, focal adhesion, cell cycle, and PI3K-Akt signaling correlate with dysregulation of the underlying molecular mechanisms in OSCC-GB. Evidence of involvement of immune escape by tumors using interferon receptor signaling mechanism was found. Infiltrating immune cells in the tumor microenvironment support growth and immunosuppressive mechanism of tumor tissues. The progressive upregulation of immune checkpoint genes *CD274, CD80*, and *IDO1* is potentially helpful to understand immune evasion mechanisms during the progression of cells to malignancy. These results after appropriate validation may help identify predictive biomarkers and novel therapeutic targets for gingivo-buccal oral cancer.

## Methods

### Patient selection

This study was approved by the Research Ethics Committees of Advanced Centre for Treatment, Research and Education in Cancer (ACTREC), Dr. R. Ahmed Dental College & Hospital (RADCH), Chittaranjan National Cancer Institute (CNCI), National Institute of Biomedical Genomics (NIBMG), and Indian Statistical Institute (ISI). A signed informed consent was obtained from each participant before their enrolment into this study.

Seventy-two patients suffering from OSCC-GB who reported for treatment to ACTREC, RADCH, or CNCI were recruited into this study. Clinical examination revealed the presence of leukoplakia in the oral cavities of 25 of these patients; leukoplakia tissue was collected from each of these 25 patients. Tumor and adjacent normal tissue samples were collected from all 72 patients. To reduce the chance of false-positive results due to normal tissue contamination, we have considered only those tumor samples with at least 80% tumor cells based on biopsy reports. Leukoplakia and normal tissues were subsequently confirmed by histology. Tissue samples were preserved in RNAlater immediately after collection. Patient details are provided in Table 1 and Supplemental Table S2.

**Table 1:** Demographic and clinical characteristics of cohorts of study participants with OSCC-GB and premalignant lesion (leukoplakia).

Characteristics	OSCC-GB patients		OSCC-GB patients from whom leukoplakia tissues were also collected
	Association discovery set	Association validation set	
	(*n* = 36)	(*n* = 36)	(*n* = 25)
Age (in years)			
Range	26−70	32−72	32−70
Mean	50.33 ± 11.19	49.08 ± 10.43	51.84 ± 10.23
<40	7	5	3
40−45	6	12	5
46−50	4	6	4
51−55	7	4	5
56−60	5	1	2
>60	7	8	6
Gender			
Male	31	28	20
Female	5	8	5
Risk-habit			
Tobacco chewing	17	18	11
Tobacco chewing and smoking + alcohol	15	13	9
Tobacco Smoking + alcohol	3	4	4
None	1	1	1
Tumor stage^**a**^			
T1	10	8	18
T2	11	10	7
T3	1	1	0
T4	14	17	0
Lymph node invasion^**a**^			
N0	17	14	9
N+	19	22	16
Leukoplakia histopathology			
Mild dysplasia	-	-	13
Moderate dysplasia	-	-	10
Severe dysplasia	-	-	2

^a^All patients were M0, i.e., no metastasis at first presentation when tissue samples were collected for analysis.

### RNA extraction and library preparation

Total RNA from all tissue samples was extracted using AllPrep DNA/RNA Mini Kit (QIAGEN). The concentration and quality of isolated total RNA were checked using a NanoDrop 2000 spectrophotometer and Agilent 2100 Bioanalyzer using the RNA 6000 Nano Kit (Thermo Fisher Scientific). For all samples, the OD260/OD280 ratio was ≥2 and RNA Integrity Number (RIN) was ≥7.0. Sequencing cDNA libraries were prepared from these RNA samples after rRNA depletion using the TrueSeq RNA Sample Preparation Kit (Illumina). The quality and quantity of the cDNA libraries were assessed in Agilent 2100 Bioanalyzer using a High Sensitivity DNA chip as well as in 7900HT Fast Real-Time PCR System (Life Technologies) using a Kapa Library Quantification Kit (Kapa Biosystems). The sequencing libraries were pooled (three samples per pool) and 2 × 100 bp paired end sequencing was performed on HiSeq-2000 and HiSeq-2500 (Illumina). Assays were performed according to the relevant manufacturer’s protocol.

### Data processing

The quality of the FASTQ files, obtained by demultiplexing the raw sequencing data, was verified using FastQC (v. 0.10.1) software (www.bioinformatics.babraham.ac.uk/projects/fastqc/). More than 50 million paired-end good-quality RNA sequence reads were generated from each (tumor/leukoplakia/normal) sample. The reads were then aligned to the human reference transcript [GTF file downloaded from the Illumina iGenome (ftp://ftp.illumina.com/Homo_sapiens/UCSC/hg19/)] and reference genome (hg19 with decoy sequence) using the Tuxedo suite [TopHat2 (v. 2.1.1)^52^, Bowtie 2 (v. 2.3.4.1)^53^] and SAMtools (v. 0.1.19)^54^, with default parameters. To minimize false-positive inferences, (a) all multi-mapped and non-concordant reads were discarded using SAMtools, and (b) duplicate reads were identified using PICARD (https://github.com/broadinstitute/picard) MarkDuplicates (v 2.17.0) command and were eliminated from further analyses. Mapped transcripts were assembled using Cufflinks (v. 2.2.1)^15^ with default settings.

### Differential gene expression in OSCC-GB patients

The cohort of 72 patients was randomly split into two equal subcohorts, for the discovery of dysregulated expression of genes and validation. Differential gene expression was identified from tumor−normal paired samples of 36 patients (discovery subcohort) using cuffdiff with default parameters. Genes discovered to be significantly differentially expressed in patients were considered for validation. Paired-sample *t*-test, based on FPKM values normalized over 36 tumor−normal paired samples from the validation cohort patients using cuffnorm, was performed to compare mean expression levels in tumor and normal samples. The Benjamini−Hochberg method was used to correct *p*-values for multiple testing.

### Curation and analyses of TCGA RNA-Seq data

The TCGAbiolinks on R/Bioconductor^16^ was used to download the gene expression quantification and clinical data from the TCGA harmonized database. Genes significantly differentially expressed in 500 primary tumor tissues compared with 44 normal tissues from head and neck squamous cell carcinoma patients (TCGA-HNSC) were identified using the function TCGAanalyze_DEA from TCGAbiolinks GUI with default settings. The edgeR package (with TCGAanalyze_DEA) was used to identify gene expression differences, in tumor vs. normal tissues, using the likelihood ratio test. To reduce the chance of false-positive inferences, a threshold value of 0.25 was selected as a mean for filtering quantile normalized HTSeq count data.

We have compared our data (*n* = 72) on gingivo-buccal tumors with the subset of the TCGA-HNSC data (*n* = 172) that pertained to tumors from the same region of the oral cavity of each patient. However, one notable difference between the data sets was that while we had normal tissue of the oral cavity from every patient, the TCGA data only had 17 normal samples. We calculated the Pearson correlation coefficient of the average log_2_ fold change of expression between tumor and normal tissues for each gene between our data and the TCGA-HNSC data.

### Gene expression progression from normal to cancer

Expression levels of those genes identified and validated as significantly differentially expressed in OSCC-GB tumor tissue compared to normal were then further explored in leukoplakia tissue to discover whether there was monotonicity in the direction of change of expression level from normal through precancer to cancer. This analysis was done using normalized expression (FPKM) values of these genes in those 25 patients from whom tumor−leukoplakia−normal triad samples were available. We have used a paired-sample *t*-test with Benjamini−Hochberg multiple-testing correction for comparing transcriptomic profiles between leukoplakia vs. normal and tumor vs. leukoplakia samples. We considered a gene to be involved in the progression from normal through precancer (leukoplakia) to cancer if the level of expression of the gene was (a) significant (corrected *p*-value <0.05) with at least 2-fold change between normal and leukoplakia, with the same direction of change (irrespective of statistical significance or fold change) from leukoplakia to cancer, or (b) significant (corrected *p*-value < 0.05) with at least 2-fold change between leukoplakia and cancer, with the same direction of change (irrespective of statistical significance or fold change) from normal to leukoplakia. Details of the methodology used are depicted in Supplemental Fig. S2.

We have further performed three-group (tumor−leukoplakia−normal) comparisons, from 25 patients with triad data, using the R/Bioconductor-based TCC package (v. 1.28.0)^17^. The raw read counts from the BAM file of each triad samples were generated using HTSeq-count (v. 0.12.4)^55^. To identify differentially expressed genes from the read counts, we have applied the SSS-S pipeline of the TCC package with an iteration of 3, as per recommendations. Genes with FDR < 0.05, and found expressed (read count ≥1) in all 75 samples, were considered as significantly differentially expressed in three-group comparisons.

### Pathway enrichment analysis

Genes identified and validated to be significantly dysregulated in OSCC-GB patients were used for functional enrichment analysis with the ClueGO plugin of Cytoscape (v. 3.7.2)^56^. Pathway information was used from the Gene Ontology (GO) terms and Kyoto Encyclopedia of Genes and Genomes (KEGG) pathways. The enrichment analysis was performed using a right-sided hypergeometric test, with the Benjamini−Hochberg multiple-testing corrected *p*-value < 0.05. A similar analysis was performed for the gene sets potentially involved in the gingivo-buccal oral cancer progression from normal to cancer.

### Gene set enrichment analysis

Gene expression data from all the 72 patients were analyzed, using GSEA software (v. 4.0.3)^57^, to identify gene sets enriched in OSCC-GB tumor samples. Genes, expressed (normalized FPKM > 0) in all the 72 tumor−normal paired samples, were ranked based on average log2 (fold change) values in tumor samples compared to normal samples. The ranked genes were then analyzed for gene set enrichment using Hallmark & KEGG gene set collections from the Molecular Signatures Database (MSigDB) v 7.1^41^. GSEA pre-ranked was performed using default settings including the number of permutations set at 1000. Significantly enriched gene sets identified met the following criteria: |normalized enrichment scores (NES) | >1, nominal *P*-value (NP) < 0.05, and false discovery rate (FDR) < 0.25.

### Assessment of tumor-infiltrating immune cells

Normalized gene expression (FPKM) values from 72 tumor and adjacent normal samples were analyzed using CIBERSORT^58^ (http://cibersort.stanford.edu/) to assess the relative proportions of 22 types of infiltrating immune cells in OSCC-GB patients. To identify infiltration of immune cells in precancerous lesions, the FPKM values from 25 tumor, leukoplakia, and adjacent normal samples were also analyzed separately. The CIBERSORT algorithm was run with default settings using the LM22 signature at 100 permutations. Significant differences in infiltrating immune cells between tumor and adjacent normal samples, from 72 patients, were identified using paired-sample *t*-test using log2 transformed proportions. The same statistical method was applied to leukoplakia vs. normal, and tumor vs. leukoplakia comparisons of immune cells.

### Reporting Summary

Further information on research design is available in the Nature Research Reporting Summary linked to this article.

## Supplementary information

Supplementary Information

Reporting Summary

## Data Availability

The aligned BAM files for RNA-Seq data from the 72 tumor−normal paired samples are available from EGA under study ID EGAS00001003893. The BAM files for RNA-Seq data from the 25 oral leukoplakia samples are available from ENA under study accession PRJEB42982.
