# Supplementary material for: Genes and pathways monotonically dysregulated during progression from normal through leukoplakia to gingivo-buccal oral cancer
Source: NPJ Genom Med. 2021 May 12;6:32. doi: 10.1038/s41525-021-00195-8 (PMC8115176; doi:10.1038/s41525-021-00195-8)
Supplement: Supplementary file 1 — Supplementary Information [file 41525_2021_195_MOESM1_ESM.pdf]

Supplemental Information

Contents

Supplemental Figure S1 2
Supplemental Figure S2 3
Supplemental Figure S3 4
Supplemental Figure S4 5
Supplemental Figure S5 6
Supplemental Figure S6 7
Supplemental Figure S7 8
Supplemental Figure S8 9
Supplemental Figure S9 10
Supplemental Figure S10 11
Supplemental Figure S11 12
Supplemental Table S1 13
Supplemental Table S2 17
Supplemental Table S3 19
Supplemental Table S4 61
Supplemental Table S5 89
Supplemental Table S6 125
Supplemental Table S7 128

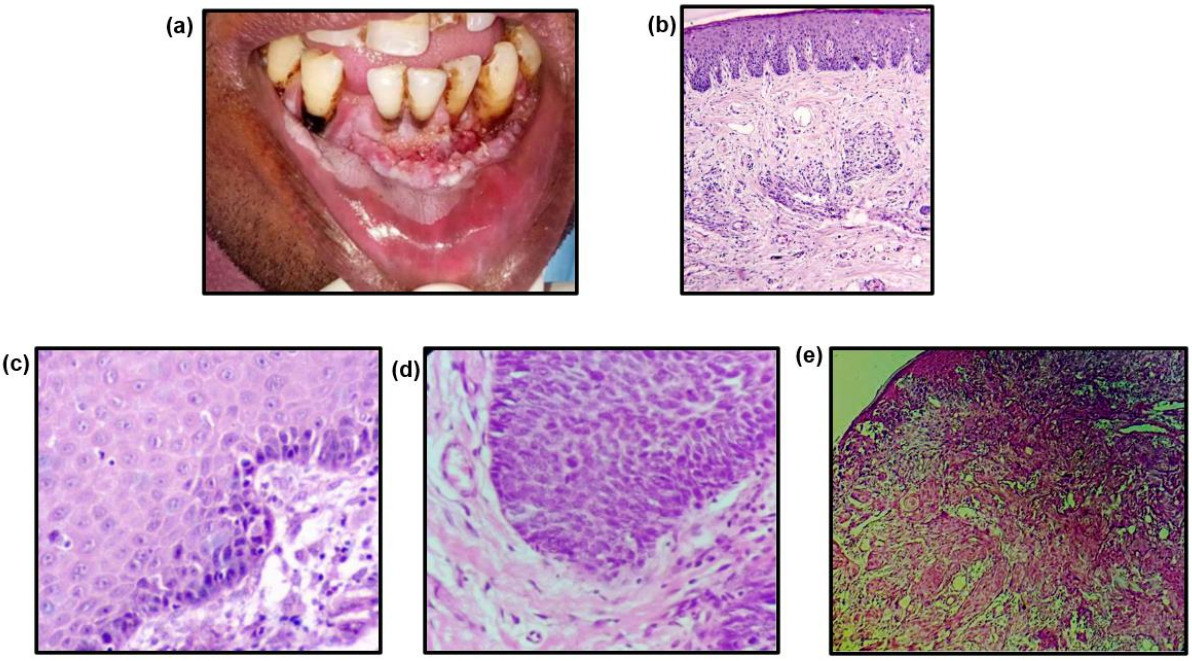

Supplemental Figure S1:

(a) Clinical photograph showing oral malignant lesion with adjacent leukoplakia.

Microphotographs of:

(b) Histology of normal oral mucosa (10x magnification)

(c)-(d) Histopathology of leukoplakia showing different grades of dysplasia (40x m magnification)

(e) Microphotograph of histopathology of oral squamous Cell carcinoma showing invading neoplastic epithelial islands (10x magnification; image digitally sharpened to show islands more clearly)

Photo of the patient who participated in this study has been included here with the explicit written consent of the patient that his photo can be published in a scientific journal describing the results of the study.

(a) CANCER DEVELOPMENT

Discovery cohort

36 patients
72 samples

Tumor vs. Normal (TvN)	
Tumor samples (n=36)	*Normal samples (n=36)*
# Genes tested = G1<br># Genes significantly differentially expressed by ≥ 2 fold = G2	

Validation cohort

36 patients
72 samples

Tumor vs. Normal (TvN)	
Tumor samples (n=36)	*Normal samples (n=36)*
# Genes tested = G2<br># Genes significantly differentially expressed by ≥ 2 fold = G3	

(b) CANCER PROGRESSION

(i) Pair-wise comparison

25 patients
75 samples

Leukoplakia vs. Normal (LvN)		Tumor vs. Leukoplakia (TvL)	
Leukoplakia samples (n=25)	*Normal samples (n=25)*	*Tumor samples (n=25)*	*Leukoplakia samples (n=25)*
# Genes tested = G3<br># Genes significantly differentially expressed by ≥ 2 fold = L1<br># Genes (of LN) with unidirectional fold change from normal to leukoplakia to tumor = L2		# Genes tested = G3<br># Genes significantly differentially expressed by ≥ 2 fold = T1<br># Genes (of TL) with unidirectional fold change from normal to leukoplakia to tumor = T2	
# Genes common between L2 & T2 = P1			

(ii) Multi-group comparisons

25 patients
75 samples

Tumor vs. Leukoplakia vs. Normal (TvLvN)		
Tumor samples (n=25)	*Leukoplakia samples (n=25)*	*Normal samples (n=25)*
# Genes tested = G1<br># Genes significantly differentially expressed = G4<br># Genes common between P1 & G4 = P2		

Supplemental Figure S2: Flow diagram depicting the methods of identification of genes associated with (a) development, and (b) progression of gingivo-buccal oral cancer from normal tissue by (i) pair-wise comparison using t-test on selected genes, and (ii) multi-group comparisons using TCC package on all genes.

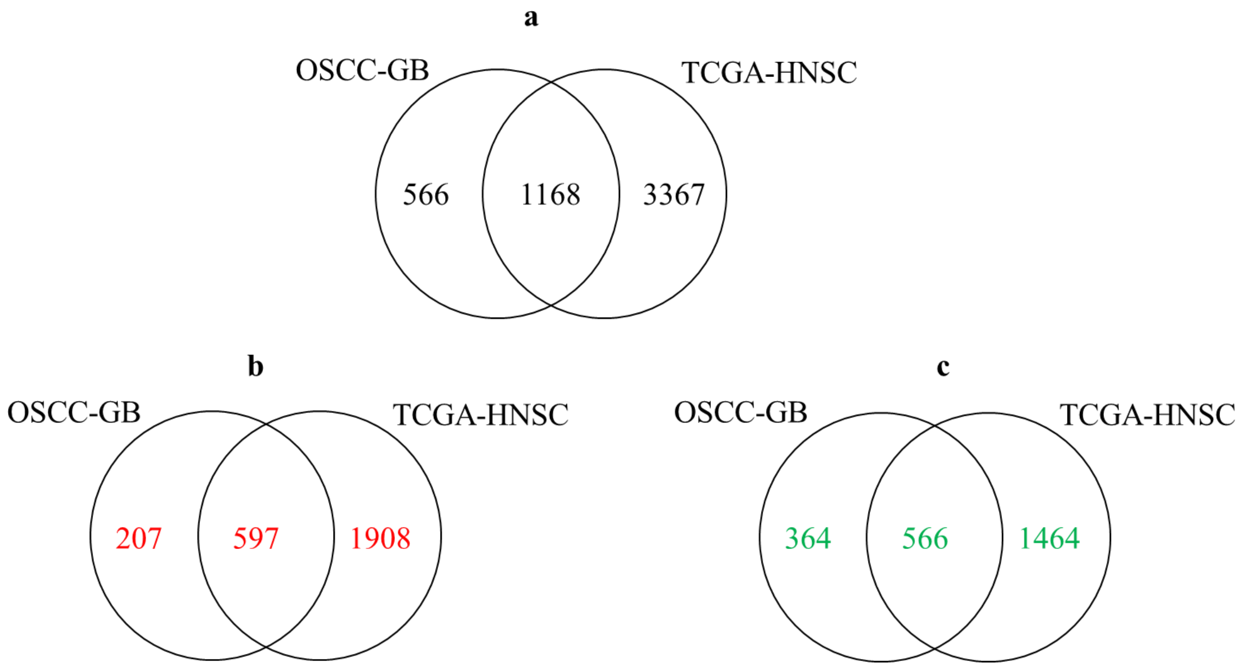

Supplemental Figure S3: Venn diagram representation to visualize overlap of genes, found significantly differentially expressed (a), or upregulated (b), or downregulated (c), between patients derived from gingivo-buccal oral cancer (OSCC-GB), and head & neck cancer (TCGA-HNSC).

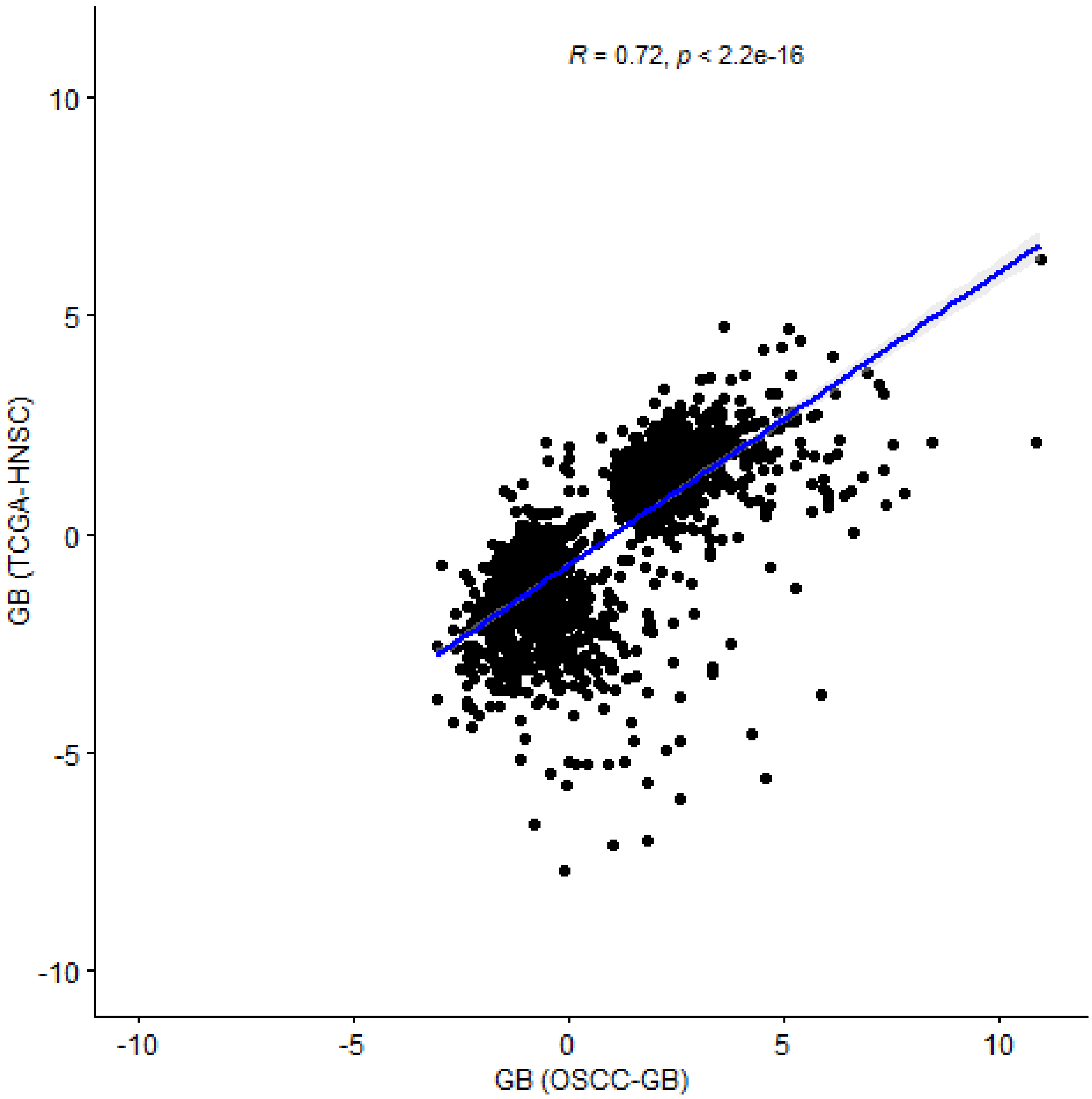

Supplemental Figure S4: Scatter diagram showing the relationship of log2 transformed average fold-change values of 1712 (of 1734) genes from all gingivo-buccal oral cancer patients in the present study with that in TCGA-HNSC study, irrespective of whether these genes were also found significantly differentially expressed in all TCGA-HNSC patients. Each point on the scatter diagram indicates log2 transformed fold-change value of a gene, averaged over 72 tumor-normal paired samples from OSCC-GB patients (this study), obtained by taking the ratio of average FPKM values from 172 tumor and 17 normal samples from the TCGA-HNSC patients with affected gingivo-buccal (GB) regions. Three outlier genes - *FAM72A*, *GPR143*, and *ZNF880* – were removed from this comparison due to very high average fold-change values obtained from our present study. The blue line indicates the regression line. Coefficient & p-value from the Pearson's correlation between the two data sets has been denoted as R and p, respectively.

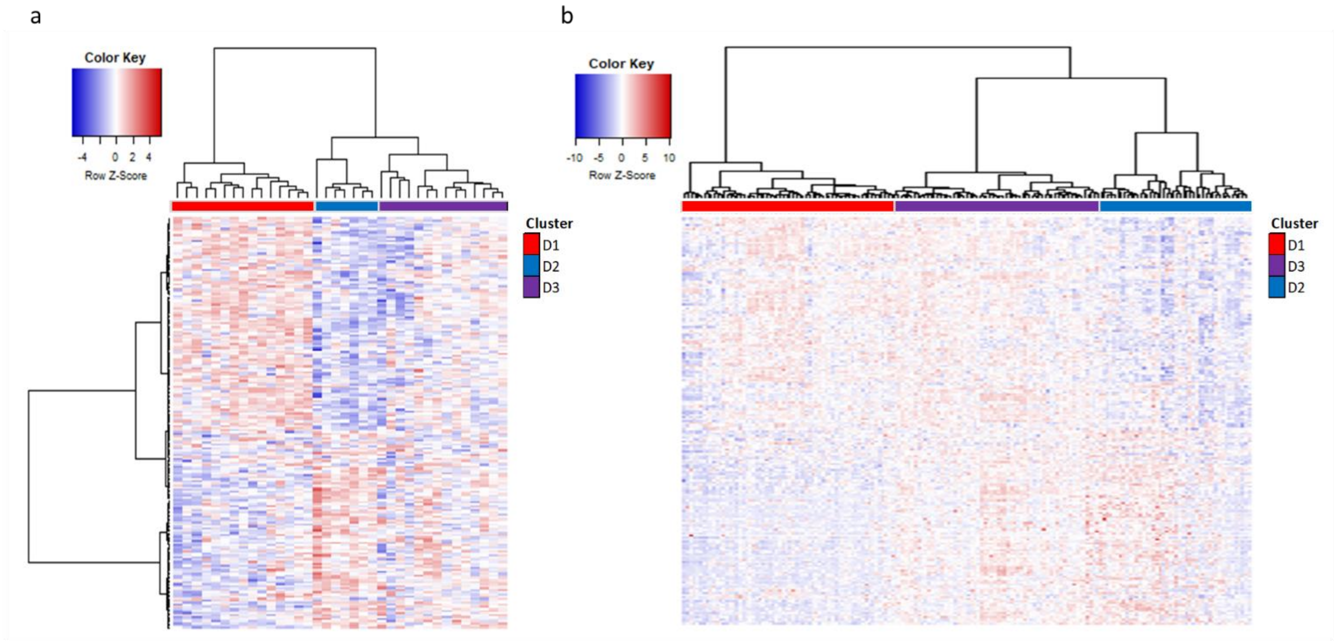

Supplemental Figure S5: Unsupervised hierarchical clustering and heatmap using (a) log2 FPKM values of the 200 differentially expressed genes in the tumor samples of GB patients from our OSCC-GB study (n=36; validation cohort), and (b) using the log2 (FPKM+0.1) values of the same genes, except one (SCO2 for which data were unavailable for the TCGA-HNSC samples), in the tumor samples of GB patients included in the TCGA-HNSC study (n=172). The clustering patterns of tumor samples of OSCC-GB patients included in the present study and those of the TCGA study are similar.

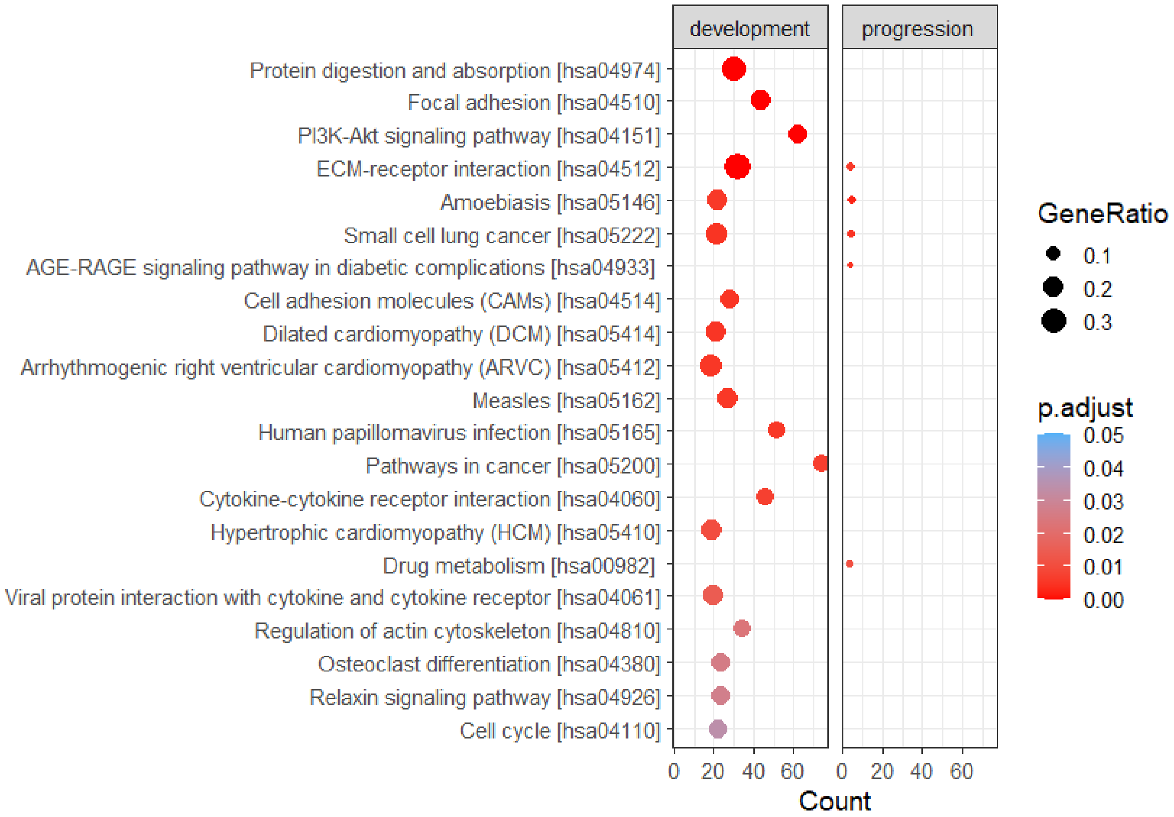

Supplemental Figure S6: KEGG pathways found significantly enriched with the 1734 and 104 genes identified to be responsible for cancer development and progression, respectively.

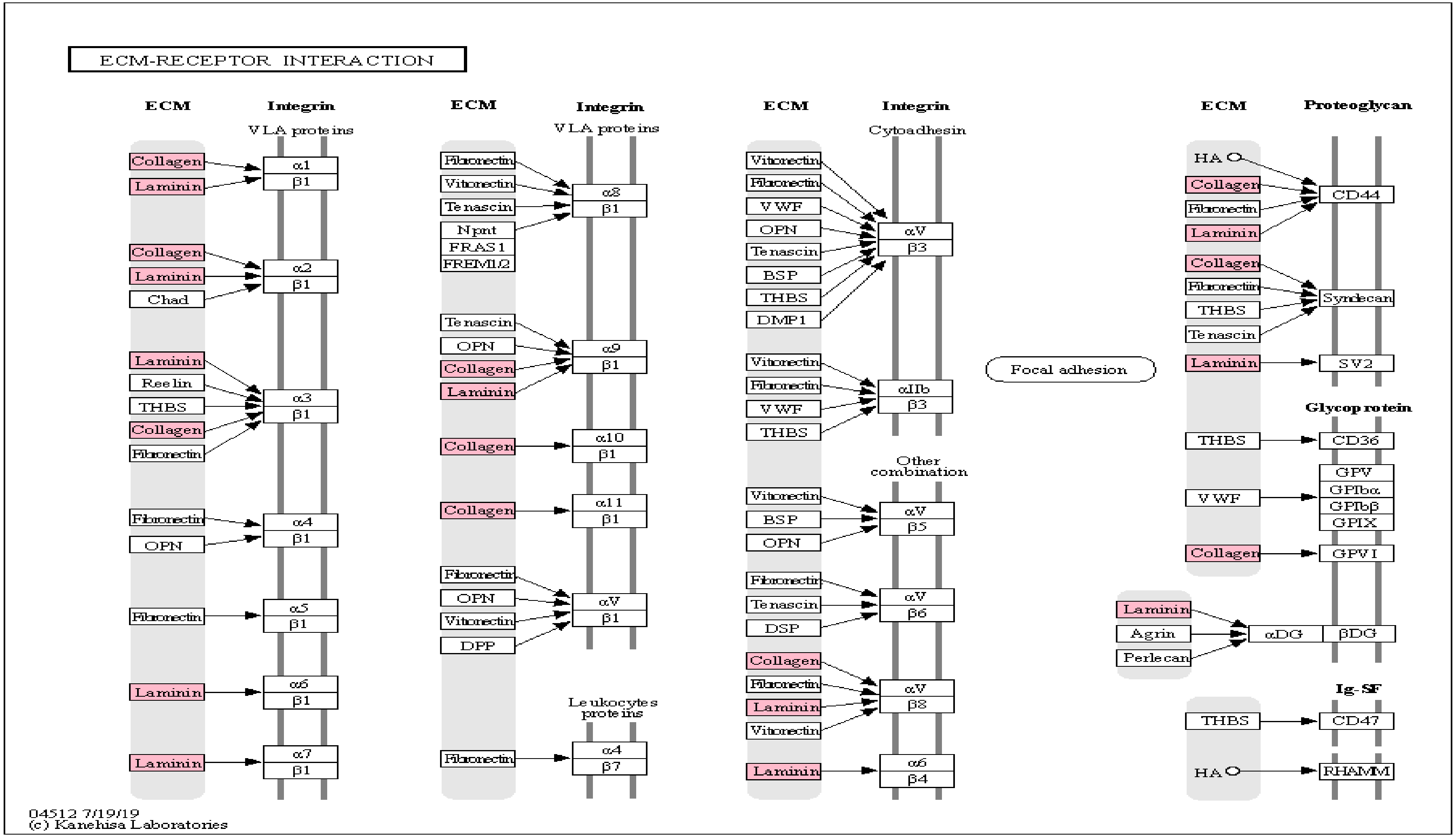

Supplemental Figure S7: KEGG ECM-receptor interaction (hsa04512) pathway. Red shaded area indicates genes which were upregulated from normal to leukoplakia to tumor.

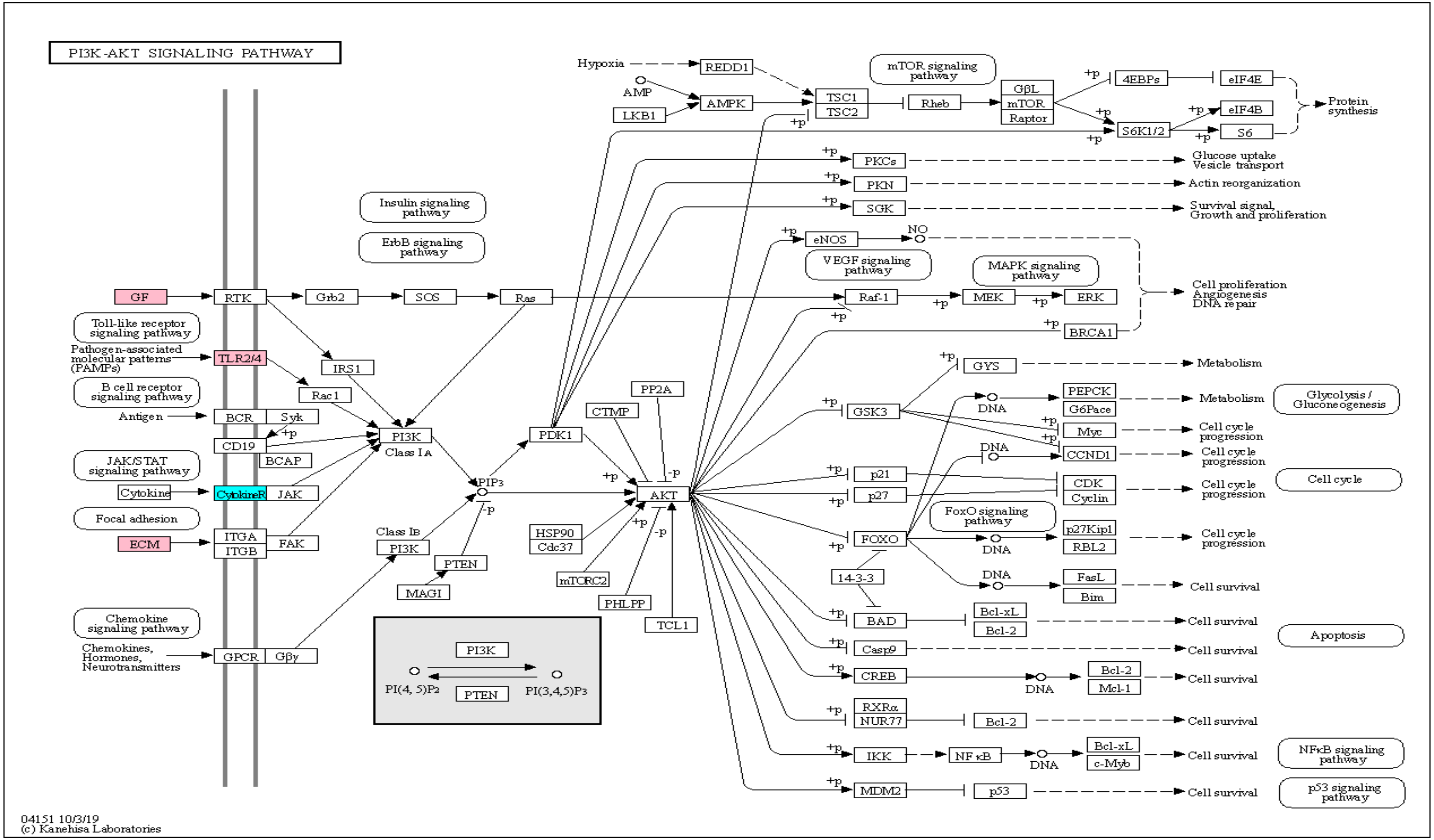

Supplemental Figure S8: KEGG PI3K-Akt signaling pathway (hsa04151) pathway. Red and blue shaded area indicate genes which were upregulated and downregulated, respectively, from normal to leukoplakia to tumor.

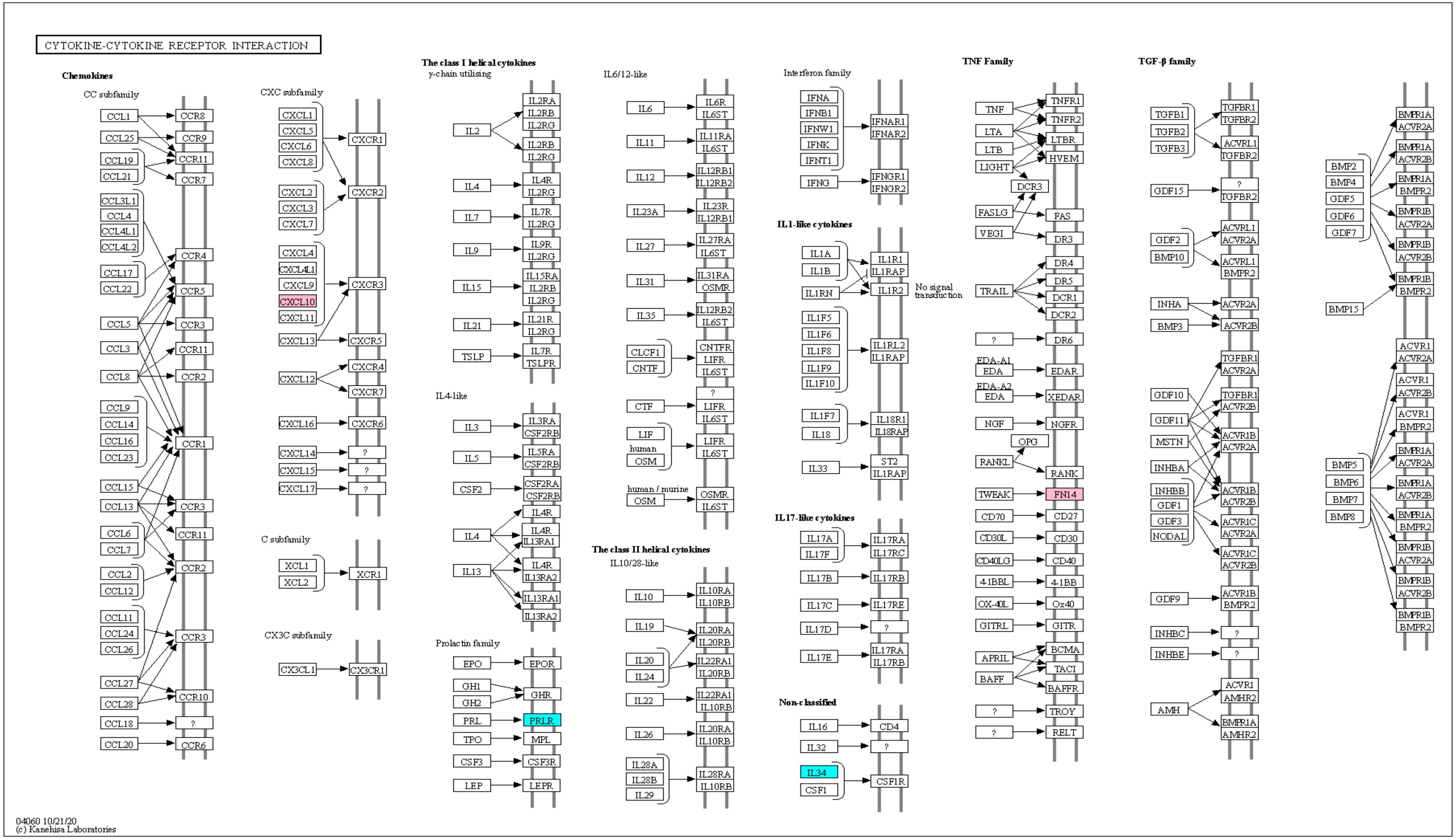

Supplemental Figure S9: KEGG cytokine-cytokine receptor interaction (hsa04060) pathway. Red and blue shaded area indicate genes which were upregulated and downregulated, respectively, from normal to leukoplakia to tumor.

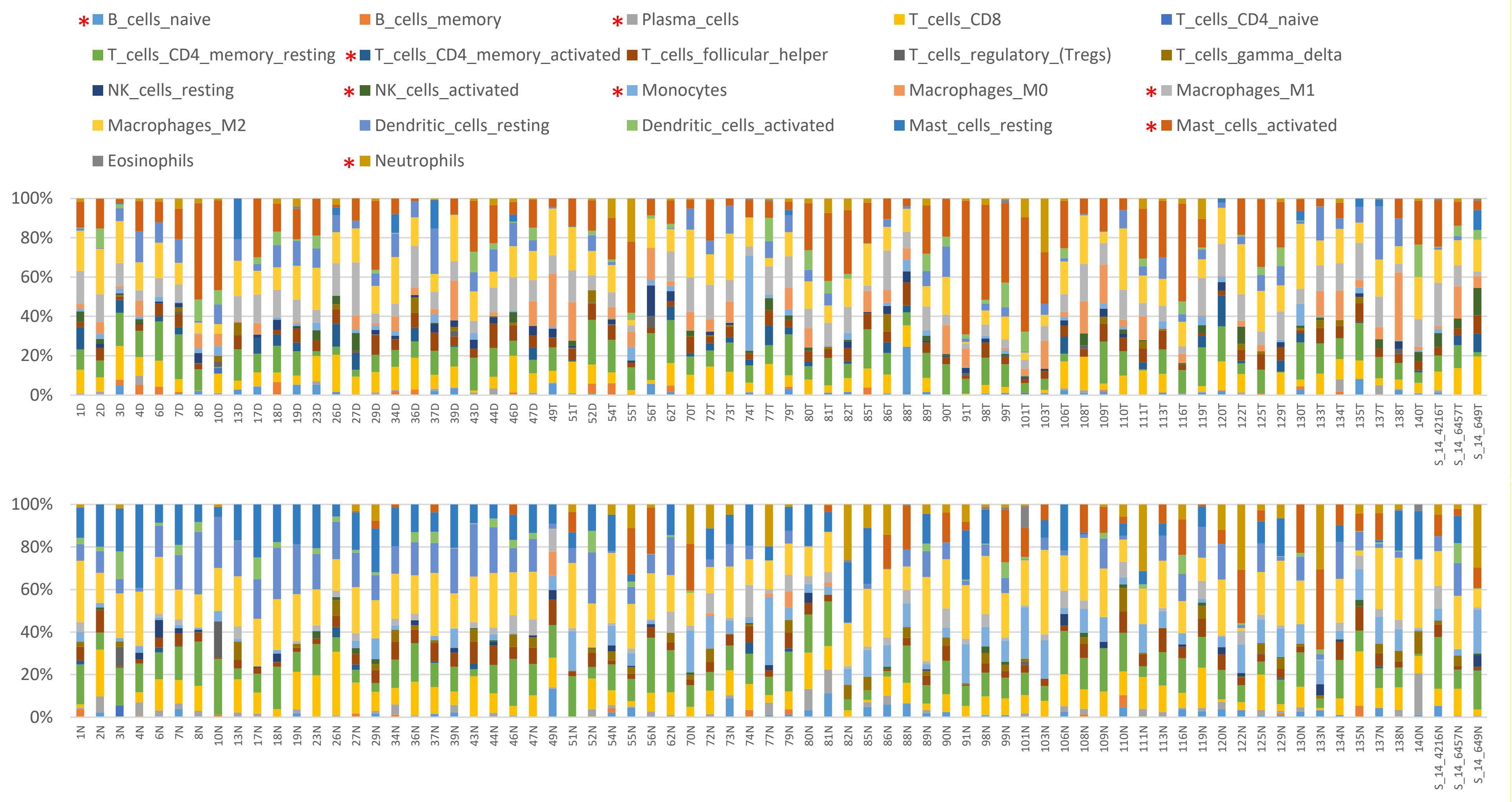

Supplemental Figure S10: The landscape of infiltrated immune cells in OSCC-GB patients. The y axes represent the relative proportions (in %) of 22 immune cell types in 72 tumor (top panel) and adjacent normal (bottom panel) samples. The x axes indicate corresponding tumor & normal sample ids. Marked (*) cells were found significantly dysregulated in tumor compared to paired normal tissue microenvironment.

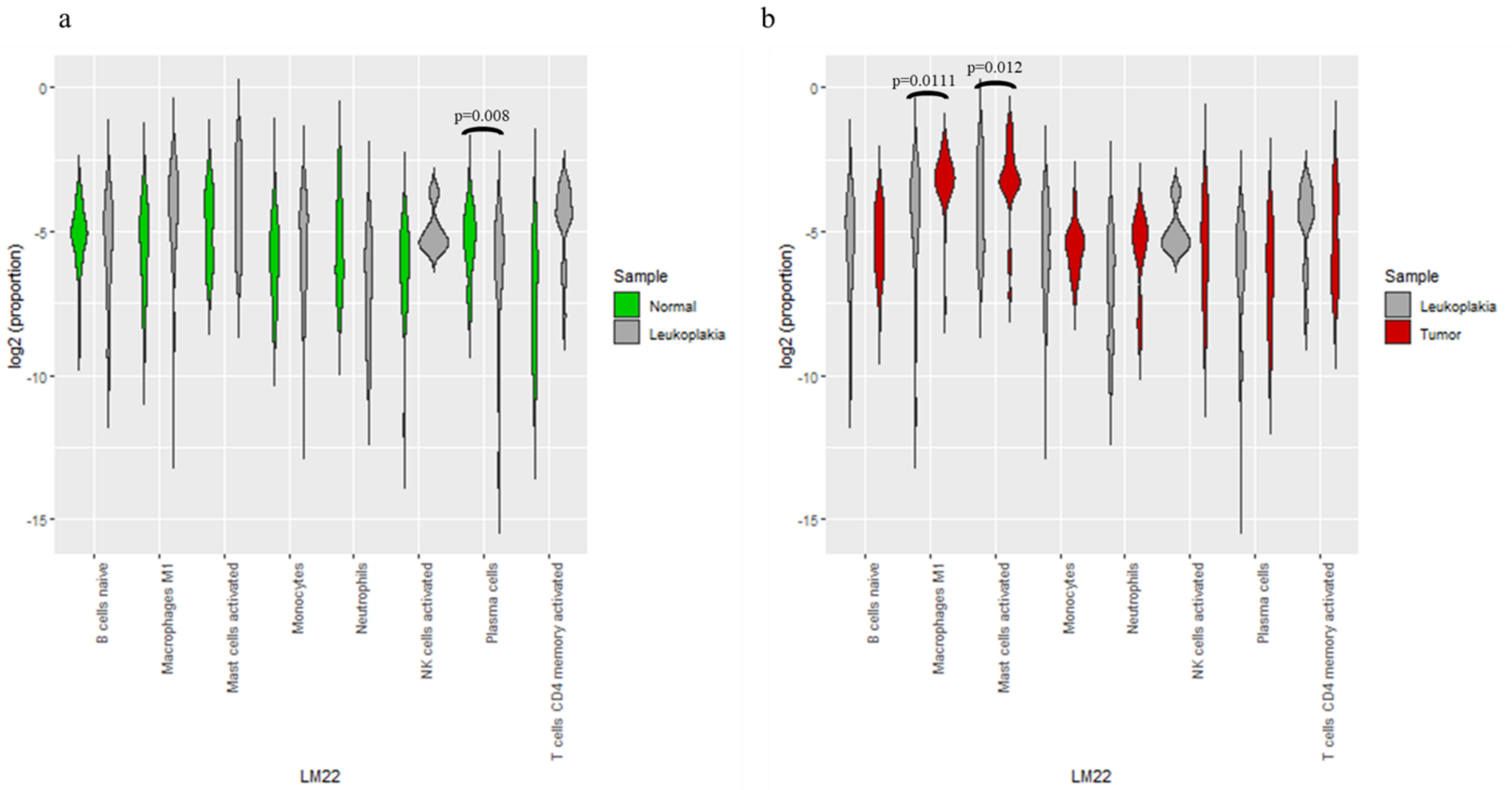

Supplemental Figure S11: Pairwise comparisons of infiltrating immune cells between (a) leukoplakia and normal tissues, and (b) tumor and leukoplakia tissues in OSCC-GB. Of the eight immune cell types, plasma cells show significant difference of proportions in leukoplakia compared to normal tissues whereas both macrophages M1 & mast cells activated show significant difference in tumor compared to leukoplakia tissues.

Supplemental Table S1: Information pertaining to tumor purity (ESTIMATE scores), and human papilloma virus (HPV) infection status in the tumor tissue of the 72 OSCC-GB patients

(Methodologies used to identify tumor purity & HPV infection status are provided in footnotes)

Sl. No. of Patient	Donor ID in clinical data	Patient ID	Centre	ESTIMATE Score	# of Reads after aligning unmapped reads to HPV genome that have MAPQ>0	# of Reads after aligning unmapped reads to HPV genome that have MAPQ=0	Presence of HPV
1	pt_49	I49	ACTREC	-208.16	0	10366272	Absent
2	pt_51	I51	ACTREC	1122.63	0	12172426	Absent
3	pt_54	I54	ACTREC	2088.38	0	14051442	Absent
4	pt_55	I55	ACTREC	-122.66	0	50174076	Absent
5	pt_56	I56	ACTREC	-1194.59	0	15386686	Absent
6	pt_62	I62	ACTREC	2510.75	0	15281250	Absent
7	pt_70	I70	ACTREC	672.83	0	10011034	Absent
8	pt_72	I72	ACTREC	2706.05	0	23134760	Absent
9	pt_73	I73	ACTREC	2094.20	0	9866512	Absent
10	pt_74	I74	ACTREC	4082.11	0	17914424	Absent
11	pt_77	I77	ACTREC	1201.80	0	25807138	Absent
12	pt_79	I79	ACTREC	3914.31	0	10084138	Absent
13	pt_80	I80	ACTREC	1277.67	0	11792802	Absent
14	pt_81	I81	ACTREC	623.91	0	29584338	Absent
15	pt_82	I82	ACTREC	1371.20	0	15733620	Absent
16	pt_85	I85	ACTREC	-914.13	0	10220718	Absent
17	pt_86	I86	ACTREC	2102.71	0	39928300	Absent
18	pt_88	I88	ACTREC	4098.34	0	16978032	Absent
19	pt_89	I89	ACTREC	2553.08	0	22551468	Absent
20	pt_90	I90	ACTREC	1224.33	0	16903858	Absent
21	pt_91	I91	ACTREC	199.44	0	20854268	Absent
22	pt_98	I98	ACTREC	397.53	0	40984390	Absent
23	pt_99	I99	ACTREC	1305.92	0	85536220	Absent
24	pt_101	I101	ACTREC	1495.94	0	45775582	Absent
25	pt_103	I103	ACTREC	519.42	0	19844954	Absent
26	pt_106	I106	ACTREC	-281.89	0	14835146	Absent
27	pt_108	I108	ACTREC	1705.95	0	11272482	Absent
28	pt_109	I109	ACTREC	3769.74	0	11479934	Absent
29	pt_110	I110	ACTREC	219.15	0	10535198	Absent
30	pt_111	I111	ACTREC	1295.21	0	12901970	Absent
31	pt_113	I113	ACTREC	1377.16	0	24795918	Absent

32	pt_116	I116	ACTREC	-613.94	0	21106390	Absent
33	pt_119	I119	ACTREC	2894.66	0	3685868	Absent
34	pt_120	I120	ACTREC	4663.45	0	18150704	Absent
35	pt_122	I122	ACTREC	1569.44	0	10611692	Absent
36	pt_125	I125	ACTREC	-563.08	0	10896770	Absent
37	pt_129	I129	ACTREC	1678.22	0	9938356	Absent
38	pt_130	I130	ACTREC	4121.70	0	11019366	Absent
39	pt_133	I133	ACTREC	1020.62	0	7585578	Absent
40	pt_134	I134	ACTREC	1964.95	0	11522114	Absent
41	pt_135	I135	ACTREC	1428.24	0	14933294	Absent
42	pt_137	I137	ACTREC	-572.94	0	9120536	Absent
43	pt_138	I138	ACTREC	-271.79	0	3697454	Absent
44	pt_140	I140	ACTREC	250.34	0	4627658	Absent
45	RADS_1	S1	RADCH	2462.09	0	9017974	Absent
46	RADS_2	S2	RADCH	266.28	0	10309814	Absent
47	RADS_3	S3	RADCH	3845.66	0	8078354	Absent
48	RADS_4	S4	RADCH	182.44	0	10333758	Absent
49	RADS_6	S6	RADCH	1418.69	0	8264644	Absent
50	RADS_7	S7	RADCH	863.44	0	8441608	Absent
51	RADS_8	S8	RADCH	-1185.21	0	6524110	Absent
52	RADS_10	S10	RADCH	-1274.89	0	5129868	Absent
53	RADS_13	S13	RADCH	838.91	0	24696458	Absent
54	RADS_17	S17	RADCH	1188.80	0	19724122	Absent
55	RADS_18	S18	RADCH	933.95	0	22465974	Absent
56	RADS_19	S19	RADCH	405.91	0	17516718	Absent
57	RADS_23	S23	RADCH	688.48	0	14515950	Absent
58	RADS_26	S26	RADCH	2898.01	0	13843008	Absent
59	RADS_27	S27	RADCH	1079.86	0	16631082	Absent
60	RADS_29	S29	RADCH	-1113.35	0	18966676	Absent
61	RADS_34	S34	RADCH	1484.95	0	18204418	Absent
62	RADS_36	S36	RADCH	1734.80	0	22806524	Absent
63	RADS_37	S37	RADCH	-1593.24	0	12523260	Absent
64	RADS_39	S39	RADCH	1089.19	0	13366666	Absent
65	RADS_43	S43	RADCH	0.09	0	22119856	Absent
66	RADS_44	S44	RADCH	-1743.64	0	28041910	Absent
67	RADS_46	S46	RADCH	204.26	0	11276940	Absent
68	RADS_47	S47	RADCH	595.28	0	19261510	Absent
69	RADS_52	S52	RADCH	261.84	0	9448808	Absent
70	S-14-4216	S_14_4216	CNCI	1183.67	0	11501104	Absent
71	S-14-6457	S_14_6457	CNCI	182.19	0	9406406	Absent
72	S-14-649	S_14_649	CNCI	484.65	0	11282162	Absent

Methods to identify tumor purity from RNA-Seq data

The raw read counts from the BAM file of 72 OSCC-GB tumor samples were generated using HTSeq-count (v. 0.12.4).[1] Tumor purity from the read counts were estimated using the R-based 'estimate' package.[2] Yoshihara K et al. have shown that the ESTIMATE scores can be used to infer tumour purity in tumor tissues. Thus we have computed the ESTIMATE score for each of the 72 tumor samples from our study. The ESTIMATE score for each of the 522 TCGA-HNSC tumor samples, generated using the RNA-seqV2 data, were downloaded from https://bioinformatics.mdanderson.org/estimate/. The ESTIMATE scores for 72 OSCC-GB tumor samples (our study) ranges between -1743.6 and 4663.5, whereas that for 522 HNSCC tumor samples (TCGA-HNSC) ranges between -3510.8 and 4227.7. Except for one sample i.e. I120T, ESTIMATE scores of all 71 OSCC-GB tumor samples lie within the ESTIMATE score range from the TCGA-HNSC tumor samples.

Methods to detect HPV infection status from RNA-Seq data

The RNA-Seq fastq reads from the 72 tumor samples were aligned to the human reference sequence (GRCh37) using HISAT2 aligner, which is splice-aware, and ideal to align RNA-seq data.[3] Fastq reads that did not map to the human genome were extracted. To detect presence of HPV reads, we aligned the unmapped fastq reads to HPV16 genome sequence downloaded from the NCBI database (Accession: NC_001526.4). MAPQ value of the alignment is defined as the probability that the reported mapped position is incorrect (http://samtools.github.io/hts-specs/SAMv1.pdf: SAM format specification.). The MAPQ value did not exceed zero for any of the tumors. MAPQ value of zero in HISAT2 aligner for a read signifies that either the read is unmapped, or it is multiple mapped and with mismatches or indels. Thus, no read that remained unaligned to the human genome mapped to the HPV16 genome.

For validation and control, we also aligned the unmapped reads against the genome of a known micro-organism associated with oral cancer, the bacteria *Porphyromonas gingivalis* (Accession: NZ_FQVS01000016).[4, 5] After alignment, we have found reads with MAPQ values > 0 in all patients, including MAPQ = 60 for some patients indicating uniquely mapped reads. Thus, for every patient, a subset of unaligned reads mapped to *Porphyromonas gingivalis* genome.

References

1. Anders S, Pyl PT, Huber W. HTSeq--a Python framework to work with high-throughput sequencing data. Bioinformatics. 2015; 31:166-169.
2. Yoshihara K, Shahmoradgoli M, Martínez E, Vegesna R, Kim H, Torres-Garcia W, Treviño V, Shen H, Laird PW, Levine DA, Carter SL, Getz G, Stemke-Hale K, Mills GB, Verhaak RG. Inferring tumour purity and stromal and immune cell admixture from expression data. Nat Commun. 2013; 4:2612.
3. Kim D, Langmead B, Salzberg SL. HISAT: a fast spliced aligner with low memory requirements. *Nat Methods*. 2015; 12:357-360.

4. Woo BH, Kim DJ, Choi JI, Kim SJ, Park BS, Song JM, Lee JH, Park HR. Oral cancer cells sustainedly infected with Porphyromonas gingivalis exhibit resistance to Taxol and have higher metastatic potential. Oncotarget. 2017; 8:46981-46992.
5. Qinyang Li, Yao Hu, Xuedong Zhou, Shiyu Liu, Qi Han, Lei Cheng, Role of Oral Bacteria in the Development of Oral Squamous Cell Carcinoma, *Cancers* 2020, **12**, 10, 2797.

Supplemental Table S2: Information on 72 gingivo-buccal oral cancer patients included in this study*

Sl. No.	Centre	Patient ID	OSCC-GB sub-cohort	Inclusion in Leukoplakia cohort
1	RADCH	S2	Discovery	Yes
2	RADCH	S6	Discovery	Yes
3	RADCH	S8	Discovery	Yes
4	RADCH	S10	Discovery	Yes
5	RADCH	S19	Discovery	Yes
6	RADCH	S26	Discovery	Yes
7	RADCH	S27	Discovery	Yes
8	RADCH	S29	Discovery	Yes
9	RADCH	S36	Discovery	Yes
10	RADCH	S37	Discovery	Yes
11	RADCH	S43	Discovery	Yes
12	RADCH	S47	Discovery	Yes
13	ACTREC	I54	Discovery	No
14	ACTREC	I55	Discovery	No
15	ACTREC	I56	Discovery	No
16	ACTREC	I62	Discovery	No
17	ACTREC	I70	Discovery	No
18	ACTREC	I79	Discovery	No
19	ACTREC	I80	Discovery	No
20	ACTREC	I81	Discovery	No
21	ACTREC	I88	Discovery	No
22	ACTREC	I90	Discovery	No
23	ACTREC	I91	Discovery	No
24	ACTREC	I98	Discovery	No
25	ACTREC	I99	Discovery	No
26	ACTREC	I103	Discovery	No
27	ACTREC	I106	Discovery	No
28	ACTREC	I108	Discovery	No
29	ACTREC	I119	Discovery	No
30	ACTREC	I120	Discovery	No
31	ACTREC	I129	Discovery	No
32	ACTREC	I130	Discovery	No
33	ACTREC	I134	Discovery	No
34	ACTREC	I135	Discovery	No
35	ACTREC	I140	Discovery	No
36	CNCI	S_14_649	Discovery	No
37	RADCH	S1	Validation	Yes
38	RADCH	S3	Validation	Yes

39	RADCH	S4	Validation	Yes
40	RADCH	S7	Validation	Yes
41	RADCH	S13	Validation	Yes
42	RADCH	S17	Validation	Yes
43	RADCH	S18	Validation	Yes
44	RADCH	S23	Validation	Yes
45	RADCH	S34	Validation	Yes
46	RADCH	S39	Validation	Yes
47	RADCH	S44	Validation	Yes
48	RADCH	S46	Validation	Yes
49	RADCH	S52	Validation	Yes
50	ACTREC	I49	Validation	No
51	ACTREC	I51	Validation	No
52	ACTREC	I72	Validation	No
53	ACTREC	I73	Validation	No
54	ACTREC	I74	Validation	No
55	ACTREC	I77	Validation	No
56	ACTREC	I82	Validation	No
57	ACTREC	I85	Validation	No
58	ACTREC	I86	Validation	No
59	ACTREC	I89	Validation	No
60	ACTREC	I101	Validation	No
61	ACTREC	I109	Validation	No
62	ACTREC	I110	Validation	No
63	ACTREC	I111	Validation	No
64	ACTREC	I113	Validation	No
65	ACTREC	I116	Validation	No
66	ACTREC	I122	Validation	No
67	ACTREC	I125	Validation	No
68	ACTREC	I133	Validation	No
69	ACTREC	I137	Validation	No
70	ACTREC	I138	Validation	No
71	CNCI	S_14_4216	Validation	No
72	CNCI	S_14_6457	Validation	No

*** All tumor samples were HPV negative**

Supplemental Table S3: Details on 1734 genes significantly differentially expressed in OSCC-GB patients

Gene symbol	p-value	Corrected p-value	Average log2 (fold-change)
AADAT	3E-06	4E-06	-1.0116
AAMDC	8E-06	1E-05	-1.2236
ABCA10	1E-13	2E-12	-2.1997
ABCA3	9E-11	4E-10	-2.2231
ABCA4	4E-10	1E-09	2.8103
ABCA8	3E-12	2E-11	-2.9721
ABCC2	2E-04	2E-04	1.7306
ABCC6	3E-06	4E-06	-1.6648
ABI3BP	6E-13	7E-12	-2.6373
ABL2	9E-11	4E-10	1.2219
ABLIM2	1E-03	2E-03	-1.3006
ACADS	1E-04	2E-04	-1.6839
ACADSB	7E-11	4E-10	-2.2983
ACBD4	4E-07	7E-07	-1.1292
ACHE	4E-05	5E-05	-2.9472
ACOT7	8E-10	3E-09	1.5568
ACOX2	8E-10	3E-09	-1.7629
ACP5	8E-09	2E-08	1.4457
ACPP	5E-06	7E-06	-1.8344
ACSM3	3E-12	3E-11	-2.5897
ACSS3	3E-09	7E-09	-2.7766
ACTN1	3E-12	2E-11	1.3089
ACVR2A	9E-13	1E-11	-1.2343
ACYP2	5E-05	7E-05	-1.1890
ADA	1E-14	4E-13	1.7669
ADAD2	9E-04	1E-03	-1.4753
ADAM12	3E-17	4E-15	4.0130
ADAM19	2E-07	3E-07	1.6776
ADAM22	6E-09	1E-08	-1.3409
ADAM32	3E-07	5E-07	-1.5539
ADAM33	1E-12	1E-11	-2.0733
ADAMTS12	7E-14	1E-12	3.0953
ADAMTS15	5E-06	7E-06	1.3298
ADAMTS2	2E-15	9E-14	2.7035
ADAMTS6	5E-07	8E-07	1.5795
ADAMTS7	2E-09	6E-09	1.6194
ADAMTSL1	3E-06	5E-06	-1.1294
ADAMTSL2	1E-08	3E-08	1.1486

ADAMTSL3	2E-12	2E-11	-2.8490
ADCK3	3E-06	5E-06	-3.2823
ADCY5	4E-13	5E-12	-2.4536
ADCY6	4E-11	2E-10	-1.3918
ADHFE1	2E-09	5E-09	-3.1111
ADORA3	1E-07	3E-07	1.1607
ADPRHL1	3E-04	4E-04	-2.3646
ADSSL1	6E-06	8E-06	-3.3925
ADTRP	4E-08	9E-08	2.1716
AFAP1L2	1E-08	2E-08	1.5802
AFF3	2E-06	2E-06	-1.8203
AGFG2	3E-07	5E-07	-2.3232
AGPHD1	2E-09	7E-09	-1.2191
AGRN	1E-12	1E-11	1.8189
AGTRAP	5E-15	2E-13	1.2292
AIM2	5E-08	1E-07	1.8016
AJAP1	1E-04	2E-04	2.5628
AJUBA	1E-06	2E-06	1.5103
AK8	3E-08	6E-08	-1.2848
AKAP12	3E-10	1E-09	-1.6879
AKAP6	2E-03	2E-03	-1.3399
AKAP7	1E-08	3E-08	-1.2772
ALDH1A1	1E-08	3E-08	-3.0812
ALDH3A1	2E-05	2E-05	-2.4342
ALDH3A2	2E-10	8E-10	-1.2407
ALDH3B1	4E-06	6E-06	-1.1595
ALDH6A1	2E-12	2E-11	-2.1512
ALG1L	9E-07	1E-06	1.9859
ALOX12	3E-07	6E-07	-2.8200
ALS2CR11	1E-07	2E-07	-1.4911
AMIGO1	2E-08	4E-08	-1.8748
AMIGO2	2E-09	6E-09	2.2282
AMOT	5E-10	2E-09	-3.3926
AMPD3	2E-07	4E-07	1.1242
AMY2B	5E-09	1E-08	-1.0454
ANG	1E-11	9E-11	-3.2714
ANGPT2	2E-09	7E-09	1.5672
ANGPTL1	1E-13	2E-12	-3.2063
ANGPTL6	1E-11	9E-11	1.2726
ANGPTL7	7E-07	1E-06	-2.3570
ANK1	3E-02	3E-02	-3.5051
ANK2	1E-10	5E-10	-2.8867

ANKRD23	1E-03	1E-03	-2.8033
ANKRD35	6E-08	1E-07	-1.4524
ANKRD6	3E-13	5E-12	-1.9377
ANLN	2E-07	3E-07	2.0568
ANO2	4E-08	8E-08	-1.3719
ANO5	3E-05	4E-05	-3.7244
ANO7	6E-03	6E-03	-2.1178
AOC3	1E-10	5E-10	-2.4532
AOX1	2E-10	1E-09	-2.7237
APBA2	3E-12	3E-11	2.4428
APBB1	4E-07	7E-07	-1.4404
APLF	8E-14	2E-12	-1.1806
APLN	6E-10	2E-09	2.2260
APOBEC3A	1E-04	1E-04	1.0482
APOBEC3G	8E-06	1E-05	1.2558
APOC1	4E-07	6E-07	1.8755
APOD	9E-13	1E-11	-3.2589
APOL1	5E-13	6E-12	2.5813
APOL2	3E-09	9E-09	1.2837
AQP1	4E-07	7E-07	-1.1029
AR	2E-12	2E-11	-2.9554
ARHGAP11A	4E-08	8E-08	1.4065
ARHGAP20	2E-16	2E-14	-2.1098
ARHGAP6	8E-11	4E-10	-1.9700
ARHGEF26	1E-19	1E-16	-3.1414
ARHGEF38	3E-05	4E-05	-5.1143
ARMCX2	5E-06	7E-06	-1.6597
ARMCX3	1E-04	1E-04	-1.4872
ARPC1B	5E-15	2E-13	1.6079
ARRDC4	4E-05	5E-05	1.6561
ARSG	1E-13	2E-12	-1.5550
ARSJ	2E-08	5E-08	1.6706
ASAP3	2E-11	1E-10	-1.5658
ASB16	6E-03	6E-03	-1.4968
ASB9	2E-05	3E-05	-2.1098
ASCL2	3E-09	1E-08	1.7383
ASPM	8E-07	1E-06	1.4388
ASRGL1	5E-03	6E-03	-1.6657
ATAD2	4E-08	9E-08	1.4342
ATF3	8E-04	9E-04	-1.2001
ATOH8	1E-07	2E-07	-2.5059
ATP1B3	1E-07	2E-07	1.1079

ATP6V0E2	4E-08	8E-08	-2.0288
ATP8A1	2E-05	3E-05	-1.5619
AUNIP	5E-12	4E-11	2.1468
AURKA	1E-13	2E-12	2.0514
B3GNT4	4E-06	6E-06	1.5227
B4GALNT1	3E-05	4E-05	1.3442
B4GALT6	1E-08	3E-08	-1.7276
BAK1	9E-14	2E-12	1.5766
BARX2	4E-08	9E-08	-2.1639
BASP1	2E-10	8E-10	1.4446
BATF2	8E-10	3E-09	3.3092
BBS5	7E-04	8E-04	-3.0232
BBS7	1E-10	5E-10	1.1930
BCAS1	1E-06	2E-06	-3.0420
BCAS4	3E-06	5E-06	1.1140
BCAT1	3E-08	6E-08	1.5864
BCKDHB	3E-11	2E-10	-1.4261
BEND6	5E-10	2E-09	1.9015
BEND7	3E-07	5E-07	-2.5435
BEX4	2E-15	9E-14	-2.3537
BFSP1	2E-05	3E-05	1.2172
BGN	3E-11	2E-10	1.3718
BHMT2	5E-09	1E-08	-2.3060
BICD1	3E-09	8E-09	1.0577
BIRC5	6E-09	2E-08	2.0346
BMP1	4E-12	3E-11	1.8872
BMP2	1E-05	2E-05	1.2560
BMP8A	3E-10	1E-09	1.9808
BMPER	3E-05	4E-05	-1.5738
BMX	2E-06	4E-06	-1.2964
BNC1	4E-06	6E-06	1.9222
BNC2	2E-07	3E-07	-2.4272
BOC	3E-14	7E-13	-1.9678
BOP1	1E-07	3E-07	1.2030
BORA	3E-11	2E-10	1.2880
BRCA1	1E-11	9E-11	1.3390
BRCA2	2E-08	5E-08	1.5246
BRIP1	8E-07	1E-06	1.3707
BSPRY	4E-03	4E-03	-1.1085
BST2	1E-14	3E-13	2.5902
BTN3A3	1E-06	2E-06	1.1137
BUB1	3E-09	7E-09	2.0014

C10orf55	9E-12	6E-11	1.7755
C10orf68	5E-08	1E-07	-1.5028
C11orf52	2E-07	5E-07	-2.4488
C11orf82	1E-11	8E-11	1.9422
C12orf75	1E-12	1E-11	2.3992
C14orf132	6E-12	5E-11	-1.8220
C14orf159	2E-06	3E-06	-1.3930
C14orf39	5E-03	6E-03	-2.2819
C15orf62	1E-07	2E-07	-1.1074
C16orf45	2E-06	2E-06	-1.4775
C16orf59	7E-10	2E-09	1.8648
C16orf74	3E-08	6E-08	1.7937
C16orf86	2E-07	3E-07	-1.3377
C17orf53	9E-09	2E-08	1.3993
C18orf54	2E-11	1E-10	1.2951
C19orf18	2E-10	9E-10	-1.7439
C19orf66	5E-14	1E-12	1.4612
C1orf115	9E-10	3E-09	-2.5621
C1QB	7E-06	1E-05	1.1951
C1QTNF6	1E-18	5E-16	3.0431
C1QTNF7	1E-11	8E-11	-1.4052
C1QTNF9B-AS1	2E-05	3E-05	-1.4633
C20orf197	4E-03	4E-03	2.6206
C2CD4B	6E-06	8E-06	-2.4693
C2orf88	1E-09	3E-09	-2.0419
C3orf18	3E-05	4E-05	-2.1630
C3orf79	3E-05	4E-05	-2.3501
C4BPA	7E-10	2E-09	-2.3395
C4orf19	6E-04	7E-04	-3.0255
C4orf48	4E-14	9E-13	2.1191
C5orf4	3E-10	1E-09	-2.1642
C6orf141	1E-08	3E-08	2.2482
C7orf10	3E-09	7E-09	1.8869
C7orf41	2E-08	5E-08	-1.7049
C8orf4	7E-03	8E-03	-1.3567
C8orf58	5E-06	7E-06	-1.0706
C9orf174	1E-06	2E-06	-1.0469
CA13	4E-10	2E-09	-1.8129
CA14	6E-07	1E-06	-2.3137
CA2	3E-04	4E-04	1.6269
CAB39L	1E-17	2E-15	-2.3951

CACNA1D	1E-07	2E-07	-2.2306
CACNA2D1	5E-08	1E-07	-2.5388
CACNA2D2	3E-06	4E-06	-1.8834
CACNB1	4E-03	5E-03	-2.1060
CADM1	3E-05	4E-05	-1.3226
CADPS2	2E-03	2E-03	-1.5838
CAMK4	1E-05	2E-05	1.4183
CAND2	2E-07	4E-07	-3.9185
CAP2	1E-02	1E-02	-2.2973
CAPN14	1E-05	2E-05	-2.4339
CAPN3	2E-05	3E-05	-2.2694
CAPN5	1E-07	2E-07	-1.6950
CAPS	1E-06	2E-06	-1.2732
CARD11	5E-07	9E-07	1.9176
CARNS1	3E-04	3E-04	-3.2307
CASC5	4E-08	9E-08	1.8243
CASP7	4E-11	2E-10	1.0863
CATSPERB	2E-02	2E-02	-1.5446
CAV1	2E-06	2E-06	1.2588
CBR3	3E-05	4E-05	-1.1504
CBX7	2E-10	8E-10	-1.8501
CCDC109B	3E-09	9E-09	1.2372
CCDC110	1E-07	2E-07	-2.4937
CCDC147	3E-03	3E-03	1.2146
CCDC149	2E-05	2E-05	-1.2529
CCDC80	3E-05	3E-05	-1.1428
CCL18	2E-03	2E-03	1.0311
CCL24	1E-03	1E-03	2.0035
CCL28	2E-03	2E-03	-4.1614
CCL5	5E-08	1E-07	1.6575
CCM2	1E-15	7E-14	1.2309
CCM2L	4E-06	6E-06	-1.0791
CCNB2	9E-10	3E-09	1.9581
CCND2	6E-04	7E-04	1.0861
CCNE1	8E-12	6E-11	1.5797
CCNF	5E-11	2E-10	1.6792
CCPG1	4E-03	4E-03	-1.0917
CCR4	6E-04	7E-04	1.2114
CCR7	1E-05	2E-05	1.4633
CCRL1	6E-04	7E-04	1.8447
CCRL2	8E-07	1E-06	1.0545
CCRN4L	2E-09	5E-09	1.4395

CCSER1	3E-07	5E-07	-2.0337
CCT6B	9E-12	6E-11	-1.5143
CD1A	1E-03	1E-03	-1.0470
CD2	9E-05	1E-04	1.2489
CD207	8E-04	9E-04	-1.5330
CD247	9E-07	2E-06	1.4109
CD274	8E-08	2E-07	2.0560
CD276	2E-16	1E-14	1.7987
CD300A	5E-05	6E-05	1.1246
CD300LF	1E-06	2E-06	1.3538
CD34	7E-08	1E-07	-1.1920
CD36	5E-05	6E-05	-1.3815
CD38	9E-05	1E-04	1.4600
CD3D	7E-05	9E-05	1.2403
CD3G	7E-05	9E-05	1.2135
CD72	2E-06	3E-06	1.6450
CD80	2E-12	2E-11	2.9286
CD86	2E-07	3E-07	1.4160
CD8A	5E-04	6E-04	1.2989
CDC20	8E-10	3E-09	2.4749
CDC25A	9E-09	2E-08	1.3308
CDC25B	1E-14	4E-13	2.0266
CDC42EP5	1E-04	1E-04	-1.6395
CDC45	3E-11	2E-10	1.9859
CDC6	9E-12	6E-11	2.3124
CDC7	1E-09	4E-09	1.3023
CDCA2	2E-08	5E-08	1.7714
CDCA3	3E-09	9E-09	1.9644
CDCA4	6E-09	2E-08	1.4545
CDCA5	5E-11	2E-10	2.4664
CDCA7	2E-05	2E-05	1.2122
CDCA8	6E-09	1E-08	1.7924
CDH11	2E-08	5E-08	1.5981
CDH24	2E-08	5E-08	1.3349
CDH3	4E-08	9E-08	2.7334
CDK1	4E-09	1E-08	1.6807
CDK18	2E-05	3E-05	1.3517
CDK20	1E-08	3E-08	-1.0914
CDK6	3E-10	1E-09	1.4262
CDKN3	1E-12	1E-11	1.9507
CDON	1E-13	2E-12	-1.7921
CDR1	5E-09	1E-08	-3.0097

CDT1	3E-07	6E-07	1.4165
CEACAM1	9E-07	1E-06	-1.9913
CEACAM19	5E-04	5E-04	1.2203
CEACAM5	1E-02	1E-02	-1.1289
CELSR3	1E-09	5E-09	2.1529
CENPF	8E-08	2E-07	1.5418
CENPI	6E-11	3E-10	1.9752
CENPK	1E-07	2E-07	1.6086
CENPL	2E-11	1E-10	1.1400
CEP55	4E-11	2E-10	2.4068
CERCAM	2E-11	1E-10	1.3355
CES1	8E-10	3E-09	-2.0953
CETP	1E-11	8E-11	1.4300
CFD	3E-13	5E-12	-3.0518
CFL2	2E-02	2E-02	-1.9750
CGNL1	3E-13	5E-12	-3.3488
CHADL	6E-14	1E-12	-2.1912
CHD3	2E-02	2E-02	-1.0537
CHEK1	2E-10	8E-10	1.7252
CHN1	2E-12	2E-11	1.9984
CHPT1	2E-09	6E-09	-2.4352
CHRNA10	4E-04	4E-04	-3.1791
CHST1	5E-07	9E-07	1.8265
CHST11	8E-14	1E-12	1.9216
CHST2	9E-08	2E-07	2.0416
CHTF18	1E-08	3E-08	1.2565
CIDEC	3E-09	7E-09	-1.9268
CKAP2	1E-11	8E-11	1.4137
CKAP2L	2E-08	5E-08	2.0598
CKMT2	2E-05	2E-05	-4.2274
CKS2	2E-10	1E-09	1.4393
CLCA2	2E-03	2E-03	1.2808
CLCN4	2E-06	4E-06	-2.8912
CLDN11	2E-10	7E-10	-2.5039
CLDN14	1E-06	2E-06	2.0052
CLDN5	4E-06	5E-06	-1.6786
CLDN7	2E-03	2E-03	-1.8687
CLEC3B	2E-15	8E-14	-3.3163
CLEC7A	3E-07	5E-07	1.1700
CLIC5	8E-04	9E-04	-2.9450
CLIC6	1E-07	2E-07	-2.1051
CLMN	4E-05	5E-05	-1.3569

CLYBL	1E-11	9E-11	-1.7208
CMA1	3E-07	6E-07	-1.3702
CMBL	3E-03	4E-03	-1.9122
CMPK2	5E-11	3E-10	2.5021
CMYA5	2E-04	3E-04	-3.2154
CNN1	4E-05	5E-05	-2.4584
CNTNAP3	2E-04	2E-04	-1.1194
CNTNAP3B	4E-11	2E-10	-1.4292
COBL	5E-10	2E-09	-2.6468
COL10A1	1E-08	3E-08	2.9589
COL12A1	6E-15	2E-13	2.0786
COL13A1	1E-08	3E-08	1.6407
COL14A1	3E-08	7E-08	-1.2956
COL16A1	4E-11	2E-10	1.6099
COL17A1	2E-04	3E-04	1.4669
COL1A1	7E-12	5E-11	2.1756
COL21A1	6E-10	2E-09	-2.0595
COL22A1	4E-07	8E-07	4.1427
COL27A1	3E-11	2E-10	1.6440
COL3A1	2E-11	1E-10	1.7048
COL4A1	1E-17	2E-15	2.5479
COL4A2	1E-15	6E-14	2.0848
COL4A5	9E-13	1E-11	2.2158
COL4A6	1E-16	1E-14	3.4224
COL5A1	6E-12	5E-11	2.5575
COL5A2	1E-14	4E-13	2.5348
COL5A3	5E-07	8E-07	1.7834
COL6A1	2E-08	5E-08	1.3548
COL6A3	5E-10	2E-09	1.5341
COL6A6	6E-08	1E-07	-2.0031
COL7A1	1E-07	3E-07	1.6915
COL8A1	5E-10	2E-09	2.1406
COL9A3	2E-03	2E-03	-1.0556
COQ10A	6E-04	7E-04	-2.6627
CORO2B	1E-08	3E-08	-1.4135
COTL1	1E-10	5E-10	1.3214
COX14	7E-06	1E-05	-1.2026
COX7A1	1E-07	3E-07	-3.4601
CP	8E-07	1E-06	-2.5299
CPAMD8	3E-11	2E-10	-3.2850
CPD	2E-03	2E-03	-1.3202
CPE	1E-04	1E-04	-1.8431

CPEB1	5E-11	2E-10	-2.5411
CPEB3	5E-11	3E-10	-1.6151
CPED1	1E-07	2E-07	-1.5585
CPNE5	5E-03	5E-03	1.0104
CPXM1	3E-11	1E-10	2.4310
CRAT	1E-08	3E-08	-2.3114
CREB3L4	2E-05	3E-05	-2.7726
CRY2	2E-10	1E-09	-1.3466
CRYAB	1E-07	2E-07	-2.2024
CRYM	1E-05	2E-05	-3.6771
CSPG4	2E-07	3E-07	1.5524
CSRNP3	1E-09	4E-09	-2.2010
CTHRC1	3E-12	3E-11	2.6000
CTPS1	3E-09	8E-09	1.0021
CTSC	3E-12	2E-11	1.5563
CTSF	3E-09	1E-08	-2.0290
CTSL1	8E-09	2E-08	1.2330
CTSL2	6E-06	8E-06	2.3485
CTTNBP2	6E-18	1E-15	-2.8366
CX3CR1	5E-05	7E-05	-1.0203
CXCL1	2E-09	7E-09	2.3194
CXCL10	8E-11	4E-10	3.4584
CXCL11	2E-10	7E-10	4.4642
CXCL12	2E-08	4E-08	-1.5427
CXCL9	3E-10	1E-09	3.0192
CXCR2	2E-07	4E-07	-1.8210
CXCR3	1E-04	2E-04	1.3740
CYBRD1	5E-09	1E-08	-1.2735
CYP26B1	3E-06	4E-06	1.0001
CYP27A1	2E-09	6E-09	-2.1858
CYP27B1	3E-12	2E-11	3.5956
CYP2J2	6E-11	3E-10	-2.1552
CYP2R1	7E-10	2E-09	-1.1706
CYP3A5	7E-05	9E-05	-1.4602
CYP4F3	2E-05	2E-05	-1.5652
CYP4X1	4E-10	1E-09	-2.8826
DAGLA	5E-08	1E-07	-1.7208
DARC	1E-07	2E-07	-1.4621
DBF4B	2E-09	5E-09	1.2182
DBNDD1	9E-10	3E-09	-2.0053
DCBLD1	4E-17	4E-15	2.3555
DCBLD2	6E-06	9E-06	1.3952

DCLK1	3E-09	9E-09	-1.8918
DCLRE1B	1E-11	9E-11	1.1211
DDAH1	1E-06	2E-06	-1.5188
DDIT4	4E-08	9E-08	1.4462
DDO	1E-03	2E-03	-2.0633
DDX58	9E-10	3E-09	1.1815
DDX60	3E-13	4E-12	2.2635
DDX60L	1E-12	1E-11	1.9806
DENND2A	2E-06	3E-06	-1.0957
DEPDC1	3E-08	6E-08	1.7355
DEPDC1B	5E-10	2E-09	2.0288
DEPTOR	1E-12	1E-11	-3.4676
DFNA5	1E-17	2E-15	2.7142
DGCR6	2E-04	2E-04	-2.3962
DIAPH3	6E-07	1E-06	1.5581
DIO3	5E-02	5E-02	-1.1436
DKK2	1E-07	2E-07	-1.4847
DKK3	2E-05	3E-05	1.1725
DLEU7	7E-10	2E-09	1.5496
DLG2	1E-14	4E-13	-2.6417
DLGAP3	3E-03	3E-03	2.9290
DLGAP4	2E-08	5E-08	1.1143
DLGAP5	9E-09	2E-08	2.1634
DLX1	5E-06	7E-06	1.5408
DMD	2E-04	3E-04	-1.8595
DNA2	3E-09	9E-09	1.1160
DNAH17	1E-10	6E-10	3.0363
DNALI1	3E-06	5E-06	-1.6997
DNER	1E-02	1E-02	1.7355
DNMT1	9E-11	4E-10	1.1796
DNMT3B	1E-11	8E-11	2.4733
DOK5	9E-03	1E-02	-1.7370
DOK7	2E-03	2E-03	-2.1114
DOPEY2	1E-07	2E-07	-1.1386
DPF3	2E-02	3E-02	-2.1344
DPT	2E-14	5E-13	-3.2103
DPY19L3	1E-02	1E-02	-1.0193
DRAM1	4E-09	1E-08	1.4701
DSE	1E-08	2E-08	1.2029
DTL	9E-11	4E-10	1.8450
DTNA	8E-07	1E-06	-3.4244
DTX3L	6E-15	2E-13	1.3997

DUSP1	1E-06	2E-06	-1.0825
DYRK1B	6E-09	2E-08	-2.1879
EBF1	4E-07	7E-07	-1.1425
EBF2	3E-05	3E-05	-1.4436
EBI3	2E-05	2E-05	1.4053
ECHDC2	4E-12	3E-11	-1.8700
ECHDC3	1E-07	2E-07	-1.7856
ECT2	5E-08	1E-07	1.2396
EDA	3E-09	7E-09	-1.2386
EDIL3	3E-08	6E-08	1.4834
EDNRA	9E-08	2E-07	1.3054
EDNRB	6E-09	1E-08	-1.3329
EEPD1	2E-07	3E-07	-1.1320
EFCAB4A	5E-04	6E-04	-3.3900
EFCAB6	3E-14	7E-13	-2.3723
EFHA2	8E-10	3E-09	-2.1011
EFHD1	2E-06	3E-06	-1.7771
EFNB1	2E-10	7E-10	1.6111
EGFL6	3E-09	8E-09	2.1872
EGR1	2E-05	3E-05	-1.5364
EHD3	3E-03	3E-03	-1.0080
EIF2AK2	1E-13	2E-12	1.6722
EIF5A2	2E-10	7E-10	1.7983
ELF3	2E-02	3E-02	-1.2580
ELF4	1E-09	4E-09	1.1897
ELN	1E-07	2E-07	-1.8232
EMCN	6E-07	1E-06	-1.1709
EME1	1E-10	5E-10	1.8271
EMP1	1E-06	2E-06	-1.9113
EMR2	1E-09	4E-09	1.5582
ENAH	1E-11	8E-11	1.5873
ENDOU	1E-04	2E-04	-2.2787
ENO3	3E-04	3E-04	-3.9180
ENPP4	1E-05	2E-05	-2.1631
EOMES	2E-05	2E-05	1.4155
EPB41L4A	4E-13	6E-12	-1.6390
EPB49	9E-10	3E-09	-1.4145
EPDR1	2E-03	2E-03	-1.8795
EPHB2	2E-09	5E-09	2.0677
EPHB6	3E-06	5E-06	-1.2862
EPHX1	2E-11	1E-10	-1.2361
EPHX2	1E-11	8E-11	-1.8231

EPPK1	8E-04	9E-04	1.0662
EPS8	8E-09	2E-08	-1.0320
EPSTI1	5E-13	7E-12	2.6173
ERCC6L	1E-09	4E-09	1.5168
ERVMER34-1	5E-05	6E-05	1.2272
ESCO2	2E-06	3E-06	1.4056
ETNK2	1E-08	3E-08	-3.3895
ETS1	1E-09	4E-09	1.1776
ETV1	2E-02	2E-02	-2.5240
ETV2	9E-04	1E-03	-1.0031
ETV4	1E-04	1E-04	1.1495
ETV7	3E-07	5E-07	1.8139
EVA1A	1E-09	4E-09	2.8927
EYA2	3E-10	1E-09	-2.1856
F2RL1	4E-05	5E-05	1.2656
F2RL2	4E-08	1E-07	1.8583
F3	1E-02	1E-02	1.0400
F8	2E-05	2E-05	-1.2686
FABP3	4E-03	4E-03	-3.2408
FADD	3E-07	5E-07	1.4488
FAIM2	3E-11	1E-10	-2.3160
FAIM3	1E-08	3E-08	2.2032
FAM107A	3E-13	4E-12	-2.9493
FAM110B	5E-08	1E-07	-1.9893
FAM110D	2E-06	4E-06	-1.1150
FAM111B	1E-07	3E-07	1.7196
FAM134B	8E-05	1E-04	-1.8490
FAM13A	6E-06	9E-06	-1.0961
FAM13C	9E-08	2E-07	-1.5536
FAM171A1	1E-08	3E-08	-1.3543
FAM171B	1E-04	1E-04	-1.3566
FAM174B	9E-10	3E-09	-2.4991
FAM180A	3E-06	5E-06	-1.4514
FAM184A	2E-10	7E-10	-2.6051
FAM188B	4E-05	5E-05	-1.1190
FAM189A2	2E-11	1E-10	-2.7393
FAM198A	3E-09	8E-09	-1.1849
FAM19A5	3E-07	6E-07	-1.0373
FAM228B	5E-08	1E-07	-1.7654
FAM229B	3E-07	6E-07	-1.5970
FAM26F	7E-08	1E-07	2.0013
FAM47E	3E-04	4E-04	-2.3821

FAM49B	2E-14	4E-13	1.1583
FAM63A	2E-10	7E-10	-1.1905
FAM64A	6E-06	8E-06	1.4534
FAM71E1	7E-08	1E-07	-1.4641
FAM72A	3E-02	3E-02	1.2189
FAM83A	1E-05	2E-05	2.3471
FAM89A	4E-08	8E-08	1.5052
FANCA	6E-09	2E-08	1.2166
FANCI	1E-09	4E-09	1.5772
FAP	6E-12	5E-11	2.5671
FBLIM1	5E-11	3E-10	1.7576
FBLN1	5E-10	2E-09	-1.2517
FBLN5	3E-06	5E-06	-1.1654
FBN2	7E-09	2E-08	2.7173
FBXL8	6E-07	1E-06	-1.0614
FBXO2	1E-03	2E-03	1.1215
FBXO39	7E-14	1E-12	2.4339
FBXO6	1E-11	7E-11	1.3413
FCER1A	3E-10	1E-09	-2.0416
FCER1G	1E-06	2E-06	1.4169
FCGBP	3E-04	4E-04	-2.9373
FCGR3A	5E-10	2E-09	2.3390
FEN1	6E-12	5E-11	1.4937
FERMT1	5E-06	7E-06	1.5979
FEZ1	6E-14	1E-12	2.2158
FGF12	1E-04	2E-04	-1.7734
FGF13	3E-07	5E-07	-2.4413
FGF18	1E-07	2E-07	-1.7271
FGF2	2E-08	5E-08	-1.3324
FGGY	1E-05	2E-05	-1.8653
FHIT	6E-12	4E-11	-2.1410
FHL1	1E-05	2E-05	-2.7904
FHL5	6E-09	2E-08	-2.5493
FIBIN	2E-05	2E-05	-1.3520
FILIP1	3E-03	3E-03	-2.2672
FITM1	7E-04	9E-04	-4.3518
FJX1	8E-11	4E-10	1.9784
FLNC	3E-02	3E-02	-2.6850
FLRT2	5E-07	9E-07	1.6874
FLRT3	2E-10	9E-10	3.0851
FMNL2	4E-13	6E-12	1.3854
FMNL3	2E-12	2E-11	1.3206

FMO2	9E-14	2E-12	-3.7006
FMO5	4E-05	5E-05	-2.5247
FN1	2E-09	5E-09	2.0743
FNDC1	4E-05	5E-05	2.0081
FNDC4	2E-07	5E-07	-1.6334
FNDC5	3E-07	5E-07	-4.7320
FOS	5E-04	6E-04	-1.1648
FOSB	1E-06	2E-06	-1.9493
FOXC2	1E-04	1E-04	1.2909
FOXM1	4E-09	1E-08	2.0825
FOXN2	2E-11	1E-10	1.0393
FOXO4	4E-11	2E-10	-1.1647
FPR3	6E-08	1E-07	1.6340
FREM1	5E-12	4E-11	-2.0648
FRMD3	2E-05	2E-05	-2.6114
FRZB	7E-06	1E-05	-2.4315
FSCN1	5E-11	2E-10	2.1860
FST	8E-11	4E-10	2.7767
FSTL3	4E-11	2E-10	2.0370
FXYD2	8E-11	4E-10	-2.8061
FXYD5	7E-13	9E-12	1.7088
FYB	1E-05	2E-05	1.2500
FZD4	8E-07	1E-06	-1.7069
FZD6	1E-08	3E-08	1.4017
FZD7	1E-07	3E-07	-1.0871
GALNS	9E-11	4E-10	1.0793
GALNT12	1E-10	6E-10	-3.5105
GALNT18	5E-12	4E-11	1.4396
GALNT5	1E-04	2E-04	-2.6676
GAMT	8E-08	2E-07	-2.8526
GAS2	2E-07	3E-07	-2.5153
GAS7	2E-09	6E-09	-1.4390
GATM	2E-15	8E-14	-2.9191
GATS	4E-05	5E-05	-1.7043
GBP1	2E-12	2E-11	2.2639
GBP4	3E-10	1E-09	2.9209
GCNT2	3E-08	7E-08	-1.7200
GCNT3	4E-04	5E-04	-3.5800
GDPD2	3E-06	5E-06	2.8166
GFI1	5E-09	1E-08	1.7842
GFRA1	2E-11	1E-10	-3.0210
GGT6	2E-02	2E-02	-1.3572

GHR	6E-08	1E-07	-1.5710
GINS4	4E-09	1E-08	1.4518
GJA1	8E-05	1E-04	1.0658
GJC1	3E-12	3E-11	2.2649
GKAP1	2E-09	5E-09	-2.4055
GLI3	2E-09	6E-09	1.3292
GLIPR1	5E-13	7E-12	1.6237
GLIS1	8E-04	9E-04	1.7436
GLRB	4E-06	6E-06	-3.2853
GLS2	3E-09	1E-08	-1.6678
GLT25D1	3E-12	2E-11	1.3324
GLTSCR2	3E-15	1E-13	-1.0594
GLUD2	9E-07	2E-06	-1.3754
GM2A	3E-06	5E-06	1.0536
GMNN	3E-10	1E-09	1.3276
GMPR	4E-05	5E-05	-2.9204
GNA12	1E-16	1E-14	1.1830
GNA14	4E-07	7E-07	-1.2992
GNAZ	2E-09	7E-09	-1.5722
GNE	2E-04	2E-04	-1.2789
GNG7	1E-05	2E-05	-1.9856
GNLY	3E-09	8E-09	2.7724
GOLGA7B	4E-08	8E-08	2.3820
GPAM	5E-06	8E-06	-1.4754
GPAT2	1E-03	1E-03	-1.1207
GPD1L	1E-11	7E-11	-2.5075
GPR133	5E-08	1E-07	-1.5509
GPR143	3E-04	3E-04	-1.4435
GPR153	2E-09	6E-09	1.8139
GPR171	5E-05	6E-05	1.3924
GPR176	4E-12	3E-11	2.0956
GPR39	1E-07	2E-07	2.2635
GPR64	4E-10	2E-09	-2.9357
GPR68	6E-09	2E-08	1.2359
GPR82	6E-04	7E-04	1.0253
GPRASP1	8E-12	5E-11	-2.0496
GPRC5B	6E-08	1E-07	-2.1972
GPRC5C	3E-04	4E-04	-1.1581
GPRIN2	6E-04	7E-04	-1.7708
GPT2	5E-09	1E-08	-1.5124
GPX3	5E-12	4E-11	-2.4660
GRIK5	3E-06	5E-06	-1.0756

GSG2	4E-11	2E-10	1.7496
GSN	1E-09	3E-09	-1.3717
GTF2IRD2B	1E-09	4E-09	-1.0786
GUCY2C	3E-10	1E-09	-1.2295
GULP1	4E-17	5E-15	-2.5943
GYLTL1B	5E-04	6E-04	1.5080
GZMB	3E-09	8E-09	2.3867
H2AFX	3E-08	6E-08	1.2074
HAAO	2E-12	2E-11	-1.5509
HADH	2E-09	5E-09	-1.5931
HAGHL	3E-08	7E-08	1.1820
HAP1	5E-04	6E-04	2.0227
HAVCR2	1E-06	2E-06	1.5138
HBB	9E-04	1E-03	-1.4800
HELLS	6E-07	1E-06	1.2255
HELZ2	6E-13	7E-12	1.7557
HENMT1	4E-11	2E-10	1.5856
HEPHL1	4E-07	7E-07	1.7512
HERC5	5E-09	1E-08	1.9621
HERC6	7E-07	1E-06	1.4926
HID1	5E-04	6E-04	-2.5510
HIST1H2AB	1E-09	4E-09	1.5467
HIST1H2AI	1E-06	2E-06	1.3429
HIST1H2AK	9E-08	2E-07	1.4203
HIST1H2AM	2E-07	4E-07	1.4431
HIST1H2BB	1E-08	2E-08	1.4700
HIST1H2BF	5E-08	1E-07	1.5707
HIST1H2BH	4E-07	8E-07	1.3989
HIST1H2BJ	2E-09	6E-09	1.4005
HIST1H2BL	9E-07	1E-06	1.4583
HIST1H2BN	1E-06	2E-06	1.2768
HIST1H2BO	5E-09	1E-08	1.9023
HIST1H3C	1E-07	2E-07	1.6054
HIST1H4I	9E-07	2E-06	1.5743
HJURP	1E-09	4E-09	2.1104
HKR1	1E-07	2E-07	-1.1221
HLF	1E-17	2E-15	-3.3267
HMGB3	3E-10	1E-09	1.6866
HNMT	3E-04	4E-04	-1.0660
HOMER3	2E-12	2E-11	1.6194
HOPX	6E-04	7E-04	-1.2434
HOXB3	2E-07	3E-07	1.2754

HOXD10	2E-08	4E-08	2.3958
HPDL	2E-10	9E-10	-1.8590
HPGD	2E-09	5E-09	-1.3491
HSD11B2	8E-04	9E-04	-1.1326
HSD17B6	5E-10	2E-09	2.2251
HSF2BP	2E-09	5E-09	1.7367
HSPA12B	1E-05	2E-05	-1.0751
HSPB6	2E-07	4E-07	-3.6083
HSPB7	2E-05	3E-05	-3.0637
HSPB8	1E-07	3E-07	-1.8770
HTR7	1E-15	6E-14	3.5850
ICA1	2E-08	6E-08	-2.5846
ICA1L	7E-12	5E-11	-1.6707
ICAM5	2E-05	2E-05	1.6279
ICOS	3E-10	1E-09	2.4891
ID4	5E-17	5E-15	-2.2312
IDO1	1E-11	7E-11	3.7633
IER5L	1E-11	9E-11	1.4316
IFI27	3E-10	1E-09	1.6866
IFI30	3E-11	2E-10	1.8247
IFI35	3E-12	3E-11	2.1241
IFI44	5E-13	7E-12	2.2518
IFI6	1E-11	8E-11	2.0715
IFIH1	2E-12	2E-11	1.8764
IFIT1	5E-10	2E-09	1.6830
IFIT2	8E-06	1E-05	1.8044
IFIT3	1E-11	8E-11	2.4998
IFITM1	3E-09	8E-09	1.3610
IFITM3	2E-12	2E-11	1.5042
IFNG	5E-06	7E-06	1.7346
IGDCC4	3E-04	4E-04	-1.8903
IGF2BP2	5E-12	4E-11	2.5703
IGF2BP3	1E-03	1E-03	2.1580
IGFBP3	2E-04	2E-04	1.3795
IGFBP5	3E-11	1E-10	-2.0841
IKZF2	7E-12	5E-11	-1.7199
IKZF3	2E-03	2E-03	1.2402
IL12RB1	4E-07	7E-07	1.8556
IL12RB2	2E-07	3E-07	2.9614
IL15RA	1E-09	3E-09	1.1279
IL17D	5E-03	6E-03	-1.5923
IL17RD	1E-10	5E-10	-1.7262

IL18BP	7E-08	1E-07	1.4508
IL18R1	1E-06	2E-06	1.2686
IL1A	6E-06	9E-06	1.7835
IL1RAP	4E-07	7E-07	1.2239
IL21R	2E-09	5E-09	2.0598
IL27RA	6E-07	1E-06	1.0748
IL2RA	3E-08	7E-08	1.9904
IL2RB	5E-08	1E-07	1.6464
IL2RG	2E-04	2E-04	1.0345
IL31RA	7E-06	1E-05	1.7409
IL34	3E-14	8E-13	-2.4454
IL7R	5E-09	1E-08	1.9868
INHBA	3E-17	4E-15	4.6517
INPP4B	1E-08	3E-08	1.1197
IQGAP2	1E-02	1E-02	-1.0984
IQGAP3	1E-07	2E-07	1.4878
IRF1	1E-06	2E-06	1.0292
IRF7	9E-15	3E-13	2.1023
IRS1	2E-10	9E-10	1.5505
IRX5	1E-06	2E-06	-1.2294
ISG15	1E-14	3E-13	3.1523
ISG20	6E-06	9E-06	1.3326
ISM1	2E-07	4E-07	-1.1934
ITGA11	1E-04	1E-04	1.2689
ITGA3	1E-15	7E-14	2.3667
ITGA5	4E-08	9E-08	1.4915
ITGA6	4E-12	4E-11	1.9056
ITGA7	5E-06	7E-06	-2.3047
ITGAV	2E-06	3E-06	1.0539
ITGB4	2E-09	5E-09	1.6204
ITGB6	3E-08	6E-08	2.1471
ITIH4	6E-04	8E-04	-1.1765
ITIH5	5E-09	1E-08	-1.5529
ITM2A	1E-10	5E-10	-1.7048
ITPR3	3E-10	1E-09	1.3314
IVD	2E-06	3E-06	-1.3657
JAK3	4E-05	5E-05	1.1147
JAKMIP1	1E-04	1E-04	1.3073
JAM2	8E-08	2E-07	-1.1055
JPH2	2E-03	2E-03	-2.5661
KALRN	8E-06	1E-05	-1.1833
KANK3	2E-06	4E-06	-1.3324

KAZALD1	1E-06	2E-06	-1.9150
KBTBD3	6E-07	1E-06	-1.2652
KCNAB1	1E-09	4E-09	-1.2569
KCNE3	5E-02	5E-02	-1.1958
KCNIP3	1E-07	3E-07	-1.1473
KIAA0408	4E-07	7E-07	-2.9569
KIAA1147	2E-06	3E-06	-1.2337
KIAA1199	5E-07	8E-07	1.0983
KIAA1211L	1E-09	5E-09	-1.8622
KIAA1244	2E-07	3E-07	-5.1199
KIAA1324	8E-03	9E-03	-5.3231
KIAA1377	5E-07	9E-07	-1.4834
KIAA1456	5E-12	4E-11	-2.3030
KIAA1524	3E-12	2E-11	1.7522
KIAA1549	1E-03	1E-03	-1.3815
KIAA1549L	2E-03	2E-03	1.4098
KIAA1609	3E-08	6E-08	1.2870
KIAA1644	2E-08	5E-08	1.8862
KIAA1737	1E-07	2E-07	-1.1880
KIF11	1E-06	2E-06	1.2639
KIF14	2E-10	9E-10	2.4867
KIF18A	4E-10	2E-09	2.0880
KIF18B	8E-08	2E-07	1.7619
KIF20A	9E-10	3E-09	2.1223
KIF20B	1E-08	3E-08	1.2186
KIF23	2E-09	6E-09	2.1905
KIF2C	5E-11	2E-10	2.2087
KIF4A	1E-09	3E-09	2.0621
KIF5C	2E-05	2E-05	-1.3314
KIRREL	1E-13	2E-12	1.3828
KIT	3E-06	4E-06	-1.0155
KL	2E-06	3E-06	-1.0380
KLB	8E-15	3E-13	-1.7343
KLF15	4E-12	3E-11	-4.3668
KLF7	1E-11	8E-11	1.3748
KLF8	1E-04	1E-04	-1.0826
KLF9	2E-06	4E-06	-1.1670
KLHDC1	2E-10	1E-09	-1.7138
KLHDC7B	1E-08	3E-08	1.8595
KLHL23	2E-08	5E-08	-1.1245
KLHL31	1E-04	2E-04	-2.8944
KLHL6	2E-05	3E-05	1.1751

KLRD1	6E-04	7E-04	1.7042
KMO	8E-06	1E-05	1.2612
KNSTRN	3E-11	1E-10	1.3361
KPNA2	1E-11	8E-11	1.5231
KRBA2	5E-08	1E-07	-1.5202
KREMEN2	4E-06	6E-06	2.1912
KRT13	5E-05	7E-05	-2.5451
KRT14	4E-04	4E-04	1.2777
KRT16	3E-04	4E-04	1.3482
KRT17	6E-08	1E-07	2.3896
KRT6B	1E-03	1E-03	1.0972
KY	4E-06	5E-06	-3.8672
KYNU	5E-12	4E-11	2.6405
L3MBTL4	3E-12	3E-11	-2.0656
LAG3	7E-10	3E-09	2.5579
LAIR1	2E-05	3E-05	1.2211
LAMA1	4E-09	1E-08	2.5753
LAMA2	2E-05	2E-05	-1.6341
LAMA3	4E-10	2E-09	2.8345
LAMB3	1E-05	2E-05	1.7756
LAMC2	4E-13	5E-12	4.2610
LAMP3	1E-07	2E-07	2.0763
LANCL3	3E-07	6E-07	-1.4278
LAP3	2E-09	5E-09	1.2357
LAPTM5	7E-06	1E-05	1.0528
LAX1	6E-03	7E-03	1.2323
LCA5	4E-08	9E-08	-1.1560
LCA5L	1E-04	2E-04	-1.3166
LCK	4E-05	6E-05	1.1749
LDB3	1E-05	1E-05	-4.9971
LDHD	3E-06	5E-06	-1.9932
LDOC1	1E-10	6E-10	-2.8873
LEPRE1	1E-13	2E-12	1.5504
LEPREL1	1E-03	1E-03	1.4506
LGI4	2E-09	6E-09	-1.7324
LIFR	5E-13	7E-12	-2.7499
LILRB1	5E-07	8E-07	1.7751
LILRB4	2E-06	3E-06	1.6439
LIMA1	1E-14	4E-13	1.4842
LIMCH1	8E-06	1E-05	-1.6588
LIMK1	4E-14	9E-13	1.2232
LIMS2	3E-10	1E-09	-1.6209

LINGO4	3E-10	1E-09	-3.6310
LIPG	5E-07	8E-07	1.5218
LIPH	2E-03	2E-03	-1.7115
LMF1	5E-10	2E-09	-1.0472
LMNB2	5E-09	1E-08	1.1743
LMOD1	4E-10	2E-09	-2.7899
LMTK3	4E-03	4E-03	1.0373
LNX1	1E-08	3E-08	-1.1882
LOC100129083	6E-08	1E-07	1.5329
LOC100129480	3E-05	4E-05	-1.2666
LONRF1	3E-16	2E-14	-1.7561
LONRF3	4E-08	9E-08	-2.1866
LOX	2E-06	3E-06	1.6614
LPCAT1	2E-15	8E-14	1.6538
LPIN1	2E-04	2E-04	-1.0743
LPL	4E-09	1E-08	-2.6367
LRFN4	2E-12	2E-11	1.6460
LRIG1	1E-06	2E-06	-1.4147
LRP3	2E-10	7E-10	-1.6638
LRP6	9E-09	2E-08	-1.2164
LRP8	3E-07	5E-07	1.1660
LRRC15	8E-11	4E-10	2.4878
LRRC4	3E-04	3E-04	-1.2984
LRRC6	3E-09	8E-09	-1.6108
LRRC8D	7E-10	2E-09	1.2903
LRRK2	2E-10	8E-10	-1.4998
LRRN1	2E-09	6E-09	-1.4268
LRRN4CL	2E-12	2E-11	-2.7185
LTBP1	6E-13	8E-12	1.9190
LTBP4	4E-13	6E-12	-2.0275
LTC4S	8E-10	3E-09	-1.2348
LTF	1E-05	1E-05	-3.4355
LUM	3E-08	7E-08	1.2184
LUZP1	6E-12	4E-11	1.0654
LY6E	9E-11	4E-10	1.8161
LY6K	7E-08	1E-07	2.7234
LYN	6E-08	1E-07	1.1670
LYPD1	1E-16	9E-15	3.5227
LYPD5	8E-04	1E-03	1.3504
LYRM9	3E-08	7E-08	-1.5677
LYVE1	3E-09	9E-09	-2.0493
MAB21L1	6E-09	2E-08	-2.1246

MAB21L2	1E-08	3E-08	-1.9719
MACROD1	7E-07	1E-06	-2.2225
MAGI1	2E-14	5E-13	-1.4811
MAGI2	3E-09	8E-09	-1.8433
MAGIX	3E-11	2E-10	-1.9062
MAL	6E-09	1E-08	-3.7294
MAMDC2	6E-14	1E-12	-2.8702
MAML3	6E-12	5E-11	-1.5999
MAMSTR	1E-09	3E-09	-1.6176
MAN1C1	3E-07	5E-07	-1.3277
MANSC1	1E-09	4E-09	-2.1468
MAOB	2E-14	6E-13	-3.8300
MAP10	2E-06	3E-06	-1.3468
MAP1LC3A	1E-12	1E-11	-1.8091
MAP2K6	2E-07	3E-07	-1.5660
MAP6	6E-08	1E-07	-1.5384
MARC1	7E-11	3E-10	-2.5105
MARC2	1E-09	3E-09	-2.1638
MARCKSL1	2E-10	9E-10	1.3468
MARVELD1	2E-11	1E-10	1.4090
MATN3	1E-09	5E-09	2.2530
MB	3E-04	4E-04	-3.5567
MB21D1	5E-15	2E-13	2.1473
MC1R	3E-11	2E-10	2.7132
MCF2L	6E-13	7E-12	-2.1142
MCM10	1E-09	4E-09	1.9513
MCM2	3E-10	1E-09	1.3487
MCM4	2E-08	4E-08	1.2965
MCOLN2	4E-04	5E-04	1.1554
MDFI	2E-09	7E-09	1.5085
ME3	2E-07	3E-07	-1.7803
MEF2C	2E-02	2E-02	-1.2644
MEFV	6E-07	1E-06	1.3091
MEIS1	2E-10	7E-10	-2.0808
MEIS2	3E-08	8E-08	-1.5790
MELK	2E-09	7E-09	2.3040
MET	5E-12	4E-11	1.6109
METTL21A	4E-11	2E-10	1.1231
METTL24	9E-13	1E-11	-2.0454
METTL25	1E-10	6E-10	-1.0473
METTL7A	2E-09	6E-09	-1.7984
MFAP2	2E-19	1E-16	3.0202

MFAP4	8E-13	9E-12	-2.7925
MFSD10	2E-13	3E-12	1.1477
MGAT4A	6E-03	7E-03	-1.0286
MGLL	3E-11	2E-10	-1.6523
MGP	2E-10	7E-10	-2.3927
MICAL2	5E-13	6E-12	2.0627
MIPOL1	2E-06	3E-06	-1.2532
MITF	9E-09	2E-08	-2.0876
MKI67	2E-07	4E-07	1.7403
MLANA	6E-08	1E-07	-2.2510
MLPH	2E-10	7E-10	-4.5182
MMP1	2E-21	3E-18	7.2122
MMP11	4E-16	3E-14	5.7765
MMP12	1E-14	4E-13	4.8500
MMP13	1E-18	5E-16	9.1572
MMP14	4E-12	3E-11	1.6262
MMP17	3E-08	7E-08	1.7375
MMP3	6E-10	2E-09	2.2588
MMP9	6E-13	7E-12	3.3374
MMRN1	6E-09	1E-08	-1.8780
MOB3B	2E-10	8E-10	1.3726
MOCOS	1E-07	3E-07	1.2650
MPC1	8E-09	2E-08	-1.4916
MPDZ	5E-05	6E-05	-1.2026
MPP7	7E-06	1E-05	-1.0407
MPZ	1E-13	2E-12	-3.4458
MROH8	4E-06	5E-06	-1.2632
MSC	6E-05	7E-05	1.2590
MSN	2E-14	5E-13	1.4739
MSRB1	2E-08	5E-08	1.0822
MSS51	3E-03	3E-03	-2.6630
MST1R	1E-05	2E-05	1.3935
MT2A	5E-09	1E-08	1.2877
MTAP	3E-05	3E-05	1.2510
MTBP	1E-10	5E-10	1.6793
MTFR2	1E-09	4E-09	1.7212
MTUS1	3E-07	5E-07	-1.2964
MTUS2	2E-10	8E-10	-2.6448
MX1	4E-11	2E-10	1.7592
MX2	1E-09	5E-09	1.8744
MYB	1E-05	2E-05	-1.1859
MYBL2	1E-08	3E-08	2.4087

MYCN	7E-04	8E-04	-2.3655
MYEF2	5E-08	1E-07	-1.6288
MYH10	6E-08	1E-07	1.0808
MYH11	2E-10	9E-10	-3.5839
MYH14	2E-10	8E-10	-2.5741
MYLIP	1E-11	8E-11	-1.2372
MYO10	6E-11	3E-10	1.4719
MYO1B	5E-15	2E-13	2.1692
MYO3B	1E-05	2E-05	2.0733
MYO5A	4E-12	3E-11	1.5677
MYO5C	1E-08	3E-08	-2.6412
MYO7A	1E-06	2E-06	1.1517
MYOM1	9E-06	1E-05	-4.2337
MYOT	3E-04	4E-04	-3.8292
N4BP3	8E-08	2E-07	-1.4130
NAALADL2	8E-10	3E-09	-3.6426
NAGS	2E-10	7E-10	1.9488
NBEA	5E-09	1E-08	-2.9474
NCALD	2E-02	2E-02	-1.7963
NCAM1	9E-04	1E-03	-1.2399
NCAM2	4E-08	9E-08	-1.5896
NCAPG	4E-10	1E-09	1.9757
NCAPH	7E-10	2E-09	1.9184
NCS1	2E-09	5E-09	1.2176
NDC80	3E-10	1E-09	2.0205
NDNF	1E-13	2E-12	-2.9335
NDRG1	7E-05	8E-05	1.1223
NDRG2	1E-07	2E-07	-2.4755
NEB	1E-03	1E-03	-3.2335
NEBL	5E-07	9E-07	-1.4777
NEFL	9E-09	2E-08	4.0864
NEGR1	1E-06	2E-06	-1.4401
NEIL1	5E-12	4E-11	-1.9210
NEK2	6E-07	1E-06	1.6460
NELL2	4E-10	1E-09	4.3975
NETO2	2E-07	3E-07	1.4086
NFIA	2E-18	6E-16	-1.3929
NFIB	5E-08	1E-07	-1.5196
NFIC	3E-11	1E-10	-1.2616
NFIX	3E-16	2E-14	-2.1032
NGF	9E-08	2E-07	2.0937
NID1	2E-09	6E-09	1.6695

NID2	1E-09	4E-09	1.5164
NIPAL4	2E-04	3E-04	1.2676
NKX3-1	6E-03	6E-03	-4.5334
NLGN3	1E-08	3E-08	-1.2011
NLGN4X	4E-04	5E-04	2.6495
NMI	5E-12	4E-11	1.4832
NNAT	1E-06	2E-06	-1.1618
NOSTRIN	2E-06	3E-06	-2.2794
NOXA1	8E-09	2E-08	-1.5864
NPDC1	6E-09	2E-08	-2.1702
NPHP1	3E-02	3E-02	-1.3079
NPNT	9E-04	1E-03	1.3626
NPR1	3E-08	6E-08	-1.6604
NPTXR	1E-09	4E-09	-2.2254
NR3C2	7E-13	9E-12	-3.3388
NR4A1	4E-06	6E-06	-2.0077
NR4A2	5E-07	9E-07	-1.8883
NR4A3	3E-02	3E-02	-1.1574
NREP	6E-10	2E-09	1.4048
NRG1	1E-06	2E-06	1.9538
NRG2	1E-17	2E-15	-3.4454
NRIP2	3E-08	7E-08	-1.4229
NRIP3	5E-09	1E-08	2.1459
NRP2	2E-08	4E-08	1.1800
NT5E	5E-08	1E-07	1.8600
NTN4	4E-09	1E-08	-1.2748
NTRK2	4E-07	7E-07	-1.6241
NTRK3	4E-14	8E-13	-3.0974
NUPR1	5E-08	1E-07	-1.2151
NUSAP1	4E-10	1E-09	1.4261
NXPH3	5E-14	9E-13	-2.3610
NYNRIN	2E-09	7E-09	-1.4853
OAS1	4E-08	8E-08	1.3884
OAS2	9E-13	1E-11	2.3530
OAS3	5E-14	1E-12	2.2166
OASL	6E-12	4E-11	2.6527
OBSCN	2E-04	2E-04	-3.6784
OCIAD2	1E-11	8E-11	1.9598
OCLN	2E-05	2E-05	-1.3721
ODC1	5E-05	7E-05	1.0974
OGFRL1	5E-10	2E-09	-1.1845
OGN	7E-11	3E-10	-2.4295

OIP5	5E-08	1E-07	1.4315
OLFML2B	1E-09	3E-09	1.4690
OLR1	3E-07	5E-07	2.3981
ORC1	3E-08	6E-08	1.7845
ORC6	2E-12	2E-11	1.8661
OSBPL1A	4E-05	6E-05	-1.0528
OSBPL3	5E-09	1E-08	1.2423
OSMR	6E-10	2E-09	1.1797
OSR1	1E-12	1E-11	-3.5451
OVGP1	3E-08	6E-08	-1.8885
P2RY6	6E-10	2E-09	1.8160
PABPC5	1E-05	2E-05	-1.2404
PAIP2B	8E-12	5E-11	-2.4008
PALM	3E-12	2E-11	-2.1516
PALMD	3E-09	9E-09	-1.5469
PAMR1	5E-08	1E-07	-1.4823
PANX1	2E-09	5E-09	1.1990
PAQR8	4E-04	5E-04	-1.1563
PARD3B	4E-14	8E-13	-1.6069
PARK2	3E-13	4E-12	-2.7978
PARM1	2E-05	3E-05	-1.8838
PARP12	1E-18	5E-16	2.1457
PARP14	9E-14	2E-12	1.7408
PARP9	1E-14	4E-13	1.5448
PATL2	4E-04	4E-04	1.3293
PAX9	1E-09	4E-09	-2.5467
PBX1	6E-16	4E-14	-2.5070
PCCA	1E-11	8E-11	-1.5220
PCDH17	4E-14	9E-13	2.1731
PCDH7	3E-07	5E-07	1.5161
PCDHA1	4E-09	1E-08	-2.3915
PCED1B	3E-09	8E-09	1.3495
PCYT1A	2E-07	4E-07	1.0334
PDCD1LG2	2E-08	5E-08	2.0530
PDE1A	8E-08	2E-07	-1.7879
PDE1C	2E-03	3E-03	-1.3672
PDE2A	8E-07	1E-06	-1.3005
PDE4D	4E-05	5E-05	-1.2862
PDE4DIP	2E-03	2E-03	-2.1790
PDE8B	4E-06	6E-06	-2.8629
PDE9A	4E-05	5E-05	-1.5500
PDGFD	2E-08	5E-08	-1.7705

PDK4	8E-09	2E-08	-3.1236
PDLIM3	4E-05	5E-05	-2.3086
PDP1	4E-10	1E-09	1.0348
PDPN	4E-14	8E-13	2.9754
PDZD4	2E-11	1E-10	-1.5595
PDZRN3	5E-08	1E-07	-1.5661
PEG3	5E-12	4E-11	-3.2862
PER2	8E-12	6E-11	-1.1230
PER3	1E-08	3E-08	-1.2732
PFKFB1	3E-06	5E-06	-3.5574
PFKM	4E-02	4E-02	-1.8935
PFN2	5E-08	1E-07	1.1351
PGAM2	1E-03	1E-03	-3.5234
PGAP3	1E-12	1E-11	-1.1141
PGD	2E-04	2E-04	-1.2215
PGF	9E-09	2E-08	1.8346
PGM1	2E-04	3E-04	-1.5584
PGM5	6E-10	2E-09	-2.8371
PGPEP1	3E-05	4E-05	-1.4002
PGR	3E-08	7E-08	-2.3417
PHC1	2E-09	5E-09	-1.2760
PHF17	1E-11	8E-11	-1.4021
PHGDH	6E-08	1E-07	-1.4121
PHKA1	2E-05	2E-05	-1.5694
PHLDB2	2E-12	2E-11	1.3585
PHYH	3E-06	5E-06	-2.6290
PHYHD1	3E-14	8E-13	-2.9439
PI3	3E-04	3E-04	1.1243
PID1	1E-05	2E-05	-1.0339
PIDD	2E-08	4E-08	1.0709
PIEZO2	4E-07	8E-07	1.2575
PIF1	5E-11	3E-10	1.9863
PIGZ	6E-10	2E-09	-1.2892
PIK3CD	9E-10	3E-09	1.2715
PIK3R1	7E-08	1E-07	-1.0301
PIP5KL1	5E-05	6E-05	1.4445
PITX1	1E-05	2E-05	-1.3817
PITX2	2E-09	7E-09	-2.6060
PKDCC	1E-09	3E-09	-3.2363
PKIA	7E-05	8E-05	-3.0108
PKIB	7E-07	1E-06	-1.8592
PKMYT1	3E-12	3E-11	2.1265

PKNOX2	5E-07	8E-07	-1.9634
PLA2G16	2E-04	2E-04	-1.5557
PLA2G7	1E-12	2E-11	3.0587
PLAC9	1E-14	3E-13	-2.6480
PLAGL1	2E-13	3E-12	-2.0050
PLAU	4E-15	1E-13	2.6756
PLCB4	1E-08	3E-08	-3.9793
PLCE1	2E-06	4E-06	-1.4402
PLEK2	7E-08	1E-07	2.3409
PLEKHA6	3E-09	9E-09	-1.9532
PLEKHA7	7E-08	1E-07	-1.5002
PLEKHB1	2E-06	3E-06	-3.5245
PLEKHG2	3E-11	1E-10	1.1897
PLEKHG6	3E-10	1E-09	-2.3902
PLIN4	1E-14	4E-13	-4.1475
PLK1	2E-10	1E-09	2.4105
PLK4	1E-07	2E-07	1.3527
PLLP	2E-13	3E-12	-2.3056
PLOD1	3E-14	8E-13	1.2510
PLOD2	9E-05	1E-04	1.5777
PLS1	8E-03	8E-03	-1.1552
PLSCR1	2E-12	2E-11	1.3858
PLXNA1	1E-12	1E-11	1.3644
PMEL	2E-07	4E-07	-4.2080
PMEPA1	1E-09	5E-09	1.5351
PMFBP1	2E-06	3E-06	1.4107
PML	6E-14	1E-12	1.5905
PNMAL2	1E-07	3E-07	-1.2880
PNPLA7	1E-11	8E-11	-3.1497
POC1A	3E-08	6E-08	1.2925
PODN	5E-10	2E-09	-2.3286
PODXL2	3E-08	6E-08	-2.4097
POLQ	5E-08	1E-07	1.4852
POLR3G	3E-05	3E-05	1.1187
POP1	2E-08	5E-08	1.2213
POPDC2	3E-03	3E-03	-3.0932
POSTN	4E-11	2E-10	3.2093
POU2F2	3E-06	5E-06	1.3353
PPAP2B	4E-12	4E-11	-1.8261
PPAPDC3	1E-02	1E-02	-2.9552
PPARG	2E-11	1E-10	-2.4589
PPARGC1A	2E-10	7E-10	-3.3979

PPFIA1	4E-05	6E-05	1.0349
PPIAL4B	2E-09	6E-09	-1.5942
PPIF	8E-05	1E-04	1.0112
PPIL6	2E-09	5E-09	-1.7540
PPL	2E-03	2E-03	-1.2879
PPM1H	1E-03	1E-03	-2.7344
PPM1L	3E-13	4E-12	-2.1250
PPP1R12B	7E-08	1E-07	-2.1011
PPP1R14C	2E-06	3E-06	1.6371
PPP1R3C	9E-10	3E-09	-3.7391
PPP1R3F	2E-06	4E-06	-1.4391
PPP4R4	2E-12	2E-11	3.9446
PRADC1	6E-10	2E-09	-1.2755
PRC1	2E-09	5E-09	1.5505
PRELP	7E-12	5E-11	-2.4845
PREX2	5E-08	1E-07	-1.2329
PRH1	3E-04	4E-04	-6.7574
PRKAR2B	5E-07	9E-07	-2.0328
PRKG2	6E-07	1E-06	-1.5709
PRLR	2E-10	9E-10	-2.7280
PRNP	2E-11	1E-10	1.4802
PROCR	2E-13	2E-12	2.0598
PROSAPIP1	4E-03	5E-03	-1.3680
PRPH2	1E-05	1E-05	-2.1037
PRR15L	1E-05	1E-05	-3.7972
PRRT3	5E-08	1E-07	-1.1325
PRSS27	2E-03	3E-03	-1.4930
PRSS53	2E-05	2E-05	1.4888
PRUNE2	2E-04	2E-04	-2.2852
PRX	1E-10	6E-10	-2.1847
PSMB2	8E-16	5E-14	1.1750
PSRC1	2E-08	5E-08	1.5850
PTCD3	1E-05	2E-05	-1.1781
PTCHD4	3E-04	3E-04	1.4423
PTGER3	2E-07	4E-07	-1.5432
PTGFR	1E-06	2E-06	-1.3508
PTGFRN	4E-10	1E-09	1.4212
PTGR1	2E-04	3E-04	-1.0740
PTGS2	6E-04	7E-04	1.2808
PTHLH	7E-12	5E-11	3.6661
PTK7	3E-11	2E-10	1.8532
PTN	3E-14	7E-13	-3.0452

PTPN7	1E-05	2E-05	1.2332
PTPRK	6E-13	7E-12	1.5640
PTPRN2	1E-04	1E-04	-2.6603
PTPRO	3E-04	3E-04	1.0247
PTPRZ1	4E-05	6E-05	1.8292
PTTG1	2E-06	3E-06	1.2706
PVRL3	7E-06	1E-05	-1.1627
PXDN	4E-12	3E-11	2.1442
PYCARD	7E-08	1E-07	1.1429
PYGM	5E-06	8E-06	-4.5823
PYGO1	2E-07	4E-07	-1.8998
PYHIN1	5E-04	6E-04	1.2010
QRICH2	3E-14	8E-13	-1.6790
R3HDM2	3E-03	3E-03	-1.8323
RAB31	2E-12	2E-11	1.5198
RAC2	4E-10	2E-09	1.5943
RACGAP1	4E-08	9E-08	1.2056
RAD51	2E-11	1E-10	1.8750
RAD51AP1	3E-10	1E-09	1.7875
RAG1	1E-07	2E-07	2.7419
RAI14	3E-06	5E-06	1.2673
RAI2	2E-11	1E-10	-1.8418
RALGPS1	4E-14	8E-13	-1.6307
RANBP17	9E-04	1E-03	-2.4612
RASGEF1A	2E-04	2E-04	2.3904
RASL11B	1E-06	2E-06	1.4967
RASL12	4E-10	2E-09	-1.8030
RAVER2	2E-09	6E-09	-1.3506
RBM20	3E-11	2E-10	-3.1698
RBMS3	5E-05	6E-05	-1.0382
RBP1	2E-07	3E-07	2.6170
RBP7	2E-11	1E-10	-2.0142
RCAN2	3E-07	5E-07	-2.3701
RDH16	1E-07	3E-07	1.2062
RECQL4	3E-11	1E-10	1.5955
REEP2	2E-09	5E-09	-2.0997
RELN	5E-09	1E-08	-2.1686
REPS2	6E-05	8E-05	-1.2791
RERG	3E-10	1E-09	-1.9641
RFC4	3E-11	2E-10	1.3002
RGAG4	4E-08	9E-08	-1.2757
RGMA	3E-08	7E-08	-1.4747

RGS11	2E-12	2E-11	-1.9407
RGS20	3E-07	5E-07	2.1793
RGS4	4E-09	1E-08	2.8814
RHOBTB3	9E-04	1E-03	-1.2390
RIBC1	3E-08	6E-08	-1.0790
RIMS3	1E-02	1E-02	1.1577
RIN1	1E-06	2E-06	1.6117
RIPK2	1E-10	5E-10	1.1782
RLTPR	4E-04	5E-04	1.0139
RMND5B	2E-09	5E-09	-1.1297
RNASE7	1E-04	2E-04	1.5426
RNASEH2A	9E-11	4E-10	1.2508
RNF125	5E-07	8E-07	-1.0510
RNF150	2E-10	8E-10	-2.6183
RNF157	1E-03	1E-03	-2.7621
RNF165	5E-09	1E-08	-1.5934
RNF180	3E-13	4E-12	-2.2403
RNF213	1E-12	1E-11	1.3867
ROBO3	6E-06	9E-06	-1.0193
ROR1	5E-13	7E-12	-2.1505
RPS6KA4	6E-10	2E-09	1.3836
RRAS2	2E-12	2E-11	1.2944
RRM2	6E-07	1E-06	1.7380
RSAD2	6E-12	4E-11	3.0833
RSPO3	7E-08	1E-07	-2.2981
RTEL1	9E-10	3E-09	1.7786
RTKN	1E-10	7E-10	1.1289
RTN2	1E-02	1E-02	-1.8483
RTN4R	4E-05	5E-05	1.1799
RTP4	2E-07	3E-07	1.7781
RUNDC3A	8E-05	1E-04	2.1904
RUNX1T1	4E-10	1E-09	-1.4931
RUNX3	7E-08	1E-07	1.2174
RUVBL1	1E-11	7E-11	1.0459
RWDD3	4E-04	4E-04	-1.0296
RYR1	1E-02	1E-02	-2.6637
RYR2	9E-04	1E-03	-2.7635
RYR3	8E-04	9E-04	-2.1226
S100A12	9E-03	9E-03	1.2998
SALL2	1E-11	9E-11	-1.4764
SAMD5	3E-05	4E-05	-2.1039
SAMD9	5E-09	1E-08	1.3604

SASH1	1E-09	4E-09	-1.1693
SATB1	9E-06	1E-05	-1.1242
SBSPON	5E-09	1E-08	-1.7409
SCARA3	1E-08	2E-08	-1.3044
SCG5	2E-08	5E-08	2.4280
SCIN	6E-09	2E-08	-3.8499
SCML2	1E-08	3E-08	-1.2306
SCNN1D	4E-05	6E-05	2.7258
SCO2	5E-13	7E-12	1.7012
SCRG1	2E-10	1E-09	-1.3757
SCUBE2	2E-04	3E-04	-1.1075
SDK2	4E-07	7E-07	1.7152
SDPR	2E-09	5E-09	-1.9445
SDS	2E-09	6E-09	1.8225
SEC14L2	9E-06	1E-05	1.1464
SECTM1	1E-04	1E-04	1.0024
SELENBP1	3E-12	2E-11	-3.3806
SEMA3C	3E-10	1E-09	1.3869
SEMA3E	2E-10	8E-10	-3.9527
SEMA3G	6E-08	1E-07	-1.5805
SEMA6C	6E-04	7E-04	-3.0881
SEMA7A	6E-09	2E-08	1.6573
SEPP1	1E-07	2E-07	-1.0909
SERINC2	1E-06	2E-06	1.2310
SERINC4	6E-05	8E-05	-1.0099
SERPINE1	4E-12	3E-11	2.5601
SERPINE2	2E-08	4E-08	2.2933
SERPINH1	3E-17	4E-15	2.0613
SERTAD4	5E-08	1E-07	-1.3303
SESN1	6E-07	9E-07	-1.2312
SETMAR	1E-12	1E-11	-1.1635
SFRP1	6E-05	8E-05	-1.1894
SFXN3	1E-10	5E-10	1.1777
SGCD	1E-04	1E-04	-1.3996
SGIP1	1E-10	7E-10	1.4783
SGK223	6E-06	9E-06	1.1206
SGOL1	3E-06	5E-06	1.1007
SGOL2	2E-09	6E-09	1.4734
SH2D2A	1E-12	1E-11	2.4671
SH3BGR	6E-05	8E-05	-1.8629
SH3BGRL2	3E-17	4E-15	-3.5993
SH3D21	1E-04	2E-04	1.2706

SHC1	8E-14	2E-12	1.2667
SHC2	4E-08	8E-08	-1.5247
SHC3	3E-06	5E-06	-1.1987
SHCBP1	8E-11	4E-10	1.9989
SHE	3E-07	6E-07	-1.1305
SHISA4	6E-04	7E-04	-2.7391
SHROOM3	2E-06	3E-06	-2.1932
SIGLEC10	1E-07	3E-07	1.4211
SIGLEC7	3E-06	5E-06	1.3785
SIM2	8E-07	1E-06	-2.7983
SIRPA	6E-13	7E-12	1.3269
SIRPB1	2E-06	3E-06	1.3027
SIX2	3E-02	3E-02	-1.7790
SKA1	2E-08	5E-08	2.0079
SKA3	4E-09	1E-08	1.7094
SLA2	7E-06	1E-05	1.4320
SLAIN1	3E-06	4E-06	-1.7345
SLAMF6	3E-04	4E-04	1.3075
SLAMF7	2E-06	4E-06	1.3857
SLAMF8	3E-09	8E-09	1.8982
SLC10A1	4E-12	3E-11	-1.4832
SLC12A2	3E-02	3E-02	-2.2571
SLC13A4	6E-08	1E-07	-1.4820
SLC14A1	1E-08	3E-08	-2.2284
SLC15A3	1E-11	8E-11	1.9986
SLC16A1	3E-11	1E-10	2.0843
SLC16A14	1E-12	1E-11	-2.0446
SLC16A6	2E-05	3E-05	-1.9478
SLC16A7	2E-09	6E-09	-1.3272
SLC19A3	2E-05	2E-05	-1.4274
SLC1A1	4E-09	1E-08	-1.4486
SLC20A1	5E-11	3E-10	1.3565
SLC24A3	7E-06	1E-05	-1.2686
SLC25A21	5E-14	1E-12	-2.8029
SLC25A23	3E-13	5E-12	-1.5896
SLC25A25	1E-06	2E-06	-1.1781
SLC25A27	4E-09	1E-08	-1.1985
SLC25A29	6E-11	3E-10	-1.0831
SLC25A4	5E-06	7E-06	-3.5381
SLC26A2	5E-07	8E-07	-2.4368
SLC26A6	1E-13	2E-12	1.4536
SLC27A5	3E-09	8E-09	-1.1566

SLC29A2	2E-05	3E-05	-1.9310
SLC2A1	3E-06	5E-06	1.5931
SLC2A10	1E-02	1E-02	-1.5912
SLC2A6	2E-10	9E-10	1.6359
SLC2A9	3E-10	1E-09	1.8102
SLC37A1	4E-09	1E-08	-1.4982
SLC38A5	6E-06	8E-06	1.9335
SLC38A7	9E-15	3E-13	1.0480
SLC39A6	2E-06	3E-06	1.0337
SLC3A2	3E-12	2E-11	1.2350
SLC43A1	1E-04	1E-04	-1.6918
SLC44A3	1E-04	2E-04	-1.5935
SLC44A5	3E-06	4E-06	1.2010
SLC4A3	4E-09	1E-08	1.4194
SLC7A11	5E-05	7E-05	1.1617
SLC7A2	3E-10	1E-09	-2.6330
SLC7A5	1E-06	2E-06	1.3367
SLC7A7	2E-06	3E-06	1.2835
SLC7A8	1E-09	4E-09	1.8450
SLC9A2	1E-03	1E-03	-2.2556
SLC9A4	1E-08	2E-08	-1.4570
SLCO5A1	9E-04	1E-03	-2.1963
SLIT3	3E-12	3E-11	-2.4329
SLMO1	1E-11	9E-11	1.8039
SMAD9	3E-07	6E-07	-2.7166
SMARCD3	1E-07	2E-07	-2.1516
SMIM1	2E-03	3E-03	-2.7837
SMIM3	3E-11	2E-10	1.4556
SMIM5	2E-07	4E-07	-1.6358
SMOC2	9E-05	1E-04	-1.3914
SMS	7E-11	3E-10	1.1343
SMTN	1E-06	2E-06	1.1078
SMTNL1	1E-05	2E-05	2.0937
SNAI2	7E-13	9E-12	2.0457
SNAPC1	2E-08	4E-08	1.0447
SNCA	7E-04	8E-04	-1.0847
SNED1	7E-12	5E-11	-1.7956
SNRPN	7E-07	1E-06	-1.5942
SNTA1	1E-04	2E-04	-1.9873
SNTB1	5E-04	6E-04	-1.1710
SNX10	7E-12	5E-11	1.9733
SOBP	7E-07	1E-06	-2.0910

SOCS1	8E-10	3E-09	1.6707
SOD3	6E-12	4E-11	-1.8293
SORBS1	7E-12	5E-11	-2.8558
SORBS2	9E-09	2E-08	-2.1970
SORCS2	4E-09	1E-08	1.8998
SOX17	1E-04	2E-04	-1.3411
SOX5	6E-13	7E-12	-1.8782
SP110	1E-16	1E-14	1.8708
SP140	1E-05	2E-05	1.4816
SP140L	2E-12	2E-11	1.2214
SPAG16	3E-09	8E-09	-1.6865
SPAG5	8E-10	3E-09	1.7371
SPARC	2E-09	5E-09	1.1470
SPATA18	4E-09	1E-08	-1.6325
SPATA22	1E-05	2E-05	1.8970
SPATA6	2E-14	6E-13	-2.2817
SPC24	2E-08	5E-08	1.6536
SPEF2	7E-10	3E-09	-1.7195
SPIN3	4E-07	8E-07	-1.1162
SPINK5	1E-02	1E-02	-1.8121
SPP1	3E-08	8E-08	3.1072
SPRY2	6E-11	3E-10	-1.3968
SPRY4	6E-13	7E-12	2.0914
SPTB	1E-02	1E-02	-1.5366
SQLE	2E-05	3E-05	1.4397
SSBP2	1E-10	5E-10	-1.2608
SSC5D	2E-07	4E-07	-1.4628
SSPN	1E-06	2E-06	-1.6705
ST3GAL4	9E-05	1E-04	-1.2009
ST6GALNAC3	9E-05	1E-04	-1.1547
STARD4	1E-08	3E-08	1.3903
STAT1	1E-16	9E-15	1.9645
STAT2	2E-14	5E-13	1.4312
STC2	5E-12	4E-11	2.3152
STIL	4E-10	1E-09	1.6679
STK39	5E-04	6E-04	-1.0624
STON2	4E-08	9E-08	1.4316
STRA6	1E-05	2E-05	2.0101
STX19	2E-06	3E-06	-1.1852
STX1A	2E-13	2E-12	1.7100
SUCNR1	1E-06	2E-06	1.5148
SULF1	7E-08	1E-07	2.3789

SULF2	5E-07	9E-07	1.5891
SULT1A1	1E-07	3E-07	-1.3250
SUSD4	2E-08	5E-08	-1.9210
SUSD5	5E-10	2E-09	-1.8505
SVEP1	2E-06	3E-06	-1.1822
SVIL	2E-05	2E-05	-1.6626
SVIP	2E-10	7E-10	-2.0881
SYBU	6E-09	2E-08	-1.9922
SYDE2	2E-08	5E-08	-1.6354
SYNGR1	1E-13	2E-12	-2.3198
SYNM	9E-05	1E-04	-2.8301
SYNPO2	5E-07	9E-07	-2.2801
SYP	2E-10	9E-10	-1.3538
SYT12	3E-08	7E-08	2.5736
SYT15	6E-13	8E-12	-2.2110
SYT8	3E-06	5E-06	-3.0348
SYTL4	3E-12	2E-11	-2.0804
TACC3	2E-10	1E-09	1.3117
TACR1	1E-10	5E-10	-2.0224
TANC2	1E-09	5E-09	1.3231
TBKBP1	1E-08	3E-08	-1.4676
TBX15	3E-04	4E-04	-2.9050
TC2N	1E-06	2E-06	-1.2539
TCEA3	1E-09	4E-09	-3.1634
TCF3	2E-11	1E-10	1.0001
TCOF1	5E-12	4E-11	1.0641
TCP11L2	8E-11	4E-10	-1.3987
TDO2	9E-11	4E-10	3.2901
TDRD6	8E-12	6E-11	2.3592
TENC1	1E-10	6E-10	-1.7152
TENM1	7E-08	1E-07	-1.1512
TENM2	5E-09	1E-08	2.4877
TENM3	2E-07	3E-07	1.7080
TESC	5E-04	6E-04	-2.7982
TET1	5E-07	8E-07	-1.9481
TEX9	1E-02	1E-02	-1.1772
TGFA	3E-06	4E-06	1.4643
TGFB1	1E-09	3E-09	1.5250
TGFBI	4E-14	9E-13	2.6650
TGFBR3	8E-16	5E-14	-2.4947
TGM2	7E-05	8E-05	1.8703
THBS2	7E-12	5E-11	1.9892

THBS4	8E-07	1E-06	-1.4904
THSD4	8E-05	1E-04	-1.0097
THY1	4E-11	2E-10	1.5069
TIGIT	1E-06	2E-06	1.5515
TK1	5E-09	1E-08	1.6865
TLE2	2E-15	1E-13	-3.2439
TLR2	1E-07	2E-07	1.3551
TMC7	6E-14	1E-12	2.5871
TMEM132A	2E-12	2E-11	2.2282
TMEM132B	2E-09	7E-09	-1.4759
TMEM143	8E-05	1E-04	-2.0007
TMEM150B	2E-05	2E-05	1.4843
TMEM150C	3E-12	2E-11	-2.2567
TMEM170B	2E-04	3E-04	-1.3793
TMEM178B	6E-07	1E-06	-2.7352
TMEM182	3E-02	3E-02	-2.5501
TMEM184B	4E-11	2E-10	1.0346
TMEM200A	3E-04	4E-04	1.2200
TMEM200C	2E-03	2E-03	-2.1209
TMEM220	1E-10	5E-10	-1.7760
TMEM229B	7E-07	1E-06	1.7220
TMEM232	3E-11	2E-10	-2.0749
TMEM38A	1E-04	2E-04	-3.6389
TMEM47	4E-07	8E-07	-1.5839
TMEM63A	1E-02	1E-02	-1.4158
TMEM86A	6E-05	7E-05	1.1536
TMEM98	3E-05	4E-05	-1.0126
TMOD1	5E-06	7E-06	-2.8227
TMPRSS11A	7E-06	1E-05	-2.6886
TMX4	7E-07	1E-06	-1.0687
TNC	2E-11	1E-10	2.4243
TNFRSF10B	9E-08	2E-07	1.0092
TNFRSF11A	4E-08	8E-08	-1.7213
TNFRSF12A	2E-13	3E-12	1.8833
TNFRSF19	3E-07	6E-07	-1.8281
TNFRSF4	6E-08	1E-07	1.8363
TNIP3	4E-08	8E-08	1.6924
TNS1	7E-11	3E-10	-1.9903
TNS4	1E-05	2E-05	1.4489
TONSL	1E-09	4E-09	1.4492
TOP1MT	1E-08	3E-08	1.2731
TOP2A	2E-07	4E-07	1.6644

TOR4A	1E-07	2E-07	1.3264
TOX	2E-05	3E-05	1.1088
TP53INP2	6E-06	9E-06	-1.1476
TP63	1E-04	2E-04	1.0936
TPBG	5E-15	2E-13	1.9072
TPCN1	4E-11	2E-10	-1.1412
TPM2	6E-03	6E-03	-2.2711
TPPP	2E-11	1E-10	-2.2536
TPPP3	7E-04	8E-04	-1.1535
TPX2	5E-10	2E-09	2.3143
TRAM2	2E-10	8E-10	1.4353
TRANK1	1E-08	3E-08	1.1027
TREM2	5E-11	2E-10	2.7307
TRIL	7E-10	3E-09	-1.8072
TRIM21	4E-14	8E-13	1.4719
TRIM22	2E-10	7E-10	1.3456
TRIP13	1E-10	6E-10	2.1777
TRPC1	5E-10	2E-09	-1.3164
TRPM2	3E-10	1E-09	2.0576
TRPS1	7E-06	1E-05	-1.0150
TSPAN10	3E-07	6E-07	2.4384
TSPAN12	2E-06	2E-06	-2.9842
TSPAN6	2E-05	2E-05	-1.1372
TTK	2E-07	4E-07	1.6238
TTLL7	3E-03	3E-03	-1.1548
TTPAL	5E-09	1E-08	1.2279
TTYH3	3E-14	8E-13	1.5553
TUB	1E-11	8E-11	-1.9433
TXLNB	9E-04	1E-03	-3.1809
TYMS	4E-07	7E-07	1.4161
UBASH3B	8E-09	2E-08	1.4104
UBE2C	1E-09	4E-09	2.1877
UBE2L6	2E-14	6E-13	1.8706
UBL3	1E-13	2E-12	-1.2389
UBXN10	3E-06	4E-06	-2.1081
UHRF1	9E-09	2E-08	1.8868
ULBP3	7E-06	9E-06	1.6296
UPK3B	1E-03	1E-03	-2.3392
USB1	2E-16	1E-14	1.7299
USHBP1	1E-04	1E-04	-1.2847
USP13	2E-04	2E-04	-2.1676
USP18	2E-09	7E-09	2.1585

VAV2	1E-14	3E-13	1.9812
VDR	7E-07	1E-06	1.1888
VEGFC	5E-10	2E-09	2.4122
VNN1	1E-02	1E-02	1.5912
VSIG10	3E-07	5E-07	-1.4243
VWA2	1E-04	2E-04	-1.0341
WARS	2E-12	2E-11	2.4943
WASF3	6E-07	1E-06	-1.1908
WBSCR17	3E-04	4E-04	-1.2657
WDHD1	1E-10	5E-10	1.5148
WDR54	4E-13	6E-12	1.9038
WDR66	2E-12	2E-11	3.1563
WDR76	6E-09	2E-08	1.3214
WDR78	2E-08	4E-08	-1.6103
WDR86	1E-05	2E-05	1.3170
WIPF3	6E-05	7E-05	-2.1884
WISP1	6E-15	2E-13	2.6691
WISP2	1E-08	3E-08	-2.4643
WNK4	8E-08	2E-07	-3.8407
WNT3	9E-05	1E-04	1.1739
WSCD1	5E-08	1E-07	-2.1870
XDH	2E-06	3E-06	1.8235
XPR1	1E-15	7E-14	1.3883
XRCC2	1E-08	3E-08	2.5422
YEATS2	4E-11	2E-10	1.0088
ZBED3	2E-09	6E-09	-1.5349
ZBP1	6E-06	9E-06	1.9032
ZBTB16	1E-12	1E-11	-4.0361
ZBTB20	7E-06	1E-05	-1.1549
ZBTB47	2E-06	3E-06	-1.4780
ZBTB7C	1E-11	8E-11	-2.2378
ZC2HC1B	3E-15	1E-13	-1.7046
ZC2HC1C	7E-10	2E-09	-1.3158
ZC3H12B	2E-08	5E-08	-1.3049
ZC3H6	8E-12	6E-11	-1.1738
ZCWPW2	6E-10	2E-09	-2.2795
ZDHHC2	6E-05	8E-05	-1.4929
ZFP14	1E-07	2E-07	-1.1574
ZFP28	1E-13	2E-12	-1.9440
ZFP92	9E-06	1E-05	1.2299
ZFPM2	5E-14	1E-12	2.7020
ZMAT1	3E-08	6E-08	-1.7511

ZNF132	1E-13	2E-12	-1.7325
ZNF135	4E-10	2E-09	-1.9087
ZNF208	8E-10	3E-09	-1.4739
ZNF214	1E-04	1E-04	-1.0235
ZNF229	1E-11	7E-11	-2.4086
ZNF254	5E-12	4E-11	-1.7911
ZNF273	4E-09	1E-08	-1.1670
ZNF281	2E-09	6E-09	1.0248
ZNF287	4E-12	3E-11	-1.7883
ZNF345	7E-11	3E-10	-1.3449
ZNF354C	3E-11	2E-10	-1.5306
ZNF358	3E-06	5E-06	-1.1232
ZNF385D	4E-07	6E-07	-1.3087
ZNF396	8E-06	1E-05	-1.6963
ZNF415	7E-07	1E-06	-2.1457
ZNF420	3E-13	4E-12	-1.6665
ZNF423	5E-07	9E-07	-1.5418
ZNF433	1E-11	7E-11	-1.5733
ZNF469	3E-11	2E-10	2.1719
ZNF471	7E-14	1E-12	-2.5556
ZNF483	8E-09	2E-08	-1.7968
ZNF491	2E-08	4E-08	-1.8868
ZNF493	4E-10	1E-09	-1.3109
ZNF502	8E-08	2E-07	-1.0845
ZNF514	5E-10	2E-09	-1.0411
ZNF528	8E-11	4E-10	-1.6371
ZNF546	4E-07	8E-07	-1.1871
ZNF554	5E-11	3E-10	-1.0203
ZNF566	4E-08	8E-08	-1.1226
ZNF568	2E-12	2E-11	-1.8452
ZNF569	6E-10	2E-09	-1.3671
ZNF570	5E-12	4E-11	-1.4095
ZNF571	2E-11	1E-10	-1.4209
ZNF573	8E-09	2E-08	-1.1920
ZNF577	8E-09	2E-08	-1.0306
ZNF582	2E-08	4E-08	-1.5391
ZNF583	3E-07	5E-07	-1.2354
ZNF610	1E-06	2E-06	-1.5350
ZNF615	2E-09	6E-09	-1.1602
ZNF626	8E-12	6E-11	-2.0782
ZNF667	2E-07	4E-07	-2.0439
ZNF677	1E-11	8E-11	-2.0079

ZNF681	2E-09	5E-09	-2.2754
ZNF682	1E-09	3E-09	-1.4651
ZNF704	1E-08	3E-08	-1.8813
ZNF717	2E-11	1E-10	-1.9132
ZNF727	8E-09	2E-08	-1.1308
ZNF737	6E-07	1E-06	-1.3488
ZNF774	7E-07	1E-06	-1.2915
ZNF790	1E-12	1E-11	-1.4588
ZNF829	7E-11	3E-10	-1.3225
ZNF844	2E-14	4E-13	-2.2152
ZNF853	1E-07	2E-07	-2.0599
ZNF880	3E-11	1E-10	-2.0850
ZNF91	4E-12	3E-11	-2.2196
ZNFX1	7E-12	5E-11	1.2058
ZSCAN16	1E-08	2E-08	-1.1185
ZSCAN18	6E-16	4E-14	-2.3327
ZWINT	1E-07	2E-07	1.4133

Supplemental Table S4: Genes found significantly differentially expressed both in OSCC-GB & TCGA-HNSCC studies

Gene symbol	OSCC-GB		TCGA-HNSCC	
	Corrected p-value	log2(fold-change)	FDR	log2(fold-change)
ABCA4	1.4E-09	2.810	2.7E-04	1.477
ABCA8	2.3E-11	-2.972	2.4E-45	-2.948
ABCC2	1.9E-04	1.731	2.6E-08	1.823
ABI3BP	7.0E-12	-2.637	1.3E-20	-2.034
ABL2	4.3E-10	1.222	4.3E-19	1.088
ABLIM2	1.6E-03	-1.301	1.9E-19	-1.564
ACADS	1.8E-04	-1.684	1.2E-29	-1.123
ACADSB	3.6E-10	-2.298	4.4E-41	-1.570
ACHE	5.0E-05	-2.947	9.7E-30	-2.416
ACOT7	2.9E-09	1.557	3.1E-19	1.181
ACOX2	2.6E-09	-1.763	4.6E-63	-2.437
ACP5	2.0E-08	1.446	4.5E-13	1.130
ACPP	7.4E-06	-1.834	8.3E-28	-1.953
ACSM3	2.7E-11	-2.590	8.8E-39	-2.086
ACSS3	7.2E-09	-2.777	3.6E-28	-2.036
ACYP2	6.8E-05	-1.189	6.6E-36	-1.263
ADA	4.1E-13	1.767	1.7E-19	1.612
ADAM12	4.2E-15	4.013	7.1E-27	3.495
ADAM19	3.5E-07	1.678	5.6E-12	1.583
ADAM33	1.5E-11	-2.073	6.1E-13	-1.242
ADAMTS12	1.4E-12	3.095	7.2E-26	3.343
ADAMTS15	7.4E-06	1.330	5.4E-05	1.190
ADAMTS2	8.5E-14	2.703	4.0E-24	2.958
ADAMTS6	8.1E-07	1.580	1.0E-29	2.935
ADAMTS7	5.7E-09	1.619	3.0E-25	2.282
ADAMTSL2	2.5E-08	1.149	4.1E-26	1.712
ADAMTSL3	2.1E-11	-2.849	1.5E-09	-1.472
ADHFE1	4.7E-09	-3.111	2.7E-48	-2.431
ADPRHL1	3.6E-04	-2.365	1.2E-65	-2.858
ADSSL1	8.1E-06	-3.392	1.4E-39	-2.167
AFAP1L2	2.4E-08	1.580	1.7E-12	1.212
AGFG2	4.8E-07	-2.323	5.7E-135	-2.399
AGRN	1.3E-11	1.819	1.8E-23	1.612
AGTRAP	1.9E-13	1.229	8.8E-16	1.097
AIM2	1.0E-07	1.802	3.5E-26	3.492
AJAP1	1.8E-04	2.563	1.3E-07	2.103
AJUBA	1.6E-06	1.510	2.3E-10	1.001

AKAP12	1.1E-09	-1.688	2.0E-07	-1.058
AKAP6	1.7E-03	-1.340	3.2E-12	-1.623
ALDH1A1	3.3E-08	-3.081	7.8E-04	-1.127
ALDH3A1	2.3E-05	-2.434	1.7E-05	-1.399
ALDH3B1	6.4E-06	-1.159	4.2E-29	-1.599
ALDH6A1	1.6E-11	-2.151	8.8E-36	-1.399
ALG1L	1.5E-06	1.986	8.1E-15	1.832
ALOX12	5.6E-07	-2.820	1.2E-26	-2.461
AMIGO1	4.3E-08	-1.875	2.1E-19	-1.423
AMIGO2	6.4E-09	2.228	3.3E-18	2.314
AMOT	1.9E-09	-3.393	2.0E-34	-2.683
AMY2B	1.3E-08	-1.045	1.7E-28	-2.340
ANG	8.6E-11	-3.271	1.4E-13	-1.071
ANGPT2	6.7E-09	1.567	1.4E-15	1.447
ANGPTL1	2.0E-12	-3.206	2.2E-69	-3.653
ANGPTL6	8.7E-11	1.273	6.4E-15	1.152
ANGPTL7	1.2E-06	-2.357	4.9E-23	-3.336
ANK1	3.4E-02	-3.505	5.8E-27	-2.349
ANK2	5.3E-10	-2.887	8.7E-52	-2.722
ANKRD23	1.1E-03	-2.803	6.2E-48	-2.876
ANKRD35	1.2E-07	-1.452	2.5E-20	-1.885
ANLN	2.9E-07	2.057	7.8E-23	1.517
ANO5	3.5E-05	-3.724	1.9E-35	-3.443
AOC3	4.5E-10	-2.453	4.5E-22	-1.613
AOX1	1.0E-09	-2.724	7.0E-43	-2.415
APBA2	2.7E-11	2.443	7.1E-32	2.865
APBB1	6.9E-07	-1.440	2.3E-25	-1.445
APLN	2.0E-09	2.226	1.3E-13	1.921
APOBEC3G	1.2E-05	1.256	3.0E-08	1.154
APOD	9.9E-12	-3.259	5.0E-37	-2.685
APOL1	6.1E-12	2.581	1.2E-12	1.816
AQP1	7.2E-07	-1.103	3.2E-21	-1.331
AR	1.9E-11	-2.955	2.0E-17	-1.948
ARHGAP11A	7.9E-08	1.406	2.2E-24	1.321
ARHGAP20	1.7E-14	-2.110	2.3E-24	-1.794
ARHGAP6	3.7E-10	-1.970	3.9E-31	-1.566
ARHGEF38	3.9E-05	-5.114	2.4E-23	-2.096
ARPC1B	2.0E-13	1.608	2.0E-23	1.295
ARSJ	4.7E-08	1.671	3.6E-11	1.405
ASAP3	1.2E-10	-1.566	4.1E-27	-1.237
ASB16	6.5E-03	-1.497	1.6E-47	-2.448
ASCL2	9.6E-09	1.738	8.8E-06	1.087

ASPM	1.3E-06	1.439	1.7E-18	1.341
ATAD2	9.0E-08	1.434	1.3E-31	1.630
ATOH8	2.3E-07	-2.506	6.4E-18	-1.771
ATP1B3	2.5E-07	1.108	6.9E-16	1.127
ATP6V0E2	7.9E-08	-2.029	1.0E-12	-1.274
ATP8A1	2.7E-05	-1.562	3.8E-29	-1.729
AUNIP	3.7E-11	2.147	3.4E-32	1.801
AURKA	2.0E-12	2.051	3.2E-34	1.739
B3GNT4	6.5E-06	1.523	4.5E-18	1.920
B4GALNT1	3.7E-05	1.344	5.6E-18	2.426
BARX2	8.6E-08	-2.164	2.5E-32	-2.256
BASP1	8.1E-10	1.445	2.8E-11	1.545
BATF2	2.7E-09	3.309	9.8E-08	1.805
BCAS1	1.6E-06	-3.042	7.2E-27	-2.460
BCAS4	4.8E-06	1.114	6.3E-18	1.734
BCAT1	6.0E-08	1.586	2.6E-10	1.758
BCKDHB	1.8E-10	-1.426	2.0E-22	-1.068
BEND6	1.7E-09	1.901	6.8E-18	1.830
BEND7	4.9E-07	-2.544	2.0E-17	-1.667
BEX4	8.5E-14	-2.354	5.6E-20	-1.566
BFSP1	2.8E-05	1.217	2.3E-14	1.622
BGN	1.6E-10	1.372	1.9E-11	1.618
BHMT2	1.2E-08	-2.306	8.1E-33	-1.928
BICD1	8.0E-09	1.058	3.0E-28	1.571
BIRC5	1.6E-08	2.035	2.4E-32	1.767
BMP1	3.1E-11	1.887	4.2E-32	1.640
BMP8A	1.1E-09	1.981	6.1E-27	3.039
BMX	3.7E-06	-1.296	9.6E-13	-1.366
BNC1	6.1E-06	1.922	8.1E-11	1.366
BOC	7.2E-13	-1.968	7.5E-35	-1.855
BOP1	2.7E-07	1.203	4.9E-14	1.002
BORA	1.6E-10	1.288	1.4E-15	1.257
BRCA1	8.6E-11	1.339	2.2E-18	1.136
BRCA2	4.5E-08	1.525	5.2E-18	1.310
BRIP1	1.3E-06	1.371	1.5E-13	1.120
BSPRY	4.0E-03	-1.109	8.8E-20	-1.720
BST2	3.2E-13	2.590	8.0E-22	2.488
BUB1	7.2E-09	2.001	3.1E-31	1.525
C10orf55	6.1E-11	1.776	1.3E-17	1.845
C12orf75	1.4E-11	2.399	1.3E-13	1.524
C15orf62	2.0E-07	-1.107	7.7E-46	-2.480
C16orf74	5.8E-08	1.794	1.7E-19	1.753

C17orf53	2.3E-08	1.399	2.6E-16	1.285
C18orf54	1.2E-10	1.295	2.5E-29	1.590
C1orf115	3.1E-09	-2.562	5.8E-22	-1.535
C1QTNF6	5.0E-16	3.043	4.8E-32	2.722
C1QTNF7	8.0E-11	-1.405	3.9E-67	-3.292
C2CD4B	8.1E-06	-2.469	1.2E-17	-1.733
C2orf88	3.4E-09	-2.042	6.4E-83	-2.545
C3orf18	3.7E-05	-2.163	6.3E-23	-1.318
C4BPA	2.5E-09	-2.340	4.4E-02	-1.045
C4orf19	7.5E-04	-3.026	5.0E-18	-2.225
C4orf48	9.1E-13	2.119	2.2E-16	1.843
C6orf141	3.2E-08	2.248	1.1E-16	2.436
CA13	1.5E-09	-1.813	5.4E-27	-1.551
CA2	3.5E-04	1.627	2.8E-05	1.203
CAB39L	2.4E-15	-2.395	1.7E-97	-2.042
CACNA1D	2.0E-07	-2.231	7.5E-08	-1.200
CACNA2D1	1.0E-07	-2.539	4.0E-09	-1.254
CACNA2D2	4.2E-06	-1.883	3.1E-12	-1.063
CACNB1	4.6E-03	-2.106	8.7E-26	-2.023
CADPS2	2.3E-03	-1.584	2.3E-11	-1.077
CAMK4	1.6E-05	1.418	2.0E-09	1.210
CAND2	4.3E-07	-3.918	1.4E-49	-2.898
CAP2	1.2E-02	-2.297	2.9E-41	-2.322
CAPN14	1.7E-05	-2.434	3.2E-57	-4.058
CAPN3	2.7E-05	-2.269	5.9E-70	-3.525
CAPN5	2.4E-07	-1.695	7.1E-75	-2.612
CAPS	2.2E-06	-1.273	3.8E-51	-2.614
CARD11	8.8E-07	1.918	3.3E-07	1.304
CARNS1	3.2E-04	-3.231	1.3E-71	-3.058
CAV1	2.5E-06	1.259	1.7E-13	1.819
CBX7	7.8E-10	-1.850	2.4E-16	-1.155
CCDC110	2.2E-07	-2.494	1.6E-14	-1.506
CCL28	2.2E-03	-4.161	3.3E-165	-4.256
CCL5	9.8E-08	1.657	7.4E-11	1.842
CCM2	7.0E-14	1.231	2.9E-18	1.097
CCNB2	3.1E-09	1.958	5.0E-27	1.524
CCNE1	5.8E-11	1.580	1.5E-17	1.536
CCNF	2.5E-10	1.679	1.0E-19	1.242
CCRL2	1.3E-06	1.054	1.5E-09	1.080
CD207	8.7E-04	-1.533	7.6E-07	-1.264
CD274	1.6E-07	2.056	4.7E-06	1.260
CD276	1.3E-14	1.799	5.6E-37	1.812

CD300LF	1.8E-06	1.354	7.7E-13	1.567
CD36	6.4E-05	-1.382	1.1E-05	-1.128
CD38	1.1E-04	1.460	1.1E-05	1.117
CD72	3.2E-06	1.645	2.9E-07	1.223
CD80	1.7E-11	2.929	8.2E-23	2.372
CDC20	2.7E-09	2.475	4.3E-27	1.610
CDC25B	4.1E-13	2.027	8.0E-21	1.386
CDC45	1.7E-10	1.986	1.6E-30	1.848
CDC6	6.1E-11	2.312	1.8E-35	1.820
CDC7	4.2E-09	1.302	1.7E-14	1.136
CDCA2	5.0E-08	1.771	1.6E-16	1.342
CDCA3	9.1E-09	1.964	6.3E-30	1.794
CDCA4	1.5E-08	1.455	1.5E-22	1.164
CDCA5	2.4E-10	2.466	3.6E-43	1.957
CDCA7	2.1E-05	1.212	5.8E-09	1.057
CDCA8	1.4E-08	1.792	1.1E-26	1.521
CDH11	5.0E-08	1.598	4.5E-13	2.050
CDH24	4.7E-08	1.335	2.1E-32	1.724
CDH3	9.4E-08	2.733	6.2E-14	1.415
CDK1	1.0E-08	1.681	2.5E-23	1.442
CDK6	1.1E-09	1.426	8.4E-11	1.291
CDKN3	1.2E-11	1.951	7.4E-25	1.633
CDT1	5.9E-07	1.416	6.9E-20	1.408
CEACAM1	1.4E-06	-1.991	9.0E-42	-2.512
CEACAM5	1.1E-02	-1.129	5.8E-16	-2.394
CELSR3	4.6E-09	2.153	4.0E-18	2.448
CENPF	1.6E-07	1.542	9.3E-25	1.583
CENPI	3.0E-10	1.975	3.5E-32	1.767
CENPK	2.2E-07	1.609	8.8E-13	1.248
CENPL	1.1E-10	1.140	1.7E-27	1.213
CEP55	2.2E-10	2.407	4.7E-37	1.894
CERCAM	1.1E-10	1.336	3.0E-16	1.685
CFD	4.7E-12	-3.052	7.9E-74	-2.611
CFL2	1.7E-02	-1.975	2.9E-42	-1.949
CGNL1	4.7E-12	-3.349	2.2E-63	-2.983
CHADL	1.1E-12	-2.191	5.3E-12	-1.091
CHEK1	8.4E-10	1.725	2.1E-25	1.256
CHN1	1.7E-11	1.998	2.4E-20	1.721
CHPT1	6.1E-09	-2.435	6.9E-21	-1.612
CHRNA10	4.4E-04	-3.179	8.3E-57	-2.333
CHST1	8.8E-07	1.827	1.2E-05	1.167
CHST11	1.4E-12	1.922	2.9E-28	2.028

CHST2	1.8E-07	2.042	1.9E-19	2.549
CHTF18	3.3E-08	1.257	6.2E-25	1.360
CIDEC	7.2E-09	-1.927	3.7E-77	-5.709
CKAP2L	5.0E-08	2.060	1.4E-24	1.492
CKMT2	2.2E-05	-4.227	1.4E-47	-3.942
CKS2	1.0E-09	1.439	6.6E-18	1.463
CLCN4	3.5E-06	-2.891	1.5E-29	-2.096
CLDN11	6.6E-10	-2.504	3.7E-53	-2.658
CLDN14	2.2E-06	2.005	1.5E-16	2.851
CLDN5	5.4E-06	-1.679	9.7E-09	-1.001
CLDN7	1.8E-03	-1.869	9.9E-10	-1.200
CLEC3B	8.0E-14	-3.316	1.9E-164	-3.636
CLEC7A	4.7E-07	1.170	1.2E-10	1.477
CLIC5	9.0E-04	-2.945	8.2E-57	-3.211
CLIC6	2.2E-07	-2.105	4.3E-12	-1.674
CLYBL	8.7E-11	-1.721	1.2E-37	-1.579
CMA1	5.6E-07	-1.370	1.3E-16	-2.469
CMPK2	2.7E-10	2.502	5.3E-13	1.878
CMYA5	2.7E-04	-3.215	4.5E-32	-3.343
CNN1	5.3E-05	-2.458	1.8E-13	-1.532
COBL	1.7E-09	-2.647	8.4E-41	-2.711
COL10A1	3.3E-08	2.959	8.0E-32	5.571
COL12A1	2.1E-13	2.079	5.7E-26	2.884
COL13A1	2.7E-08	1.641	2.5E-32	2.323
COL14A1	6.7E-08	-1.296	2.5E-09	-1.216
COL16A1	1.9E-10	1.610	1.1E-16	1.657
COL17A1	2.8E-04	1.467	3.2E-09	1.596
COL1A1	5.3E-11	2.176	2.9E-22	3.433
COL22A1	7.8E-07	4.143	4.9E-16	3.796
COL27A1	1.6E-10	1.644	1.7E-27	2.075
COL3A1	1.3E-10	1.705	8.2E-18	2.939
COL4A1	2.1E-15	2.548	2.4E-33	2.524
COL4A2	6.3E-14	2.085	4.5E-27	2.207
COL4A5	1.0E-11	2.216	1.5E-25	2.121
COL4A6	1.2E-14	3.422	1.5E-17	2.878
COL5A1	4.6E-11	2.558	3.9E-24	3.089
COL5A2	4.1E-13	2.535	6.8E-25	3.052
COL5A3	8.3E-07	1.783	3.1E-19	2.136
COL6A1	4.8E-08	1.355	2.3E-15	2.018
COL6A3	1.8E-09	1.534	4.3E-16	2.469
COL7A1	2.5E-07	1.692	6.7E-28	2.263
COL8A1	1.9E-09	2.141	5.4E-14	2.321

COQ10A	6.8E-04	-2.663	4.6E-71	-2.020
COTL1	5.3E-10	1.321	5.0E-13	1.032
COX7A1	2.7E-07	-3.460	4.9E-97	-3.582
CP	1.4E-06	-2.530	6.4E-03	-1.023
CPEB3	2.7E-10	-1.615	2.9E-62	-1.767
CPXM1	1.4E-10	2.431	8.1E-05	1.018
CRAT	2.8E-08	-2.311	5.6E-41	-2.195
CRY2	1.0E-09	-1.347	1.2E-57	-1.317
CRYAB	2.2E-07	-2.202	2.8E-24	-2.205
CRYM	1.5E-05	-3.677	2.0E-32	-2.871
CSPG4	3.4E-07	1.552	6.3E-11	1.911
CTHRC1	2.6E-11	2.600	4.7E-20	2.644
CTSC	2.3E-11	1.556	4.1E-16	1.398
CTTNBP2	1.5E-15	-2.837	5.2E-22	-2.252
CX3CR1	6.6E-05	-1.020	5.1E-23	-1.883
CXCL10	3.9E-10	3.458	1.5E-10	2.848
CXCL11	7.2E-10	4.464	1.8E-11	3.375
CXCL12	3.9E-08	-1.543	6.3E-16	-1.661
CXCL9	1.1E-09	3.019	9.9E-09	2.266
CXCR2	3.7E-07	-1.821	2.0E-32	-2.492
CYP27B1	2.3E-11	3.596	3.7E-19	2.536
CYP2J2	2.9E-10	-2.155	8.3E-22	-1.564
CYP3A5	8.9E-05	-1.460	5.1E-47	-2.918
CYP4X1	1.4E-09	-2.883	6.1E-09	-1.576
DAGLA	1.1E-07	-1.721	4.1E-21	-1.591
DBF4B	5.2E-09	1.218	1.0E-24	1.319
DBNDD1	2.9E-09	-2.005	1.8E-10	-1.214
DCBLD1	4.3E-15	2.356	5.2E-24	2.018
DCBLD2	8.8E-06	1.395	2.3E-16	1.544
DCLK1	9.1E-09	-1.892	2.0E-07	-1.239
DDAH1	1.7E-06	-1.519	8.3E-15	-1.233
DDO	1.7E-03	-2.063	2.8E-14	-1.489
DDX58	2.9E-09	1.181	2.4E-13	1.635
DDX60	4.3E-12	2.263	1.2E-13	1.696
DDX60L	1.5E-11	1.981	1.2E-17	1.639
DEPDC1	5.8E-08	1.736	2.9E-20	1.295
DEPDC1B	1.8E-09	2.029	6.5E-26	1.680
DEPTOR	1.4E-11	-3.468	3.1E-61	-2.753
DGCR6	1.9E-04	-2.396	1.7E-18	-1.278
DIAPH3	1.1E-06	1.558	1.6E-15	1.156
DLEU7	2.3E-09	1.550	5.3E-18	1.671
DLG2	3.9E-13	-2.642	6.5E-90	-3.013

DLGAP3	3.1E-03	2.929	1.4E-13	2.517
DLGAP5	2.3E-08	2.163	1.5E-29	1.646
DLX1	7.3E-06	1.541	1.8E-15	2.359
DMD	2.9E-04	-1.860	7.2E-26	-1.959
DNA2	8.7E-09	1.116	8.5E-21	1.255
DNAH17	5.8E-10	3.036	5.8E-19	3.198
DNALI1	4.9E-06	-1.700	4.8E-51	-2.602
DNER	1.4E-02	1.735	2.3E-11	-1.810
DNMT1	4.1E-10	1.180	2.7E-15	1.011
DNMT3B	7.8E-11	2.473	2.2E-31	2.473
DOK5	9.6E-03	-1.737	2.6E-22	-1.918
DOK7	1.9E-03	-2.111	1.9E-07	-1.255
DPF3	2.5E-02	-2.134	1.0E-36	-2.251
DPT	4.5E-13	-3.210	5.4E-27	-2.259
DTL	4.1E-10	1.845	1.3E-33	1.872
DTNA	1.3E-06	-3.424	1.9E-28	-2.400
DUSP1	1.6E-06	-1.083	7.4E-11	-1.128
DYRK1B	1.6E-08	-2.188	2.2E-26	-1.411
EBF2	3.4E-05	-1.444	1.9E-08	-1.149
ECHDC3	2.4E-07	-1.786	6.1E-10	-1.420
ECT2	1.0E-07	1.240	1.1E-29	1.706
EDIL3	6.4E-08	1.483	3.4E-07	1.378
EDNRB	1.5E-08	-1.333	1.6E-19	-1.381
EFCAB6	6.7E-13	-2.372	2.0E-16	-1.443
EFHD1	2.6E-06	-1.777	7.6E-13	-1.412
EFNB1	7.3E-10	1.611	7.6E-20	1.269
EGFL6	7.8E-09	2.187	2.0E-07	1.196
EGR1	2.9E-05	-1.536	4.7E-13	-1.257
EHD3	3.5E-03	-1.008	2.2E-25	-1.364
EIF2AK2	1.8E-12	1.672	2.0E-28	1.219
EIF5A2	7.0E-10	1.798	1.3E-34	2.043
ELF3	2.5E-02	-1.258	4.9E-10	-1.349
ELF4	4.4E-09	1.190	1.3E-20	1.172
EMCN	9.8E-07	-1.171	1.5E-32	-1.648
EME1	4.8E-10	1.827	4.8E-39	2.038
EMP1	2.4E-06	-1.911	2.3E-84	-2.584
ENDOU	1.7E-04	-2.279	2.7E-36	-3.340
ENO3	3.3E-04	-3.918	9.8E-70	-4.355
ENPP4	1.5E-05	-2.163	5.0E-44	-2.453
EOMES	2.3E-05	1.416	1.5E-07	1.437
EPB41L4A	5.9E-12	-1.639	1.5E-26	-1.687
EPDR1	2.1E-03	-1.880	1.4E-18	-1.748

EPHB2	5.2E-09	2.068	5.7E-20	2.203
EPHB6	4.9E-06	-1.286	1.3E-07	-1.108
EPHX2	8.1E-11	-1.823	1.4E-26	-1.668
EPSTI1	6.9E-12	2.617	3.0E-15	2.022
ERCC6L	4.2E-09	1.517	4.2E-16	1.234
ERVMER34-1	6.1E-05	1.227	4.5E-12	1.554
ESCO2	3.3E-06	1.406	2.6E-13	1.257
ETNK2	3.0E-08	-3.389	9.6E-08	-1.005
ETV1	1.8E-02	-2.524	1.4E-14	-1.478
ETV7	4.7E-07	1.814	6.7E-06	1.041
EVA1A	4.1E-09	2.893	4.7E-10	1.796
EYA2	1.1E-09	-2.186	1.8E-12	-1.793
F2RL2	9.5E-08	1.858	1.3E-17	2.410
FABP3	4.0E-03	-3.241	2.9E-48	-3.010
FADD	4.6E-07	1.449	5.6E-15	1.822
FAIM2	1.5E-10	-2.316	4.6E-19	-1.648
FAM107A	4.3E-12	-2.949	4.0E-123	-3.683
FAM110B	1.1E-07	-1.989	3.5E-11	-1.156
FAM110D	3.7E-06	-1.115	2.2E-28	-1.541
FAM111B	2.6E-07	1.720	8.3E-11	1.218
FAM13C	1.8E-07	-1.554	2.1E-25	-1.891
FAM174B	2.9E-09	-2.499	1.2E-36	-1.643
FAM184A	6.9E-10	-2.605	4.6E-07	-1.112
FAM189A2	1.3E-10	-2.739	3.3E-32	-2.451
FAM47E	4.1E-04	-2.382	1.9E-28	-1.919
FAM72A	2.8E-02	1.219	8.5E-20	1.507
FAM89A	7.7E-08	1.505	1.3E-09	1.091
FANCA	1.7E-08	1.217	4.0E-23	1.393
FANCI	4.2E-09	1.577	5.7E-26	1.384
FAP	4.6E-11	2.567	8.0E-19	2.608
FBLIM1	2.5E-10	1.758	7.5E-10	1.013
FBN2	1.7E-08	2.717	1.2E-20	3.974
FBXO2	1.5E-03	1.121	2.6E-05	1.122
FBXO39	1.4E-12	2.434	9.1E-11	1.916
FCER1A	1.0E-09	-2.042	2.8E-20	-2.042
FCER1G	1.7E-06	1.417	5.4E-08	1.079
FCGBP	4.1E-04	-2.937	4.1E-07	-1.306
FCGR3A	1.8E-09	2.339	9.5E-11	1.670
FEN1	4.6E-11	1.494	5.8E-28	1.299
FERMT1	6.9E-06	1.598	2.9E-09	1.093
FEZ1	1.1E-12	2.216	2.0E-16	2.133
FGF18	2.2E-07	-1.727	6.7E-20	-1.822

FHIT	4.3E-11	-2.141	5.4E-25	-1.631
FHL1	2.0E-05	-2.790	4.8E-34	-2.896
FHL5	1.6E-08	-2.549	1.3E-13	-1.498
FILIP1	3.1E-03	-2.267	3.3E-23	-2.016
FITM1	8.6E-04	-4.352	7.6E-76	-4.110
FJX1	3.7E-10	1.978	4.6E-11	1.192
FLNC	2.8E-02	-2.685	3.8E-15	-2.174
FLRT2	8.7E-07	1.687	3.9E-09	1.606
FLRT3	9.3E-10	3.085	7.4E-05	1.439
FMNL3	1.7E-11	1.321	1.9E-23	1.318
FMO2	1.6E-12	-3.701	2.1E-39	-3.387
FMO5	5.1E-05	-2.525	2.1E-37	-1.747
FN1	5.0E-09	2.074	7.1E-17	3.145
FNDC1	5.1E-05	2.008	9.3E-08	1.850
FNDC4	4.5E-07	-1.633	8.4E-35	-1.750
FNDC5	5.1E-07	-4.732	9.5E-117	-4.911
FOS	5.6E-04	-1.165	7.3E-10	-1.211
FOSB	1.7E-06	-1.949	3.5E-12	-1.662
FOXC2	1.5E-04	1.291	5.2E-10	2.039
FOXM1	1.1E-08	2.083	4.6E-33	1.983
FOXO4	2.0E-10	-1.165	5.8E-29	-1.121
FPR3	1.2E-07	1.634	4.2E-08	1.211
FRMD3	2.1E-05	-2.611	1.5E-36	-1.921
FRZB	1.0E-05	-2.432	5.5E-66	-3.022
FSCN1	2.4E-10	2.186	4.3E-29	1.721
FST	3.7E-10	2.777	1.9E-21	2.584
FSTL3	2.1E-10	2.037	4.4E-18	2.202
FXYD2	3.9E-10	-2.806	1.4E-14	-2.587
FXYD5	8.6E-12	1.709	4.5E-15	1.496
FZD4	1.3E-06	-1.707	7.4E-26	-1.321
FZD6	2.6E-08	1.402	5.3E-20	1.196
GALNS	4.1E-10	1.079	1.1E-14	1.009
GALNT12	6.4E-10	-3.511	9.2E-27	-1.740
GALNT18	4.0E-11	1.440	2.0E-17	1.270
GALNT5	1.7E-04	-2.668	3.9E-10	-1.503
GAMT	1.6E-07	-2.853	1.4E-43	-2.086
GAS2	3.3E-07	-2.515	2.1E-09	-1.520
GAS7	5.9E-09	-1.439	2.8E-25	-1.641
GATM	7.7E-14	-2.919	4.0E-18	-1.600
GBP1	2.3E-11	2.264	8.4E-08	1.339
GCNT3	4.9E-04	-3.580	2.8E-29	-2.617
GDPD2	5.1E-06	2.817	2.3E-10	2.768

GFI1	1.2E-08	1.784	2.9E-18	2.105
GFRA1	1.1E-10	-3.021	2.5E-25	-2.302
GGT6	2.0E-02	-1.357	1.9E-26	-1.981
GINS4	1.0E-08	1.452	1.2E-14	1.434
GJA1	1.0E-04	1.066	3.1E-09	1.297
GJC1	2.6E-11	2.265	7.0E-40	3.048
GKAP1	5.1E-09	-2.405	1.8E-10	-1.156
GLIS1	9.1E-04	1.744	4.4E-17	2.548
GLRB	5.9E-06	-3.285	1.3E-24	-2.064
GMNN	1.0E-09	1.328	4.7E-19	1.324
GMPR	4.7E-05	-2.920	5.0E-32	-2.012
GNA12	1.1E-14	1.183	6.0E-33	1.415
GNA14	6.5E-07	-1.299	1.7E-97	-2.759
GNE	2.2E-04	-1.279	9.1E-29	-1.554
GNG7	2.0E-05	-1.986	1.7E-64	-2.460
GNLY	7.6E-09	2.772	7.3E-11	1.993
GOLGA7B	8.0E-08	2.382	3.5E-06	1.325
GPD1L	6.9E-11	-2.508	3.9E-112	-2.600
GPR153	5.7E-09	1.814	3.5E-10	1.158
GPR176	3.1E-11	2.096	6.4E-25	2.606
GPR39	2.4E-07	2.264	1.6E-14	2.353
GPR68	1.6E-08	1.236	1.1E-06	1.060
GPRASP1	5.4E-11	-2.050	9.2E-46	-1.676
GPRC5B	1.2E-07	-2.197	2.3E-17	-1.501
GPRC5C	3.6E-04	-1.158	5.6E-10	-1.029
GPT2	1.4E-08	-1.512	1.5E-40	-1.697
GPX3	4.0E-11	-2.466	2.9E-38	-2.313
GUCY2C	1.0E-09	-1.230	1.0E-05	-1.096
GULP1	4.7E-15	-2.594	6.4E-19	-1.719
GZMB	8.0E-09	2.387	1.7E-06	1.340
HADH	4.7E-09	-1.593	7.6E-29	-1.075
HAGHL	7.3E-08	1.182	1.3E-12	1.543
HAP1	5.7E-04	2.023	6.8E-22	3.112
HAVCR2	2.0E-06	1.514	2.1E-07	1.059
HBB	1.1E-03	-1.480	1.6E-26	-2.652
HELLS	1.1E-06	1.225	4.2E-15	1.238
HELZ2	7.2E-12	1.756	2.2E-10	1.132
HENMT1	2.2E-10	1.586	7.2E-14	1.374
HERC5	1.3E-08	1.962	4.0E-18	2.112
HID1	6.2E-04	-2.551	7.1E-22	-1.729
HIST1H2AI	1.6E-06	1.343	2.1E-16	2.593
HIST1H2AM	4.2E-07	1.443	1.6E-10	1.798

HIST1H2BH	7.6E-07	1.399	8.4E-13	1.830
HIST1H2BJ	5.5E-09	1.400	2.7E-12	1.753
HIST1H2BL	1.5E-06	1.458	1.3E-08	1.664
HIST1H2BN	2.0E-06	1.277	1.2E-14	1.683
HIST1H2BO	1.3E-08	1.902	9.2E-18	3.167
HIST1H4I	1.5E-06	1.574	2.6E-17	1.751
HJURP	4.2E-09	2.110	9.1E-29	1.772
HLF	2.1E-15	-3.327	2.5E-19	-2.071
HMGB3	1.1E-09	1.687	3.1E-17	1.302
HNMT	3.6E-04	-1.066	2.6E-17	-1.172
HOMER3	1.9E-11	1.619	2.0E-27	1.644
HOPX	6.6E-04	-1.243	4.4E-36	-2.520
HOXD10	4.1E-08	2.396	1.5E-30	3.806
HPGD	4.7E-09	-1.349	1.3E-48	-2.940
HSD11B2	9.3E-04	-1.133	1.4E-68	-2.462
HSD17B6	1.8E-09	2.225	4.1E-36	2.254
HSF2BP	5.2E-09	1.737	1.4E-16	1.779
HSPB6	3.8E-07	-3.608	3.3E-52	-3.962
HSPB7	2.8E-05	-3.064	3.6E-15	-2.683
HSPB8	2.8E-07	-1.877	1.8E-23	-1.821
HTR7	6.3E-14	3.585	1.1E-14	2.507
ICA1	5.6E-08	-2.585	9.2E-17	-1.547
ICAM5	2.5E-05	1.628	3.1E-28	2.881
ICOS	1.2E-09	2.489	5.7E-11	1.611
ID4	5.3E-15	-2.231	4.2E-32	-1.856
IDO1	6.5E-11	3.763	1.2E-07	2.218
IER5L	9.1E-11	1.432	1.3E-17	1.253
IFI27	1.1E-09	1.687	7.6E-20	2.097
IFI30	1.8E-10	1.825	1.7E-17	1.488
IFI35	2.6E-11	2.124	2.3E-16	1.571
IFI44	6.9E-12	2.252	1.6E-18	2.223
IFI6	7.5E-11	2.072	1.3E-22	3.009
IFIH1	1.8E-11	1.876	1.7E-12	1.430
IFIT1	1.8E-09	1.683	1.6E-12	2.213
IFIT2	1.1E-05	1.804	4.2E-14	2.186
IFIT3	7.5E-11	2.500	2.9E-13	2.139
IFITM1	8.1E-09	1.361	1.3E-09	1.276
IFNG	7.5E-06	1.735	2.2E-07	2.134
IGDCC4	4.1E-04	-1.890	3.2E-22	-1.801
IGF2BP2	4.1E-11	2.570	2.1E-24	2.481
IGF2BP3	1.3E-03	2.158	8.9E-10	2.068
IGFBP3	2.2E-04	1.380	1.1E-07	1.423

IKZF2	5.0E-11	-1.720	6.9E-35	-1.837
IKZF3	2.2E-03	1.240	6.7E-05	1.085
IL12RB1	6.7E-07	1.856	1.8E-08	1.350
IL12RB2	3.0E-07	2.961	4.4E-10	1.988
IL17D	5.8E-03	-1.592	2.1E-66	-2.895
IL17RD	4.8E-10	-1.726	4.2E-21	-1.455
IL18BP	1.4E-07	1.451	7.7E-10	1.236
IL1A	8.7E-06	1.783	3.7E-09	2.086
IL21R	4.9E-09	2.060	1.3E-10	1.614
IL27RA	9.7E-07	1.075	2.7E-08	1.163
IL2RA	7.2E-08	1.990	3.6E-12	1.537
IL31RA	9.8E-06	1.741	5.5E-07	1.410
IL34	7.9E-13	-2.445	1.9E-22	-1.712
IL7R	1.2E-08	1.987	8.0E-12	1.580
INHBA	4.2E-15	4.652	6.3E-30	4.034
IQGAP2	1.4E-02	-1.098	5.8E-21	-1.665
IQGAP3	2.4E-07	1.488	3.7E-20	1.465
IRF7	3.2E-13	2.102	2.1E-08	1.047
ISG15	3.4E-13	3.152	3.5E-26	3.444
ISM1	3.9E-07	-1.193	6.8E-13	-1.350
ITGA11	1.4E-04	1.269	1.3E-09	1.980
ITGA3	7.5E-14	2.367	3.0E-16	1.683
ITGA5	8.9E-08	1.492	5.2E-24	2.327
ITGA6	3.5E-11	1.906	2.5E-23	2.009
ITGA7	6.8E-06	-2.305	6.0E-12	-1.478
ITGAV	2.6E-06	1.054	2.3E-12	1.111
ITGB4	4.8E-09	1.620	1.8E-15	1.344
ITGB6	6.3E-08	2.147	2.2E-07	1.257
ITM2A	5.1E-10	-1.705	2.9E-57	-2.294
JAK3	4.7E-05	1.115	1.5E-07	1.010
JAKMIP1	1.4E-04	1.307	4.0E-06	1.199
JAM2	1.6E-07	-1.106	3.6E-16	-1.225
JPH2	2.1E-03	-2.566	3.0E-23	-2.420
KALRN	1.1E-05	-1.183	6.2E-30	-1.583
KCNAB1	4.1E-09	-1.257	5.7E-59	-1.712
KIAA1211L	4.5E-09	-1.862	2.0E-23	-1.671
KIAA1324	9.1E-03	-5.323	9.5E-28	-2.817
KIAA1549L	2.1E-03	1.410	1.9E-10	1.857
KIF11	2.0E-06	1.264	5.0E-17	1.077
KIF14	9.5E-10	2.487	3.6E-35	1.941
KIF18A	1.6E-09	2.088	2.0E-26	1.569
KIF18B	1.7E-07	1.762	3.9E-31	1.762

KIF20A	3.0E-09	2.122	2.8E-17	1.370
KIF23	5.7E-09	2.190	1.6E-33	1.586
KIF2C	2.4E-10	2.209	6.6E-39	1.879
KIF4A	3.2E-09	2.062	8.6E-34	1.721
KIT	4.0E-06	-1.016	1.5E-30	-2.074
KLB	2.7E-13	-1.734	8.0E-32	-2.085
KLF15	3.1E-11	-4.367	1.2E-44	-2.947
KLF7	7.8E-11	1.375	8.4E-24	1.594
KLF8	1.4E-04	-1.083	2.5E-08	-1.004
KLF9	3.8E-06	-1.167	1.6E-16	-1.149
KLHDC7B	3.3E-08	1.860	2.7E-14	3.202
KLHL31	1.6E-04	-2.894	7.0E-42	-2.842
KLRD1	6.7E-04	1.704	8.0E-06	1.124
KMO	1.1E-05	1.261	3.8E-14	1.707
KNSTRN	1.5E-10	1.336	1.6E-24	1.150
KPNA2	8.4E-11	1.523	5.9E-30	1.212
KREMEN2	6.1E-06	2.191	6.8E-18	2.849
KRT13	7.0E-05	-2.545	1.1E-16	-2.672
KRT14	4.2E-04	1.278	1.9E-06	1.300
KRT17	1.3E-07	2.390	3.0E-18	2.078
KY	5.4E-06	-3.867	1.7E-49	-3.725
KYNU	3.7E-11	2.641	2.5E-09	1.786
LAG3	2.5E-09	2.558	4.8E-13	2.085
LAMA1	1.2E-08	2.575	4.2E-12	2.760
LAMA3	1.7E-09	2.834	2.7E-22	2.788
LAMB3	2.0E-05	1.776	4.0E-21	1.969
LAMC2	4.9E-12	4.261	8.1E-32	3.777
LAMP3	2.2E-07	2.076	2.2E-13	1.658
LAX1	6.8E-03	1.232	2.3E-05	1.195
LCA5	8.8E-08	-1.156	1.7E-14	-1.022
LDB3	1.4E-05	-4.997	2.8E-53	-4.283
LDHD	4.8E-06	-1.993	1.5E-50	-2.411
LDOC1	6.3E-10	-2.887	3.9E-07	-1.153
LIFR	6.9E-12	-2.750	2.6E-10	-1.309
LILRB1	8.4E-07	1.775	4.1E-06	1.007
LILRB4	2.5E-06	1.644	4.2E-09	1.617
LIMCH1	1.2E-05	-1.659	6.0E-31	-2.157
LINGO4	1.0E-09	-3.631	2.2E-42	-3.766
LIPG	8.0E-07	1.522	4.6E-04	1.049
LIPH	1.8E-03	-1.711	2.3E-06	-1.111
LMOD1	1.5E-09	-2.790	1.9E-58	-2.612
LNX1	3.3E-08	-1.188	3.6E-27	-1.534

LOX	2.5E-06	1.661	1.1E-08	1.443
LPCAT1	7.7E-14	1.654	1.2E-26	1.648
LPIN1	2.3E-04	-1.074	2.1E-57	-1.697
LPL	1.1E-08	-2.637	1.1E-26	-2.192
LRFN4	2.1E-11	1.646	3.2E-21	1.374
LRP8	5.4E-07	1.166	3.7E-10	1.138
LRRC15	3.7E-10	2.488	1.6E-11	2.646
LRRK2	8.1E-10	-1.500	4.3E-21	-1.529
LRRN4CL	1.6E-11	-2.719	5.5E-66	-2.649
LTBP1	7.6E-12	1.919	2.3E-14	1.747
LTF	1.5E-05	-3.436	1.0E-24	-3.829
LUM	7.5E-08	1.218	3.9E-13	1.996
LY6E	4.4E-10	1.816	1.4E-13	1.343
LY6K	1.5E-07	2.723	5.4E-21	2.262
LYPD1	9.4E-15	3.523	5.5E-08	1.619
LYVE1	8.8E-09	-2.049	7.0E-36	-2.210
MACROD1	1.2E-06	-2.223	9.8E-31	-1.469
MAGI1	4.9E-13	-1.481	3.2E-56	-1.518
MAL	1.5E-08	-3.729	1.8E-52	-4.388
MAMDC2	1.2E-12	-2.870	1.5E-14	-1.759
MAML3	4.6E-11	-1.600	5.1E-32	-1.616
MAMSTR	3.3E-09	-1.618	7.0E-12	-1.101
MAN1C1	5.5E-07	-1.328	3.1E-21	-1.324
MANSC1	3.9E-09	-2.147	9.1E-20	-1.493
MAOB	5.5E-13	-3.830	4.5E-09	-1.551
MAP6	1.3E-07	-1.538	4.3E-22	-1.981
MARC2	3.3E-09	-2.164	1.0E-14	-1.228
MARCKSL1	9.2E-10	1.347	5.8E-14	1.143
MARVELD1	1.2E-10	1.409	2.7E-19	1.315
MATN3	4.5E-09	2.253	7.8E-21	3.025
MB	3.6E-04	-3.557	2.4E-36	-3.983
MC1R	1.6E-10	2.713	6.6E-21	1.433
MCM10	4.0E-09	1.951	1.4E-20	1.398
MCM2	1.2E-09	1.349	2.1E-25	1.914
MCM4	3.7E-08	1.296	2.3E-19	1.191
ME3	3.0E-07	-1.780	2.6E-18	-1.358
MEF2C	1.6E-02	-1.264	1.1E-12	-1.567
MEFV	9.6E-07	1.309	1.2E-09	1.724
MEIS1	7.1E-10	-2.081	2.3E-18	-1.249
MEIS2	7.5E-08	-1.579	3.3E-29	-1.254
MELK	6.5E-09	2.304	4.1E-24	1.697
MET	4.0E-11	1.611	2.2E-12	1.116

METTL24	1.0E-11	-2.045	4.7E-03	-1.047
METTL7A	5.7E-09	-1.798	3.5E-35	-2.036
MFAP2	1.1E-16	3.020	2.7E-38	2.954
MFAP4	9.1E-12	-2.793	4.2E-17	-1.834
MGAT4A	6.5E-03	-1.029	2.1E-19	-1.188
MGLL	1.5E-10	-1.652	4.1E-38	-1.840
MGP	7.5E-10	-2.393	2.8E-21	-2.149
MICAL2	6.3E-12	2.063	6.9E-19	1.848
MITF	2.3E-08	-2.088	1.3E-39	-1.882
MKI67	4.0E-07	1.740	5.8E-16	1.261
MLANA	1.3E-07	-2.251	1.4E-47	-2.558
MLPH	6.8E-10	-4.518	2.9E-31	-2.370
MMP1	3.2E-18	7.212	2.0E-11	2.813
MMP11	2.6E-14	5.777	4.3E-35	5.198
MMP12	3.7E-13	4.850	4.3E-28	3.899
MMP13	4.8E-16	9.157	2.9E-22	6.366
MMP14	3.0E-11	1.626	3.1E-25	1.602
MMP17	7.5E-08	1.738	1.4E-12	1.438
MMP3	2.0E-09	2.259	1.6E-10	2.736
MMP9	7.0E-12	3.337	6.5E-27	3.787
MMRN1	1.5E-08	-1.878	1.3E-31	-2.506
MOB3B	8.1E-10	1.373	5.0E-08	1.086
MPC1	1.9E-08	-1.492	1.0E-58	-1.467
MPP7	9.6E-06	-1.041	5.1E-23	-1.383
MPZ	2.0E-12	-3.446	5.5E-26	-2.053
MSC	7.2E-05	1.259	2.4E-10	1.619
MSN	4.5E-13	1.474	8.8E-22	1.308
MTBP	4.7E-10	1.679	2.1E-36	1.693
MTFR2	3.7E-09	1.721	2.2E-25	1.719
MX1	2.2E-10	1.759	2.5E-08	1.323
MX2	4.6E-09	1.874	1.7E-09	1.435
MYBL2	2.6E-08	2.409	1.6E-37	2.105
MYH10	1.2E-07	1.081	2.1E-09	1.041
MYH11	8.7E-10	-3.584	2.1E-15	-2.099
MYH14	8.1E-10	-2.574	4.4E-07	-1.121
MYO10	2.8E-10	1.472	8.5E-21	1.275
MYO1B	1.9E-13	2.169	1.2E-23	1.716
MYO3B	2.0E-05	2.073	1.5E-08	2.030
MYO5C	2.9E-08	-2.641	3.8E-15	-1.593
MYO7A	2.0E-06	1.152	1.5E-16	1.519
MYOM1	1.2E-05	-4.234	3.6E-54	-3.994
MYOT	3.6E-04	-3.829	2.1E-29	-3.562

N4BP3	1.7E-07	-1.413	1.8E-36	-1.529
NAALADL2	2.8E-09	-3.643	1.0E-14	-1.633
NAGS	7.2E-10	1.949	1.0E-13	1.421
NBEA	1.2E-08	-2.947	1.4E-42	-2.593
NCALD	2.4E-02	-1.796	2.3E-11	-1.243
NCAPG	1.3E-09	1.976	9.0E-24	1.400
NCAPH	2.3E-09	1.918	5.8E-27	1.493
NDC80	1.1E-09	2.020	5.6E-25	1.828
NDNF	1.9E-12	-2.934	7.7E-14	-1.795
NDRG1	8.4E-05	1.122	6.8E-14	1.545
NDRG2	2.0E-07	-2.476	2.8E-75	-2.615
NEB	1.1E-03	-3.233	9.0E-15	-2.610
NEBL	8.6E-07	-1.478	1.2E-18	-1.432
NEFL	2.3E-08	4.086	9.5E-08	2.146
NEK2	1.1E-06	1.646	4.7E-28	1.742
NELL2	1.5E-09	4.397	1.8E-17	3.092
NETO2	3.5E-07	1.409	1.5E-38	1.958
NFIA	5.6E-16	-1.393	2.6E-26	-1.101
NFIB	1.1E-07	-1.520	1.8E-10	-1.060
NFIC	1.5E-10	-1.262	3.0E-19	-1.002
NFIX	2.1E-14	-2.103	4.8E-52	-1.841
NGF	1.9E-07	2.094	4.8E-05	1.392
NID1	5.8E-09	1.669	1.3E-15	2.038
NID2	4.3E-09	1.516	1.1E-15	2.242
NKX3-1	6.1E-03	-4.533	9.5E-19	-1.604
NLGN4X	4.6E-04	2.649	1.0E-05	1.770
NNAT	1.9E-06	-1.162	1.8E-16	-1.571
NOSTRIN	3.1E-06	-2.279	1.7E-112	-2.393
NPDC1	1.6E-08	-2.170	4.9E-15	-1.133
NPNT	1.0E-03	1.363	3.1E-17	1.861
NR3C2	8.7E-12	-3.339	3.1E-49	-2.982
NR4A1	5.7E-06	-2.008	5.3E-20	-1.815
NR4A2	8.7E-07	-1.888	4.8E-06	-1.076
NR4A3	3.2E-02	-1.157	1.0E-14	-1.609
NREP	2.1E-09	1.405	9.3E-16	1.475
NRG1	1.7E-06	1.954	1.2E-12	2.063
NRG2	2.1E-15	-3.445	1.3E-97	-3.878
NRIP3	1.3E-08	2.146	1.7E-28	2.743
NT5E	1.1E-07	1.860	5.1E-09	1.626
NTN4	1.0E-08	-1.275	2.8E-16	-1.213
NTRK3	8.0E-13	-3.097	1.3E-27	-2.587
NUSAP1	1.5E-09	1.426	7.9E-17	1.324

NXPH3	9.4E-13	-2.361	1.3E-13	-1.332
NYNRIN	6.7E-09	-1.485	2.1E-12	-1.250
OAS2	9.9E-12	2.353	1.4E-15	1.767
OAS3	1.1E-12	2.217	1.9E-14	1.644
OASL	4.4E-11	2.653	6.3E-15	2.456
OBSCN	2.2E-04	-3.678	1.3E-59	-2.810
OCIAD2	7.7E-11	1.960	1.1E-16	1.297
OCLN	2.5E-05	-1.372	5.8E-16	-1.535
ODC1	6.5E-05	1.097	6.5E-11	1.990
OGN	3.3E-10	-2.429	1.5E-13	-2.216
OIP5	1.1E-07	1.431	3.1E-18	1.449
OLFML2B	3.5E-09	1.469	1.1E-09	1.514
OLR1	5.1E-07	2.398	7.0E-10	2.083
ORC1	5.7E-08	1.785	8.9E-22	1.461
ORC6	2.0E-11	1.866	5.5E-29	1.758
OSR1	1.2E-11	-3.545	1.6E-32	-2.283
PAIP2B	5.5E-11	-2.401	1.4E-59	-1.977
PALM	2.4E-11	-2.152	5.2E-10	-1.219
PALMD	9.3E-09	-1.547	3.0E-16	-1.175
PAQR8	5.2E-04	-1.156	2.5E-20	-1.349
PARP12	4.8E-16	2.146	2.9E-29	1.520
PARP14	1.6E-12	1.741	1.2E-21	1.444
PATL2	4.3E-04	1.329	9.8E-10	1.302
PAX9	4.1E-09	-2.547	2.9E-34	-2.264
PBX1	4.0E-14	-2.507	1.2E-11	-1.321
PCCA	7.8E-11	-1.522	1.3E-26	-1.076
PCDH17	8.9E-13	2.173	4.5E-13	1.421
PCDH7	5.0E-07	1.516	1.5E-05	1.148
PCED1B	8.2E-09	1.349	3.1E-21	1.481
PDCD1LG2	4.8E-08	2.053	1.3E-13	2.098
PDE1A	1.6E-07	-1.788	1.2E-23	-1.401
PDE4DIP	2.2E-03	-2.179	3.2E-36	-1.992
PDE8B	6.2E-06	-2.863	1.2E-19	-1.073
PDGFD	5.2E-08	-1.770	9.0E-16	-1.513
PDK4	2.1E-08	-3.124	4.6E-56	-3.667
PDLIM3	4.8E-05	-2.309	3.7E-13	-1.950
PDPN	8.3E-13	2.975	1.5E-20	2.098
PDZRN3	1.1E-07	-1.566	5.7E-13	-1.303
PEG3	3.8E-11	-3.286	3.4E-28	-2.708
PER2	5.7E-11	-1.123	2.3E-17	-1.061
PFKFB1	4.8E-06	-3.557	9.5E-55	-2.563
PFKM	4.5E-02	-1.894	5.6E-47	-1.708

PFN2	1.1E-07	1.135	2.5E-08	1.239
PGF	2.3E-08	1.835	6.9E-15	1.763
PGM1	2.6E-04	-1.558	1.5E-33	-1.198
PGM5	2.1E-09	-2.837	2.2E-43	-2.582
PGR	7.5E-08	-2.342	3.3E-06	-1.349
PHKA1	2.4E-05	-1.569	8.3E-22	-1.153
PHLDB2	1.6E-11	1.359	4.8E-21	1.777
PHYH	4.8E-06	-2.629	7.0E-62	-2.122
PHYHD1	7.9E-13	-2.944	1.2E-04	-1.074
PI3	3.3E-04	1.124	4.3E-03	1.094
PIEZO2	7.8E-07	1.258	2.6E-10	1.616
PIF1	2.6E-10	1.986	1.2E-23	1.940
PIK3CD	3.0E-09	1.272	6.5E-15	1.187
PIK3R1	1.4E-07	-1.030	2.0E-20	-1.257
PIP5KL1	6.5E-05	1.444	1.3E-08	1.158
PITX1	1.8E-05	-1.382	3.9E-16	-1.363
PITX2	7.0E-09	-2.606	8.2E-06	-1.233
PKIA	8.4E-05	-3.011	1.9E-43	-2.864
PKMYT1	2.7E-11	2.127	2.5E-34	1.832
PKNOX2	8.4E-07	-1.963	1.2E-30	-2.471
PLA2G7	1.5E-11	3.059	1.2E-19	2.090
PLAC9	3.4E-13	-2.648	2.2E-82	-2.850
PLAU	1.5E-13	2.676	2.9E-28	2.508
PLCB4	2.9E-08	-3.979	8.5E-26	-2.331
PLEK2	1.4E-07	2.341	1.4E-17	1.894
PLEKHA6	9.2E-09	-1.953	2.9E-32	-2.030
PLEKHA7	1.4E-07	-1.500	4.4E-27	-1.611
PLEKHB1	3.3E-06	-3.524	2.0E-73	-3.209
PLEKHG6	1.3E-09	-2.390	1.7E-08	-1.140
PLIN4	3.7E-13	-4.147	4.1E-120	-4.487
PLK1	1.0E-09	2.410	2.9E-29	1.613
PLK4	2.4E-07	1.353	4.4E-21	1.197
PLLP	2.9E-12	-2.306	8.9E-15	-1.232
PLOD1	7.5E-13	1.251	9.2E-25	1.411
PLOD2	1.2E-04	1.578	1.2E-18	1.673
PLXNA1	1.1E-11	1.364	1.6E-20	1.331
PMEL	4.4E-07	-4.208	5.0E-65	-2.699
PMEPA1	4.6E-09	1.535	4.8E-22	2.194
PMFBP1	3.0E-06	1.411	1.4E-06	1.075
PNPLA7	8.2E-11	-3.150	1.9E-58	-2.050
PODN	1.7E-09	-2.329	7.3E-13	-1.593
POLQ	1.0E-07	1.485	3.1E-22	1.657

POPDC2	3.4E-03	-3.093	3.9E-46	-2.495
POSTN	2.0E-10	3.209	1.2E-20	3.470
POU2F2	4.6E-06	1.335	7.4E-12	1.354
PPARG	1.2E-10	-2.459	4.3E-32	-2.269
PPARGC1A	7.5E-10	-3.398	1.8E-39	-2.858
PPFIA1	5.7E-05	1.035	1.6E-10	1.466
PPIL6	5.0E-09	-1.754	1.4E-51	-2.266
PPL	1.9E-03	-1.288	5.3E-30	-1.888
PPM1H	1.5E-03	-2.734	1.2E-12	-1.518
PPM1L	3.8E-12	-2.125	1.8E-23	-1.572
PPP1R12B	1.4E-07	-2.101	3.3E-44	-2.043
PPP1R14C	2.9E-06	1.637	2.3E-08	1.043
PPP1R3C	3.0E-09	-3.739	2.6E-46	-2.971
PPP4R4	2.2E-11	3.945	4.8E-18	2.876
PRADC1	2.1E-09	-1.276	2.5E-22	-1.001
PRC1	5.5E-09	1.551	1.2E-21	1.225
PRELP	4.9E-11	-2.484	8.0E-08	-1.332
PREX2	9.9E-08	-1.233	1.7E-11	-1.033
PRH1	4.2E-04	-6.757	3.7E-158	-7.628
PRKAR2B	9.2E-07	-2.033	3.3E-11	-1.179
PRNP	1.4E-10	1.480	2.1E-14	1.139
PROCR	2.3E-12	2.060	6.6E-16	1.691
PRPH2	1.5E-05	-2.104	1.8E-15	-1.540
PRR15L	1.4E-05	-3.797	9.3E-26	-2.481
PRRT3	1.0E-07	-1.133	1.3E-26	-1.267
PRSS27	2.6E-03	-1.493	3.6E-37	-2.873
PRSS53	2.1E-05	1.489	2.8E-15	1.873
PRUNE2	1.9E-04	-2.285	5.5E-12	-1.904
PSRC1	5.3E-08	1.585	7.2E-18	1.289
PTGER3	3.7E-07	-1.543	3.9E-13	-1.522
PTGFR	2.0E-06	-1.351	2.0E-18	-1.890
PTGFRN	1.4E-09	1.421	2.4E-21	1.269
PTGS2	6.7E-04	1.281	2.3E-06	1.609
PTHLH	5.0E-11	3.666	1.4E-23	3.437
PTK7	1.5E-10	1.853	7.2E-31	1.572
PTN	6.5E-13	-3.045	1.5E-07	-1.371
PTPN7	1.6E-05	1.233	4.5E-08	1.229
PTPRN2	1.5E-04	-2.660	7.4E-26	-1.770
PTPRO	3.4E-04	1.025	1.0E-06	1.090
PTTG1	3.0E-06	1.271	9.9E-11	1.177
PXDN	3.2E-11	2.144	3.5E-15	2.029
PYGM	7.8E-06	-4.582	7.3E-69	-5.263

PYHIN1	5.6E-04	1.201	8.8E-05	1.000
RAB31	1.6E-11	1.520	5.6E-16	1.199
RAC2	1.6E-09	1.594	2.6E-18	1.579
RACGAP1	8.5E-08	1.206	2.4E-21	1.017
RAD51	1.4E-10	1.875	1.1E-26	1.530
RAD51AP1	1.1E-09	1.787	3.2E-28	1.779
RAG1	2.1E-07	2.742	1.8E-15	2.125
RAI14	4.7E-06	1.267	1.4E-09	1.078
RAI2	1.2E-10	-1.842	4.3E-20	-1.492
RASGEF1A	2.1E-04	2.390	1.8E-12	2.229
RASL11B	1.6E-06	1.497	2.8E-15	2.275
RBM20	1.7E-10	-3.170	5.3E-33	-2.314
RBP1	2.8E-07	2.617	1.9E-15	2.032
RBP7	1.2E-10	-2.014	1.0E-05	-1.006
RCAN2	5.1E-07	-2.370	3.5E-40	-2.179
RDH16	2.8E-07	1.206	3.4E-11	1.975
RECQL4	1.5E-10	1.595	1.0E-15	1.185
RELN	1.4E-08	-2.169	1.4E-12	-1.604
RERG	1.1E-09	-1.964	3.8E-13	-1.555
RFC4	1.8E-10	1.300	3.5E-29	1.876
RGMA	7.1E-08	-1.475	5.1E-08	-1.088
RGS11	1.6E-11	-1.941	1.2E-14	-1.412
RGS20	5.0E-07	2.179	7.3E-06	1.232
RGS4	9.9E-09	2.881	3.9E-19	2.730
RIBC1	5.7E-08	-1.079	3.3E-21	-1.452
RMND5B	5.0E-09	-1.130	3.1E-49	-1.309
RNASEH2A	4.1E-10	1.251	1.5E-21	1.302
RNF125	8.5E-07	-1.051	1.6E-13	-1.021
RNF150	7.8E-10	-2.618	9.9E-17	-1.998
RNF157	1.3E-03	-2.762	6.2E-26	-1.920
RNF180	3.8E-12	-2.240	5.0E-52	-2.007
ROR1	6.5E-12	-2.150	3.6E-12	-1.372
RRM2	1.0E-06	1.738	2.2E-13	1.090
RSAD2	4.5E-11	3.083	7.6E-16	2.586
RSPO3	1.4E-07	-2.298	6.9E-26	-2.042
RTKN	6.6E-10	1.129	7.6E-20	1.000
RTN2	1.1E-02	-1.848	1.0E-38	-1.742
RTN4R	4.7E-05	1.180	3.1E-21	1.611
RTP4	3.3E-07	1.778	7.0E-10	1.497
RUNDC3A	1.0E-04	2.190	4.5E-07	1.535
RUNX3	1.4E-07	1.217	1.0E-10	1.148
RYR1	1.1E-02	-2.664	1.4E-34	-2.524

RYR2	9.9E-04	-2.764	3.1E-03	1.021
RYR3	9.1E-04	-2.123	2.2E-08	-1.332
SAMD5	4.1E-05	-2.104	3.8E-27	-2.276
SASH1	4.3E-09	-1.169	1.2E-51	-2.107
SATB1	1.3E-05	-1.124	2.1E-22	-1.275
SCARA3	2.5E-08	-1.304	1.2E-16	-1.418
SCG5	4.6E-08	2.428	3.8E-17	2.565
SCIN	1.5E-08	-3.850	2.2E-48	-2.845
SCNN1D	5.6E-05	2.726	4.0E-07	1.677
SCRG1	1.0E-09	-1.376	1.6E-13	-2.350
SDK2	7.3E-07	1.715	3.1E-11	1.800
SELENBP1	2.3E-11	-3.381	4.3E-55	-2.696
SEMA3E	7.7E-10	-3.953	2.5E-09	-2.166
SEMA3G	1.2E-07	-1.581	5.1E-32	-1.790
SEMA6C	6.5E-04	-3.088	1.7E-42	-2.390
SEMA7A	1.5E-08	1.657	2.8E-09	1.132
SERPINE1	3.0E-11	2.560	1.0E-20	2.934
SERPINE2	4.0E-08	2.293	4.4E-08	1.465
SERPINH1	4.2E-15	2.061	2.0E-34	2.196
SESN1	9.5E-07	-1.231	2.7E-28	-1.038
SETMAR	1.1E-11	-1.163	1.8E-25	-1.058
SFRP1	8.1E-05	-1.189	2.0E-13	-1.985
SFXN3	5.1E-10	1.178	9.8E-14	1.067
SGCD	1.5E-04	-1.400	2.6E-05	-1.120
SGIP1	6.6E-10	1.478	5.3E-20	1.989
SH2D2A	1.4E-11	2.467	7.3E-29	2.198
SH3BGR	7.6E-05	-1.863	5.1E-80	-3.315
SH3BGRL2	4.2E-15	-3.599	3.8E-111	-3.548
SHCBP1	3.7E-10	1.999	6.7E-30	1.599
SHISA4	6.5E-04	-2.739	1.2E-26	-1.855
SHROOM3	2.6E-06	-2.193	9.9E-45	-2.046
SIGLEC7	4.9E-06	1.379	5.1E-08	1.280
SIM2	1.3E-06	-2.798	1.6E-04	-1.045
SIRPB1	3.2E-06	1.303	2.1E-07	1.429
SKA1	5.0E-08	2.008	3.9E-18	1.385
SKA3	1.2E-08	1.709	2.1E-13	1.080
SLAIN1	4.0E-06	-1.735	3.2E-10	-1.249
SLAMF8	8.2E-09	1.898	1.1E-13	1.578
SLC12A2	3.3E-02	-2.257	2.3E-34	-1.506
SLC13A4	1.2E-07	-1.482	5.8E-14	-1.653
SLC15A3	7.8E-11	1.999	3.2E-21	1.860
SLC16A1	1.4E-10	2.084	4.8E-18	1.347

SLC16A6	2.6E-05	-1.948	7.8E-41	-2.158
SLC16A7	6.1E-09	-1.327	1.7E-33	-2.060
SLC19A3	2.3E-05	-1.427	3.3E-22	-1.827
SLC1A1	9.9E-09	-1.449	3.0E-15	-1.653
SLC20A1	2.6E-10	1.357	5.9E-21	1.149
SLC24A3	1.0E-05	-1.269	4.7E-08	-1.051
SLC25A21	1.0E-12	-2.803	1.9E-08	-1.119
SLC25A23	4.7E-12	-1.590	1.1E-28	-1.539
SLC25A25	2.4E-06	-1.178	2.8E-29	-1.505
SLC25A4	7.5E-06	-3.538	6.0E-86	-2.883
SLC26A2	7.9E-07	-2.437	3.4E-22	-1.322
SLC26A6	1.8E-12	1.454	1.1E-33	1.466
SLC2A1	5.2E-06	1.593	2.9E-19	1.842
SLC2A10	1.5E-02	-1.591	2.1E-09	-1.100
SLC2A6	8.8E-10	1.636	1.3E-31	2.228
SLC37A1	1.0E-08	-1.498	4.6E-28	-1.428
SLC38A5	8.3E-06	1.933	3.0E-08	1.550
SLC3A2	2.3E-11	1.235	1.2E-16	1.041
SLC44A3	1.7E-04	-1.593	2.4E-28	-1.491
SLC7A11	6.8E-05	1.162	8.4E-07	1.717
SLC7A2	1.1E-09	-2.633	2.2E-06	-1.171
SLC7A5	1.6E-06	1.337	3.6E-09	1.204
SLC7A7	3.0E-06	1.283	3.1E-09	1.172
SLC7A8	4.2E-09	1.845	8.4E-13	1.730
SLC9A4	2.5E-08	-1.457	2.7E-11	-2.539
SLCO5A1	1.0E-03	-2.196	3.5E-32	-2.476
SLIT3	2.7E-11	-2.433	1.5E-10	-1.291
SMAD9	5.9E-07	-2.717	2.5E-28	-1.569
SMARCD3	2.0E-07	-2.152	5.9E-34	-1.689
SMIM5	3.6E-07	-1.636	5.4E-42	-2.608
SMS	3.3E-10	1.134	1.0E-15	1.029
SMTNL1	1.9E-05	2.094	2.0E-53	-4.055
SNAI2	8.7E-12	2.046	2.2E-28	1.761
SNAPC1	4.0E-08	1.045	3.3E-12	1.085
SNED1	4.8E-11	-1.796	4.5E-17	-1.496
SNRPN	1.2E-06	-1.594	1.4E-16	-1.253
SNTA1	1.8E-04	-1.987	5.0E-26	-1.427
SNTB1	5.6E-04	-1.171	5.3E-33	-1.607
SNX10	4.8E-11	1.973	1.3E-33	2.194
SOBP	1.1E-06	-2.091	1.7E-16	-1.438
SOCS1	2.8E-09	1.671	9.8E-23	1.871
SOD3	4.5E-11	-1.829	6.8E-32	-1.729

SORBS1	5.1E-11	-2.856	9.9E-68	-2.769
SORBS2	2.3E-08	-2.197	5.0E-60	-2.818
SORCS2	1.0E-08	1.900	1.5E-04	1.023
SOX17	1.7E-04	-1.341	3.6E-25	-1.467
SP110	1.0E-14	1.871	7.2E-11	1.186
SP140	2.1E-05	1.482	1.4E-10	1.623
SPAG16	8.5E-09	-1.687	8.1E-13	-1.065
SPAG5	2.7E-09	1.737	1.2E-30	1.635
SPARC	5.1E-09	1.147	3.3E-17	2.137
SPATA18	1.2E-08	-1.633	2.6E-51	-2.193
SPATA6	5.8E-13	-2.282	4.3E-36	-1.728
SPC24	4.8E-08	1.654	5.2E-21	1.677
SPEF2	2.6E-09	-1.720	7.1E-08	-1.099
SPINK5	1.3E-02	-1.812	4.1E-35	-2.771
SPP1	7.5E-08	3.107	1.4E-10	2.807
SPRY4	7.4E-12	2.091	2.7E-23	1.705
SPTB	1.2E-02	-1.537	1.6E-22	-2.625
ST3GAL4	1.1E-04	-1.201	4.5E-27	-1.298
STARD4	3.3E-08	1.390	1.0E-21	1.427
STAT1	9.4E-15	1.965	3.3E-14	1.498
STAT2	5.3E-13	1.431	7.2E-17	1.082
STC2	4.2E-11	2.315	1.2E-28	3.103
STIL	1.4E-09	1.668	1.9E-22	1.338
STK39	6.2E-04	-1.062	1.6E-40	-1.315
STON2	9.4E-08	1.432	6.3E-13	1.213
STRA6	1.5E-05	2.010	1.9E-11	2.097
STX19	3.3E-06	-1.185	5.2E-08	-1.167
STX1A	2.4E-12	1.710	6.9E-29	1.900
SUCNR1	2.2E-06	1.515	3.8E-06	1.046
SULF1	1.5E-07	2.379	3.7E-14	2.179
SULF2	9.0E-07	1.589	1.1E-09	1.248
SVEP1	3.2E-06	-1.182	1.9E-13	-1.154
SVIL	2.2E-05	-1.663	4.6E-31	-1.344
SVIP	7.1E-10	-2.088	8.0E-13	-1.444
SYBU	1.6E-08	-1.992	1.1E-09	-1.247
SYNGR1	2.2E-12	-2.320	2.5E-28	-1.745
SYNM	1.1E-04	-2.830	1.6E-46	-2.465
SYNPO2	8.8E-07	-2.280	2.2E-09	-1.734
SYT12	7.5E-08	2.574	6.5E-07	1.456
SYT8	4.5E-06	-3.035	3.4E-11	-1.785
SYTL4	2.4E-11	-2.080	6.3E-18	-1.147
TACC3	1.0E-09	1.312	3.0E-23	1.261

TACR1	4.6E-10	-2.022	4.9E-24	-2.325
TBX15	3.9E-04	-2.905	1.8E-33	-2.522
TC2N	1.6E-06	-1.254	7.2E-14	-1.115
TCEA3	3.8E-09	-3.163	1.2E-23	-1.691
TCP11L2	3.6E-10	-1.399	1.5E-36	-1.462
TDRD6	5.7E-11	2.359	7.5E-14	1.560
TENM1	1.5E-07	-1.151	3.2E-07	2.482
TENM2	1.3E-08	2.488	2.1E-14	2.301
TENM3	2.9E-07	1.708	1.4E-14	2.140
TESC	5.8E-04	-2.798	3.9E-82	-3.499
TGFB1	3.3E-09	1.525	4.6E-39	1.603
TGFBI	9.2E-13	2.665	9.0E-27	3.375
TGFBR3	4.8E-14	-2.495	2.4E-39	-2.157
THBS2	4.8E-11	1.989	3.5E-10	1.783
THSD4	1.0E-04	-1.010	3.4E-31	-1.708
THY1	2.2E-10	1.507	1.3E-09	1.458
TIGIT	2.0E-06	1.551	5.6E-12	1.673
TK1	1.4E-08	1.687	1.5E-32	1.805
TLE2	9.5E-14	-3.244	1.6E-52	-2.486
TMC7	1.2E-12	2.587	1.4E-18	1.760
TMEM132A	1.6E-11	2.228	1.6E-24	1.504
TMEM132B	6.8E-09	-1.476	1.6E-06	-1.009
TMEM143	1.1E-04	-2.001	1.2E-50	-1.624
TMEM150B	2.3E-05	1.484	1.7E-15	1.954
TMEM170B	2.7E-04	-1.379	2.2E-16	-1.181
TMEM182	2.8E-02	-2.550	7.5E-52	-2.171
TMEM184B	2.2E-10	1.035	5.7E-26	1.020
TMEM200A	3.7E-04	1.220	3.8E-09	1.574
TMEM200C	2.1E-03	-2.121	3.1E-07	-1.409
TMEM220	5.3E-10	-1.776	2.0E-23	-1.362
TMEM229B	1.2E-06	1.722	6.6E-08	1.320
TMEM232	1.8E-10	-2.075	2.6E-81	-3.055
TMEM38A	1.6E-04	-3.639	5.0E-55	-2.937
TMEM47	7.7E-07	-1.584	4.5E-13	-1.170
TMEM63A	1.2E-02	-1.416	6.3E-33	-1.140
TMOD1	7.5E-06	-2.823	2.6E-32	-2.887
TMPRSS11A	1.1E-05	-2.689	7.0E-09	-1.843
TNC	1.2E-10	2.424	2.5E-15	2.396
TNFRSF11A	8.2E-08	-1.721	1.8E-40	-1.845
TNFRSF12A	3.0E-12	1.883	1.0E-12	1.330
TNFRSF19	6.1E-07	-1.828	4.8E-17	-1.526
TNFRSF4	1.2E-07	1.836	2.6E-20	1.896

TNS1	3.2E-10	-1.990	2.8E-19	-1.416
TONSL	4.5E-09	1.449	1.3E-26	1.493
TOP2A	3.9E-07	1.664	1.4E-21	1.522
TOX	2.9E-05	1.109	2.6E-09	1.425
TP53INP2	8.7E-06	-1.148	9.7E-23	-1.451
TP63	1.5E-04	1.094	4.2E-13	1.148
TPBG	2.0E-13	1.907	3.8E-30	1.506
TPCN1	2.2E-10	-1.141	2.3E-42	-1.170
TPM2	6.0E-03	-2.271	2.1E-32	-2.375
TPPP	1.1E-10	-2.254	4.8E-09	-1.149
TPPP3	7.6E-04	-1.154	2.9E-10	-1.287
TPX2	1.7E-09	2.314	7.3E-33	1.830
TRAM2	8.5E-10	1.435	1.3E-29	1.646
TREM2	2.5E-10	2.731	7.4E-16	2.061
TRIL	2.5E-09	-1.807	3.1E-10	-1.308
TRIP13	6.3E-10	2.178	1.4E-27	1.765
TRPM2	1.1E-09	2.058	2.3E-13	1.515
TSPAN10	5.9E-07	2.438	5.5E-16	2.232
TSPAN12	2.4E-06	-2.984	9.4E-38	-2.068
TSPAN6	2.1E-05	-1.137	6.5E-27	-1.248
TTK	4.1E-07	1.624	2.6E-22	1.347
TTYH3	7.6E-13	1.555	1.0E-24	1.649
TXLNB	1.1E-03	-3.181	3.1E-37	-3.141
TYMS	7.1E-07	1.416	3.6E-14	1.342
UBE2C	4.5E-09	2.188	9.1E-33	1.881
UBE2L6	5.5E-13	1.871	1.7E-11	1.159
UBL3	1.9E-12	-1.239	1.1E-79	-1.566
UBXN10	4.4E-06	-2.108	2.6E-25	-2.333
UHRF1	2.3E-08	1.887	1.9E-22	1.591
UPK3B	1.3E-03	-2.339	9.4E-14	-1.841
USB1	1.2E-14	1.730	6.7E-26	1.188
USP13	2.2E-04	-2.168	3.7E-35	-1.830
USP18	7.0E-09	2.159	4.0E-18	1.840
VAV2	3.3E-13	1.981	7.6E-29	1.690
VEGFC	1.9E-09	2.412	6.3E-11	1.932
VWA2	1.7E-04	-1.034	1.0E-15	-1.643
WARS	2.0E-11	2.494	2.0E-11	1.932
WDHD1	4.8E-10	1.515	1.1E-35	1.547
WDR54	5.7E-12	1.904	1.7E-16	1.382
WDR66	1.6E-11	3.156	8.7E-20	1.852
WDR76	1.5E-08	1.321	2.1E-14	1.244
WDR78	4.1E-08	-1.610	7.8E-38	-2.094

WDR86	1.7E-05	1.317	2.8E-07	1.051
WNK4	1.7E-07	-3.841	1.3E-12	-1.751
XPR1	7.0E-14	1.388	6.5E-21	1.141
XRCC2	2.7E-08	2.542	5.1E-32	1.917
YEATS2	2.0E-10	1.009	9.7E-30	1.371
ZBP1	8.5E-06	1.903	4.5E-12	2.008
ZBTB16	1.5E-11	-4.036	2.3E-57	-3.774
ZBTB47	3.1E-06	-1.478	6.5E-37	-1.581
ZBTB7C	8.2E-11	-2.238	8.5E-09	-1.332
ZCWPW2	2.2E-09	-2.280	2.6E-29	-1.456
ZFP92	1.3E-05	1.230	1.5E-26	2.586
ZNF135	1.5E-09	-1.909	2.0E-13	-1.406
ZNF281	6.3E-09	1.025	8.2E-22	1.067
ZNF385D	6.2E-07	-1.309	8.5E-25	-1.819
ZNF415	1.1E-06	-2.146	1.0E-17	-1.567
ZNF423	8.9E-07	-1.542	1.4E-10	-1.155
ZNF433	6.9E-11	-1.573	6.0E-09	-1.089
ZNF469	1.7E-10	2.172	5.3E-18	2.579
ZNF471	1.3E-12	-2.556	1.1E-13	-1.494
ZNF568	1.9E-11	-1.845	5.4E-10	-1.046
ZNF582	4.3E-08	-1.539	1.5E-12	-1.173
ZNF626	5.6E-11	-2.078	1.8E-18	-1.553
ZNF677	8.0E-11	-2.008	4.2E-29	-1.786
ZNF737	9.7E-07	-1.349	1.9E-08	-1.064
ZNF844	4.3E-13	-2.215	4.0E-37	-1.959
ZNF853	2.3E-07	-2.060	2.1E-17	-1.605
ZNF880	1.4E-10	-2.085	1.1E-09	-1.056
ZSCAN18	3.7E-14	-2.333	4.1E-16	-1.486
ZWINT	2.0E-07	1.413	7.6E-19	1.220
ADCK3	4.9E-06	-3.282	2.0E-67	-2.613
ALS2CR11	2.3E-07	-1.491	2.1E-07	1.857
C11orf82	7.8E-11	1.942	1.6E-14	1.162
C14orf159	3.2E-06	-1.393	1.4E-47	-1.318
C16orf45	2.5E-06	-1.478	4.8E-18	-1.207
C16orf59	2.3E-09	1.865	9.3E-34	1.849
C5orf4	1.3E-09	-2.164	1.4E-43	-2.027
C7orf10	7.4E-09	1.887	7.3E-18	2.061
C7orf41	5.1E-08	-1.705	2.3E-21	-1.281
CASC5	8.9E-08	1.824	1.1E-18	1.253
CTSL2	8.5E-06	2.348	2.5E-14	1.881
DARC	2.4E-07	-1.462	4.1E-38	-2.670
DFNA5	2.1E-15	2.714	1.1E-23	2.125

DOPEY2	2.3E-07	-1.139	1.2E-30	-1.216
EFCAB4A	6.3E-04	-3.390	2.9E-57	-2.639
EFHA2	2.6E-09	-2.101	1.7E-10	-1.135
EMR2	4.0E-09	1.558	1.5E-12	1.451
FAM134B	1.0E-04	-1.849	3.0E-18	-1.615
FAM198A	8.0E-09	-1.185	1.8E-11	-1.447
FAM26F	1.4E-07	2.001	6.6E-09	1.492
FAM63A	7.2E-10	-1.190	1.5E-48	-1.421
FAM64A	8.3E-06	1.453	4.6E-22	1.833
FYB	1.8E-05	1.250	3.4E-06	1.160
GATS	5.0E-05	-1.704	4.3E-15	-1.163
GLT25D1	2.4E-11	1.332	6.1E-50	1.479
GPR133	1.1E-07	-1.551	1.3E-23	-1.908
GPR64	1.6E-09	-2.936	4.6E-16	-1.841
GSG2	1.9E-10	1.750	7.4E-19	1.411
GYLTL1B	5.9E-04	1.508	2.2E-08	1.112
KIAA1244	3.3E-07	-5.120	1.2E-23	-2.269
KIAA1456	3.7E-11	-2.303	1.1E-18	-1.864
KIAA1524	2.3E-11	1.752	2.8E-38	1.808
KIAA1644	4.6E-08	1.886	3.6E-14	2.464
KIAA1737	1.9E-07	-1.188	2.2E-57	-1.323
KIRREL	1.9E-12	1.383	2.9E-12	1.238
LEPRE1	2.3E-12	1.550	6.9E-28	1.826
LEPREL1	1.4E-03	1.451	3.0E-17	2.638
MB21D1	2.0E-13	2.147	2.2E-25	1.600
PARK2	4.2E-12	-2.798	3.9E-47	-2.476
PLA2G16	2.0E-04	-1.556	3.6E-10	-1.375
PPAP2B	3.6E-11	-1.826	3.8E-13	-1.107
PPAPDC3	1.2E-02	-2.955	6.4E-50	-2.865
PROSAPIP1	4.8E-03	-1.368	6.4E-12	-1.188
RLTPR	4.8E-04	1.014	2.1E-07	1.053
SDPR	5.4E-09	-1.945	2.9E-15	-1.637
SGOL1	4.7E-06	1.101	3.1E-13	1.097
SGOL2	5.5E-09	1.473	4.7E-18	1.102
SLMO1	8.7E-11	1.804	2.7E-32	2.081
TENC1	6.3E-10	-1.715	1.2E-30	-1.339
WBSCR17	3.5E-04	-1.266	1.6E-05	-1.131
WISP1	2.1E-13	2.669	2.6E-29	3.426
WISP2	3.0E-08	-2.464	2.6E-10	-1.768

Supplemental Table S5: Genes found significantly differentially expressed in OSCC-GB patients and also significantly dysregulated in triad samples

Gene symbol	OSCC-GB		Results from three-group comparison (TCC)		
	Corrected p-value	log2FC	p-value	FDR (q-value)	Rank
MMP11	2.64E-14	5.777	9.96E-51	1.31E-46	2
PLAU	1.49E-13	2.676	1.61E-44	1.41E-40	3
ETNK2	2.99E-08	-3.389	2.88E-44	1.90E-40	4
MFAP2	1.06E-16	3.020	1.22E-42	6.46E-39	5
CAB39L	2.36E-15	-2.395	4.56E-41	1.72E-37	7
LAMC2	4.87E-12	4.261	5.36E-40	1.77E-36	8
C1QTNF6	4.98E-16	3.043	3.65E-39	1.07E-35	9
PGAP3	1.47E-11	-1.114	2.14E-38	5.63E-35	10
PTK7	1.54E-10	1.853	1.37E-36	3.28E-33	11
ROR1	6.52E-12	-2.150	4.50E-36	9.13E-33	13
APLN	2.00E-09	2.226	6.73E-36	1.27E-32	14
EIF5A2	7.04E-10	1.798	7.45E-36	1.31E-32	15
TPCN1	2.24E-10	-1.141	9.19E-36	1.51E-32	16
TLE2	9.52E-14	-3.244	2.14E-35	3.33E-32	17
TGFBI	9.17E-13	2.665	3.16E-35	4.60E-32	18
COL4A1	2.10E-15	2.548	3.32E-35	4.60E-32	19
SERPINE1	2.97E-11	2.560	5.52E-35	7.28E-32	20
SERPINH1	4.18E-15	2.061	3.89E-34	4.88E-31	21
ITGA3	7.50E-14	2.367	6.22E-34	7.45E-31	22
PCDH17	8.86E-13	2.173	4.98E-33	5.47E-30	24
TNFRSF12A	2.99E-12	1.883	1.41E-32	1.43E-29	25
NRG2	2.10E-15	-3.445	1.41E-32	1.43E-29	26
PROCR	2.34E-12	2.060	2.30E-32	2.24E-29	27
WNK4	1.65E-07	-3.841	2.51E-32	2.36E-29	28
MAOB	5.50E-13	-3.830	2.85E-32	2.59E-29	29
MLPH	6.76E-10	-4.518	9.14E-32	8.04E-29	30
STC2	4.16E-11	2.315	1.01E-31	8.29E-29	32
PHC1	4.85E-09	-1.276	1.38E-31	1.10E-28	33
ID4	5.32E-15	-2.231	1.86E-31	1.40E-28	35
CCM2	7.03E-14	1.231	4.21E-31	2.92E-28	38
SHROOM3	2.57E-06	-2.193	4.69E-31	3.17E-28	39
WISP1	2.13E-13	2.669	1.04E-30	6.68E-28	41
ITGB6	6.26E-08	2.147	1.84E-30	1.10E-27	43
DCBLD1	4.33E-15	2.356	1.84E-30	1.10E-27	44
PLEKHA7	1.41E-07	-1.500	2.90E-30	1.70E-27	45
MAMDC2	1.16E-12	-2.870	8.98E-30	5.15E-27	46

TPBG	2.04E-13	1.907	9.59E-30	5.38E-27	47
PPP4R4	2.24E-11	3.945	1.14E-29	6.27E-27	48
FST	3.67E-10	2.777	1.40E-29	7.41E-27	50
DDX60L	1.46E-11	1.981	1.96E-29	9.61E-27	53
MICAL2	6.32E-12	2.063	1.97E-29	9.61E-27	54
MEIS1	7.12E-10	-2.081	2.37E-29	1.13E-26	55
SCIN	1.54E-08	-3.850	2.41E-29	1.13E-26	56
NELL2	1.45E-09	4.397	2.75E-29	1.27E-26	57
PANX1	5.08E-09	1.199	4.19E-29	1.87E-26	59
BAK1	1.54E-12	1.577	4.90E-29	2.15E-26	60
LUZP1	4.30E-11	1.065	5.60E-29	2.42E-26	61
PDPN	8.30E-13	2.975	5.74E-29	2.44E-26	62
DDAH1	1.73E-06	-1.519	7.92E-29	3.26E-26	64
PRR15L	1.43E-05	-3.797	9.71E-29	3.88E-26	66
SLC16A1	1.43E-10	2.084	1.03E-28	4.01E-26	67
SLC26A6	1.78E-12	1.454	1.03E-28	4.01E-26	68
SYT15	7.53E-12	-2.211	1.23E-28	4.64E-26	70
CTSC	2.33E-11	1.556	1.55E-28	5.74E-26	71
SPATA6	5.81E-13	-2.282	1.96E-28	7.08E-26	73
COL12A1	2.10E-13	2.079	2.00E-28	7.09E-26	74
SP110	9.98E-15	1.871	2.29E-28	7.85E-26	77
LPCAT1	7.66E-14	1.654	2.34E-28	7.89E-26	78
ADAM12	4.18E-15	4.013	3.83E-28	1.28E-25	79
ARPC1B	2.00E-13	1.608	4.16E-28	1.35E-25	81
COL4A6	1.20E-14	3.422	5.68E-28	1.83E-25	82
USB1	1.20E-14	1.730	5.94E-28	1.89E-25	83
PLEK2	1.43E-07	2.341	6.60E-28	2.05E-25	85
FAP	4.62E-11	2.567	1.54E-27	4.67E-25	87
XPR1	7.03E-14	1.388	1.69E-27	5.06E-25	88
SYTL4	2.38E-11	-2.080	2.11E-27	6.24E-25	89
FLRT3	9.25E-10	3.085	2.24E-27	6.57E-25	90
C6orf141	3.18E-08	2.248	6.13E-27	1.76E-24	92
MYO1B	1.89E-13	2.169	7.32E-27	2.07E-24	93
LAMA3	1.65E-09	2.834	7.92E-27	2.22E-24	94
GALNT12	6.36E-10	-3.511	8.93E-27	2.43E-24	97
BEX4	8.51E-14	-2.354	9.94E-27	2.67E-24	98
AGFG2	4.78E-07	-2.323	1.11E-26	2.96E-24	99
PLA2G7	1.52E-11	3.059	1.14E-26	3.01E-24	100
SLC26A2	7.87E-07	-2.437	1.36E-26	3.56E-24	101
MSN	4.52E-13	1.474	1.75E-26	4.49E-24	103
VAV2	3.33E-13	1.981	1.89E-26	4.78E-24	104
MET	3.96E-11	1.611	2.68E-26	6.68E-24	105

ST3GAL4	1.09E-04	-1.201	2.69E-26	6.68E-24	106
IFI30	1.76E-10	1.825	3.55E-26	8.66E-24	108
SNAI2	8.66E-12	2.046	3.77E-26	9.13E-24	109
LIMA1	3.83E-13	1.484	3.92E-26	9.39E-24	110
CYP27B1	2.30E-11	3.596	4.25E-26	1.00E-23	112
BOC	7.16E-13	-1.968	4.38E-26	1.02E-23	113
SNX10	4.77E-11	1.973	1.22E-25	2.81E-23	114
RALGPS1	7.93E-13	-1.631	1.72E-25	3.95E-23	115
SLC20A1	2.58E-10	1.357	2.31E-25	5.16E-23	118
FAM49B	4.23E-13	1.158	2.44E-25	5.40E-23	119
C12orf75	1.44E-11	2.399	2.46E-25	5.40E-23	120
C14orf132	4.60E-11	-1.822	2.56E-25	5.52E-23	122
CTTNBP2	1.48E-15	-2.837	2.81E-25	5.96E-23	124
TGFBR3	4.82E-14	-2.495	2.95E-25	6.23E-23	125
AIM2	1.04E-07	1.802	3.30E-25	6.91E-23	126
HTR7	6.29E-14	3.585	3.94E-25	8.18E-23	127
EMP1	2.36E-06	-1.911	4.04E-25	8.32E-23	128
FBLIM1	2.52E-10	1.758	4.70E-25	9.61E-23	129
SLC38A7	3.04E-13	1.048	5.31E-25	1.08E-22	130
RBM20	1.70E-10	-3.170	6.47E-25	1.27E-22	134
ZNF132	2.17E-12	-1.732	1.12E-24	2.15E-22	137
ANGPT2	6.70E-09	1.567	1.26E-24	2.41E-22	138
DNMT3B	7.77E-11	2.473	1.34E-24	2.54E-22	139
PLEKHA6	9.18E-09	-1.953	1.72E-24	3.23E-22	140
DFNA5	2.10E-15	2.714	1.86E-24	3.47E-22	141
PRNP	1.40E-10	1.480	1.87E-24	3.47E-22	142
SFXN3	5.05E-10	1.178	2.00E-24	3.69E-22	143
PHLDB2	1.64E-11	1.359	2.03E-24	3.71E-22	144
BST2	3.21E-13	2.590	2.29E-24	4.16E-22	145
AR	1.93E-11	-2.955	2.43E-24	4.36E-22	147
ABL2	4.33E-10	1.222	2.57E-24	4.54E-22	149
CAPN5	2.43E-07	-1.695	2.61E-24	4.59E-22	150
PSMB2	4.89E-14	1.175	3.55E-24	6.19E-22	151
C1QTNF7	7.95E-11	-1.405	3.75E-24	6.50E-22	152
FSTL3	2.13E-10	2.037	4.19E-24	7.12E-22	155
CD276	1.32E-14	1.799	4.52E-24	7.60E-22	157
ADAMTS12	1.37E-12	3.095	8.00E-24	1.29E-21	163
MAGIX	1.79E-10	-1.906	1.07E-23	1.71E-21	165
GPR39	2.38E-07	2.264	1.46E-23	2.28E-21	169
LIMK1	9.21E-13	1.223	1.81E-23	2.78E-21	172
HLF	2.10E-15	-3.327	2.61E-23	3.87E-21	177
FNDC4	4.53E-07	-1.633	2.61E-23	3.87E-21	178

GULP1	4.66E-15	-2.594	2.96E-23	4.36E-21	179
FSCN1	2.43E-10	2.186	4.72E-23	6.77E-21	184
NAGS	7.24E-10	1.949	4.87E-23	6.94E-21	185
PPIL6	5.00E-09	-1.754	4.90E-23	6.95E-21	186
CCT6B	6.33E-11	-1.514	5.81E-23	8.10E-21	189
DDX60	4.34E-12	2.263	6.98E-23	9.63E-21	191
FCGR3A	1.80E-09	2.339	7.07E-23	9.66E-21	193
LRFN4	2.09E-11	1.646	7.38E-23	1.00E-20	194
C4orf48	9.14E-13	2.119	8.69E-23	1.17E-20	196
CLEC3B	7.96E-14	-3.316	9.32E-23	1.24E-20	198
ZNF471	1.26E-12	-2.556	1.09E-22	1.44E-20	200
OAS2	9.87E-12	2.353	1.10E-22	1.44E-20	201
GALNT5	1.67E-04	-2.668	1.11E-22	1.44E-20	202
ALDH6A1	1.62E-11	-2.151	1.58E-22	2.03E-20	205
PTHLH	4.95E-11	3.666	1.80E-22	2.30E-20	207
N4BP3	1.67E-07	-1.413	1.93E-22	2.44E-20	209
IFI6	7.53E-11	2.072	2.04E-22	2.57E-20	210
PLOD1	7.54E-13	1.251	2.17E-22	2.71E-20	211
NFIX	2.14E-14	-2.103	2.29E-22	2.85E-20	212
KLF7	7.76E-11	1.375	2.37E-22	2.93E-20	213
PITX2	7.00E-09	-2.606	3.46E-22	4.21E-20	217
IRS1	9.47E-10	1.551	3.48E-22	4.21E-20	218
SMIM3	1.67E-10	1.456	4.08E-22	4.87E-20	221
FOXN2	1.12E-10	1.039	4.54E-22	5.32E-20	225
LTBP1	7.55E-12	1.919	4.71E-22	5.50E-20	226
FMNL2	5.88E-12	1.385	5.09E-22	5.91E-20	227
ITGA6	3.50E-11	1.906	5.13E-22	5.93E-20	228
PBX1	3.98E-14	-2.507	6.14E-22	7.00E-20	231
STX1A	2.41E-12	1.710	6.26E-22	7.11E-20	232
FEZ1	1.10E-12	2.216	6.50E-22	7.36E-20	233
PLXNA1	1.09E-11	1.364	7.90E-22	8.83E-20	236
ITGAV	2.64E-06	1.054	1.08E-21	1.18E-19	242
SHC1	1.50E-12	1.267	1.15E-21	1.25E-19	244
PARP14	1.55E-12	1.741	1.18E-21	1.27E-19	245
TMC7	1.16E-12	2.587	1.22E-21	1.31E-19	246
GUCY2C	1.00E-09	-1.230	1.24E-21	1.32E-19	247
RMND5B	5.00E-09	-1.130	1.43E-21	1.51E-19	250
KIRREL	1.87E-12	1.383	1.61E-21	1.69E-19	252
PTGFRN	1.40E-09	1.421	1.85E-21	1.93E-19	253
CCRL2	1.31E-06	1.054	1.90E-21	1.98E-19	254
STAT1	9.37E-15	1.965	1.98E-21	2.05E-19	255
WDR66	1.55E-11	3.156	2.06E-21	2.12E-19	256

SLC2A9	1.15E-09	1.810	2.17E-21	2.22E-19	257
BCKDHB	1.75E-10	-1.426	2.53E-21	2.59E-19	258
GLIPR1	6.56E-12	1.624	2.79E-21	2.83E-19	260
CHST11	1.44E-12	1.922	3.15E-21	3.17E-19	262
MB21D1	2.00E-13	2.147	3.25E-21	3.25E-19	264
MYO10	2.78E-10	1.472	3.69E-21	3.67E-19	265
AMIGO2	6.35E-09	2.228	4.07E-21	4.03E-19	266
RRAS2	1.56E-11	1.294	4.25E-21	4.20E-19	267
NFIA	5.56E-16	-1.393	4.29E-21	4.22E-19	268
MT2A	1.40E-08	1.288	4.39E-21	4.30E-19	269
VEGFC	1.94E-09	2.412	4.71E-21	4.60E-19	270
ZFP28	2.20E-12	-1.944	4.87E-21	4.74E-19	271
BNC1	6.11E-06	1.922	4.99E-21	4.84E-19	272
UPK3B	1.32E-03	-2.339	6.52E-21	6.19E-19	278
ADA	4.14E-13	1.767	7.34E-21	6.89E-19	281
SPRY4	7.41E-12	2.091	8.01E-21	7.44E-19	284
ENAH	7.50E-11	1.587	8.67E-21	7.97E-19	287
SLC25A21	1.01E-12	-2.803	9.81E-21	8.95E-19	289
IRF7	3.15E-13	2.102	1.02E-20	9.30E-19	290
MMP14	2.95E-11	1.626	1.04E-20	9.41E-19	292
GALNT18	3.96E-11	1.440	1.11E-20	9.88E-19	296
BATF2	2.74E-09	3.309	1.29E-20	1.14E-18	300
PPARGC1A	7.48E-10	-3.398	1.37E-20	1.20E-18	301
ALDH3A2	7.94E-10	-1.241	1.39E-20	1.21E-18	302
YEATS2	1.97E-10	1.009	1.44E-20	1.25E-18	303
RAG1	2.13E-07	2.742	1.61E-20	1.40E-18	304
GBP1	2.26E-11	2.264	1.79E-20	1.54E-18	307
GPD1L	6.89E-11	-2.508	1.89E-20	1.60E-18	310
COL4A5	1.01E-11	2.216	1.92E-20	1.63E-18	311
PLEKHG6	1.25E-09	-2.390	2.58E-20	2.15E-18	317
VSIG10	5.17E-07	-1.424	2.78E-20	2.29E-18	319
WDR54	5.68E-12	1.904	2.91E-20	2.39E-18	320
COL4A2	6.32E-14	2.085	3.00E-20	2.46E-18	321
GNA12	1.06E-14	1.183	3.02E-20	2.48E-18	322
NREP	2.05E-09	1.405	3.23E-20	2.64E-18	323
LYPD1	9.37E-15	3.523	3.47E-20	2.83E-18	324
IKZF2	4.95E-11	-1.720	3.54E-20	2.87E-18	325
SLC3A2	2.33E-11	1.235	3.59E-20	2.90E-18	326
ZNF704	2.66E-08	-1.881	4.56E-20	3.62E-18	332
IFITM3	1.70E-11	1.504	5.28E-20	4.16E-18	334
ISG15	3.36E-13	3.152	5.29E-20	4.16E-18	335
KYNU	3.73E-11	2.641	5.68E-20	4.46E-18	336

EIF2AK2	1.82E-12	1.672	5.81E-20	4.53E-18	338
RIPK2	4.81E-10	1.178	5.88E-20	4.57E-18	339
CGNL1	4.72E-12	-3.349	6.68E-20	5.12E-18	344
EPB41L4A	5.89E-12	-1.639	6.74E-20	5.15E-18	345
IER5L	9.10E-11	1.432	7.02E-20	5.33E-18	347
PGD	2.04E-04	-1.222	7.08E-20	5.37E-18	348
HERC5	1.27E-08	1.962	7.29E-20	5.51E-18	349
ADAMTS2	8.51E-14	2.703	7.54E-20	5.68E-18	350
CDON	2.17E-12	-1.792	8.06E-20	6.00E-18	354
EVA1A	4.06E-09	2.893	8.15E-20	6.04E-18	355
GPX3	3.96E-11	-2.466	8.16E-20	6.04E-18	356
IGF2BP3	1.31E-03	2.158	9.58E-20	7.03E-18	359
RAC2	1.64E-09	1.594	9.62E-20	7.05E-18	360
LCA5	8.82E-08	-1.156	1.05E-19	7.63E-18	362
TNS4	1.71E-05	1.449	1.05E-19	7.64E-18	363
SLMO1	8.66E-11	1.804	1.09E-19	7.84E-18	365
PLAC9	3.41E-13	-2.648	1.12E-19	8.03E-18	368
KLB	2.70E-13	-1.734	1.49E-19	1.05E-17	372
CPAMD8	1.60E-10	-3.285	1.74E-19	1.23E-17	374
ANKRD6	4.66E-12	-1.938	1.78E-19	1.25E-17	376
NT5E	1.12E-07	1.860	1.89E-19	1.32E-17	377
AUNIP	3.69E-11	2.147	1.92E-19	1.34E-17	378
SLC37A1	1.02E-08	-1.498	2.34E-19	1.63E-17	379
CTHRC1	2.56E-11	2.600	3.15E-19	2.15E-17	386
SORBS1	5.10E-11	-2.856	3.22E-19	2.20E-17	387
SEMA3C	1.30E-09	1.387	4.00E-19	2.71E-17	390
CDC25B	4.10E-13	2.027	4.04E-19	2.72E-17	391
B4GALT6	3.04E-08	-1.728	4.09E-19	2.74E-17	393
BCAS1	1.63E-06	-3.042	4.40E-19	2.93E-17	396
TRIM21	8.08E-13	1.472	4.54E-19	3.01E-17	398
SH3BGRL2	4.18E-15	-3.599	4.56E-19	3.01E-17	399
RNF180	3.78E-12	-2.240	4.67E-19	3.07E-17	401
CDH3	9.36E-08	2.733	4.69E-19	3.07E-17	402
C19orf66	1.01E-12	1.461	5.75E-19	3.75E-17	404
EPSTI1	6.85E-12	2.617	6.10E-19	3.96E-17	406
TPPP	1.08E-10	-2.254	7.76E-19	4.98E-17	411
GPR153	5.70E-09	1.814	8.18E-19	5.22E-17	413
OSR1	1.21E-11	-3.545	8.85E-19	5.60E-17	417
OSMR	2.06E-09	1.180	9.68E-19	6.09E-17	419
FN1	5.00E-09	2.074	9.90E-19	6.21E-17	420
ODC1	6.51E-05	1.097	1.13E-18	7.05E-17	422
NXPH3	9.35E-13	-2.361	1.25E-18	7.76E-17	424

ABCA10	2.17E-12	-2.200	1.26E-18	7.78E-17	426
HOMER3	1.91E-11	1.619	1.35E-18	8.30E-17	428
USP18	7.00E-09	2.159	1.45E-18	8.86E-17	430
SORBS2	2.32E-08	-2.197	1.46E-18	8.89E-17	432
FAM174B	2.91E-09	-2.499	1.47E-18	8.95E-17	433
SP140L	2.12E-11	1.221	1.53E-18	9.26E-17	436
TNS1	3.20E-10	-1.990	1.55E-18	9.37E-17	437
UBE2L6	5.50E-13	1.871	1.70E-18	1.03E-16	438
ARHGEF26	1.06E-16	-3.141	1.82E-18	1.09E-16	440
WARS	2.03E-11	2.494	1.86E-18	1.11E-16	441
QRICH2	7.93E-13	-1.679	1.86E-18	1.11E-16	442
AURKA	1.97E-12	2.051	1.93E-18	1.15E-16	443
NRG1	1.68E-06	1.954	2.01E-18	1.19E-16	445
FBXO6	7.37E-11	1.341	2.06E-18	1.22E-16	446
GATM	7.66E-14	-2.919	2.38E-18	1.40E-16	449
IFITM1	8.08E-09	1.361	2.42E-18	1.41E-16	451
LIFR	6.85E-12	-2.750	2.69E-18	1.56E-16	455
CDK6	1.11E-09	1.426	2.71E-18	1.56E-16	457
IFI44	6.85E-12	2.252	2.73E-18	1.57E-16	458
AGTRAP	1.89E-13	1.229	3.06E-18	1.75E-16	462
TSPAN6	2.11E-05	-1.137	3.12E-18	1.77E-16	463
COL5A2	4.10E-13	2.535	3.17E-18	1.80E-16	464
ZNF844	4.32E-13	-2.215	3.36E-18	1.90E-16	465
MYO5A	3.20E-11	1.568	3.77E-18	2.13E-16	467
TENM2	1.29E-08	2.488	4.06E-18	2.28E-16	469
STIL	1.42E-09	1.668	4.19E-18	2.34E-16	471
FAM89A	7.66E-08	1.505	4.34E-18	2.42E-16	473
CPEB1	2.47E-10	-2.541	4.93E-18	2.73E-16	477
ZNF229	7.09E-11	-2.409	5.74E-18	3.15E-16	480
PARP12	4.82E-16	2.146	6.61E-18	3.62E-16	481
KIAA1211L	4.51E-09	-1.862	7.00E-18	3.83E-16	482
IL34	7.93E-13	-2.445	7.06E-18	3.85E-16	483
PTN	6.51E-13	-3.045	8.00E-18	4.32E-16	488
MARC2	3.26E-09	-2.164	8.19E-18	4.40E-16	491
AJUBA	1.63E-06	1.510	8.38E-18	4.48E-16	493
IDO1	6.54E-11	3.763	9.81E-18	5.22E-16	495
IFI35	2.56E-11	2.124	1.11E-17	5.82E-16	501
PGF	2.28E-08	1.835	1.14E-17	5.99E-16	502
OCIAD2	7.68E-11	1.960	1.18E-17	6.20E-16	503
IFIT1	1.76E-09	1.683	1.19E-17	6.20E-16	505
ZNFX1	4.98E-11	1.206	1.22E-17	6.35E-16	506
GAS7	5.94E-09	-1.439	1.32E-17	6.80E-16	510

LONRF1	2.14E-14	-1.756	1.35E-17	6.96E-16	512
RSAD2	4.47E-11	3.083	1.47E-17	7.48E-16	517
KIAA1456	3.67E-11	-2.303	1.91E-17	9.56E-16	526
NR3C2	8.66E-12	-3.339	1.96E-17	9.82E-16	527
MMP12	3.70E-13	4.850	2.05E-17	1.02E-15	528
MPZ	1.97E-12	-3.446	2.07E-17	1.03E-15	529
ARRDC4	4.71E-05	1.656	2.49E-17	1.23E-15	535
PTPRK	7.04E-12	1.564	2.53E-17	1.24E-15	536
ZSCAN18	3.71E-14	-2.333	2.59E-17	1.27E-15	537
MMP9	7.04E-12	3.337	2.60E-17	1.27E-15	538
ITPR3	1.19E-09	1.331	2.77E-17	1.35E-15	542
DDIT4	9.32E-08	1.446	2.82E-17	1.37E-15	544
ARSG	2.17E-12	-1.555	2.86E-17	1.38E-15	546
LTBP4	5.53E-12	-2.027	4.01E-17	1.90E-15	558
DEPTOR	1.36E-11	-3.468	4.17E-17	1.96E-15	562
KNSTRN	1.49E-10	1.336	4.27E-17	2.00E-15	563
PMEL	4.38E-07	-4.208	4.91E-17	2.27E-15	570
AFAP1L2	2.35E-08	1.580	5.08E-17	2.34E-15	572
ZNF281	6.32E-09	1.025	5.14E-17	2.37E-15	573
ADAM33	1.47E-11	-2.073	5.24E-17	2.40E-15	574
TMEM150C	2.43E-11	-2.257	5.75E-17	2.60E-15	582
APOL1	6.08E-12	2.581	6.39E-17	2.89E-15	584
PAX9	4.11E-09	-2.547	7.48E-17	3.35E-15	589
STARD4	3.33E-08	1.390	7.92E-17	3.53E-15	591
SELENBP1	2.33E-11	-3.381	8.44E-17	3.73E-15	597
NOXA1	2.08E-08	-1.586	9.84E-17	4.28E-15	606
SIRPA	7.34E-12	1.327	9.90E-17	4.29E-15	608
IGF2BP2	4.09E-11	2.570	1.05E-16	4.54E-15	613
UBASH3B	2.06E-08	1.410	1.14E-16	4.90E-15	615
GSN	3.49E-09	-1.372	1.17E-16	4.99E-15	616
LRRC8D	2.37E-09	1.290	1.19E-16	5.08E-15	617
PLLP	2.85E-12	-2.306	1.21E-16	5.17E-15	618
MCF2L	7.10E-12	-2.114	1.25E-16	5.30E-15	619
CDC6	6.11E-11	2.312	1.26E-16	5.35E-15	620
PLAGL1	2.97E-12	-2.005	1.27E-16	5.37E-15	621
GOLGA7B	8.00E-08	2.382	1.38E-16	5.80E-15	626
SMS	3.25E-10	1.134	1.49E-16	6.24E-15	631
F2RL1	4.86E-05	1.266	1.53E-16	6.36E-15	633
EHD3	3.46E-03	-1.008	1.61E-16	6.67E-15	635
MTBP	4.70E-10	1.679	1.69E-16	7.01E-15	636
CCNE1	5.79E-11	1.580	1.83E-16	7.57E-15	638
FMO2	1.56E-12	-3.701	1.96E-16	8.09E-15	639

ADAMTSL3	2.05E-11	-2.849	2.02E-16	8.30E-15	640
SLC39A6	3.15E-06	1.034	2.11E-16	8.65E-15	642
SH2D2A	1.44E-11	2.467	2.17E-16	8.89E-15	643
CD274	1.59E-07	2.056	2.48E-16	1.01E-14	648
APOL2	9.34E-09	1.284	2.58E-16	1.05E-14	649
SALL2	8.69E-11	-1.476	2.66E-16	1.08E-14	651
TNFRSF10B	1.79E-07	1.009	2.73E-16	1.10E-14	652
STAT2	5.29E-13	1.431	2.75E-16	1.11E-14	653
SLC15A3	7.83E-11	1.999	2.77E-16	1.12E-14	654
MARVELD1	1.16E-10	1.409	2.86E-16	1.15E-14	656
SUSD5	1.75E-09	-1.850	2.96E-16	1.19E-14	657
ADAMTS6	8.12E-07	1.580	3.07E-16	1.23E-14	660
TENM3	2.88E-07	1.708	3.08E-16	1.23E-14	661
NCS1	5.18E-09	1.218	3.14E-16	1.25E-14	662
IFIH1	1.76E-11	1.876	3.53E-16	1.40E-14	666
PCDH7	5.00E-07	1.516	3.72E-16	1.47E-14	668
TMEM132A	1.62E-11	2.228	3.75E-16	1.48E-14	669
STON2	9.37E-08	1.432	3.87E-16	1.52E-14	671
CASP7	2.06E-10	1.086	3.97E-16	1.55E-14	675
PARK2	4.18E-12	-2.798	4.03E-16	1.57E-14	676
PLIN4	3.70E-13	-4.147	4.28E-16	1.66E-14	678
LDOC1	6.34E-10	-2.887	4.45E-16	1.72E-14	681
MFSD10	2.59E-12	1.148	4.48E-16	1.73E-14	682
OAS3	1.05E-12	2.217	4.53E-16	1.74E-14	684
ADAMTSL2	2.53E-08	1.149	4.96E-16	1.90E-14	688
CDCA5	2.43E-10	2.466	5.14E-16	1.96E-14	691
TRPM2	1.08E-09	2.058	5.67E-16	2.13E-14	701
IFIT3	7.53E-11	2.500	5.83E-16	2.19E-14	702
PARP9	4.14E-13	1.545	5.99E-16	2.25E-14	703
IL1RAP	7.22E-07	1.224	6.27E-16	2.34E-14	706
MYO5C	2.94E-08	-2.641	6.47E-16	2.40E-14	710
C16orf74	5.83E-08	1.794	6.56E-16	2.43E-14	711
TRIM22	7.45E-10	1.346	6.77E-16	2.51E-14	712
SERPINE2	4.01E-08	2.293	6.93E-16	2.56E-14	713
BCAT1	5.95E-08	1.586	7.05E-16	2.60E-14	714
SYNGR1	2.17E-12	-2.320	7.09E-16	2.61E-14	716
FBN2	1.66E-08	2.717	7.80E-16	2.86E-14	719
IL12RB2	3.00E-07	2.961	7.82E-16	2.86E-14	720
KIAA1524	2.29E-11	1.752	7.97E-16	2.92E-14	721
METTL21A	2.07E-10	1.123	8.13E-16	2.97E-14	723
PPFIA1	5.66E-05	1.035	8.54E-16	3.10E-14	726
BMP1	3.13E-11	1.887	8.93E-16	3.22E-14	731

MAML3	4.62E-11	-1.600	9.54E-16	3.43E-14	733
TUB	7.50E-11	-1.943	9.81E-16	3.52E-14	735
OASL	4.42E-11	2.653	9.94E-16	3.56E-14	737
SOX5	7.34E-12	-1.878	1.02E-15	3.62E-14	739
CD300LF	1.79E-06	1.354	1.02E-15	3.64E-14	740
CMPK2	2.72E-10	2.502	1.12E-15	3.97E-14	743
FJX1	3.74E-10	1.978	1.13E-15	4.00E-14	744
MAGI1	4.93E-13	-1.481	1.13E-15	4.00E-14	745
ACP5	1.95E-08	1.446	1.19E-15	4.21E-14	748
LTF	1.47E-05	-3.436	1.21E-15	4.26E-14	749
HAAO	1.74E-11	-1.551	1.22E-15	4.30E-14	750
PARD3B	7.93E-13	-1.607	1.25E-15	4.39E-14	751
IFIT2	1.13E-05	1.804	1.34E-15	4.67E-14	754
SLC2A1	5.23E-06	1.593	1.46E-15	5.08E-14	760
FAM26F	1.41E-07	2.001	1.60E-15	5.52E-14	763
PDP1	1.48E-09	1.035	1.61E-15	5.57E-14	764
CKAP2	7.86E-11	1.414	1.64E-15	5.65E-14	766
ETS1	3.85E-09	1.178	1.78E-15	6.10E-14	768
EPHX2	8.14E-11	-1.823	1.80E-15	6.16E-14	769
PFN2	1.05E-07	1.135	1.91E-15	6.50E-14	774
PDCD1LG2	4.76E-08	2.053	1.94E-15	6.58E-14	776
NGF	1.85E-07	2.094	1.95E-15	6.63E-14	777
NEBL	8.59E-07	-1.478	2.02E-15	6.84E-14	779
ZNF880	1.43E-10	-2.085	2.10E-15	7.05E-14	784
PCCA	7.78E-11	-1.522	2.23E-15	7.44E-14	790
NRIP3	1.27E-08	2.146	2.35E-15	7.83E-14	793
APBA2	2.68E-11	2.443	2.47E-15	8.16E-14	797
DTX3L	2.34E-13	1.400	2.48E-15	8.18E-14	798
ICOS	1.20E-09	2.489	2.53E-15	8.34E-14	801
CHPT1	6.05E-09	-2.435	2.64E-15	8.66E-14	805
CEP55	2.20E-10	2.407	2.94E-15	9.58E-14	810
LRRC15	3.67E-10	2.488	3.00E-15	9.71E-14	813
CLDN7	1.76E-03	-1.869	3.06E-15	9.89E-14	815
PMEPA1	4.58E-09	1.535	3.07E-15	9.93E-14	816
EME1	4.79E-10	1.827	3.15E-15	1.02E-13	817
CCND2	6.54E-04	1.086	3.23E-15	1.04E-13	819
TMEM184B	2.15E-10	1.035	3.28E-15	1.06E-13	820
SASH1	4.34E-09	-1.169	3.43E-15	1.10E-13	824
TNFRSF11A	8.22E-08	-1.721	3.54E-15	1.13E-13	827
ECT2	1.04E-07	1.240	3.62E-15	1.15E-13	828
ITGB4	4.76E-09	1.620	3.71E-15	1.18E-13	830
PAMR1	1.10E-07	-1.482	3.98E-15	1.26E-13	834

RGS4	9.86E-09	2.881	4.02E-15	1.27E-13	835
NMI	3.69E-11	1.483	4.08E-15	1.29E-13	837
MEFV	9.55E-07	1.309	4.21E-15	1.32E-13	838
EYA2	1.10E-09	-2.186	4.57E-15	1.43E-13	844
CSPG4	3.42E-07	1.552	4.62E-15	1.44E-13	846
PML	1.10E-12	1.591	5.00E-15	1.55E-13	851
LY6E	4.37E-10	1.816	5.04E-15	1.56E-13	852
SLC7A8	4.17E-09	1.845	5.38E-15	1.66E-13	856
ACOX2	2.60E-09	-1.763	5.48E-15	1.68E-13	857
TCOF1	4.00E-11	1.064	5.48E-15	1.68E-13	858
CDCA4	1.50E-08	1.455	5.49E-15	1.68E-13	859
ZNF568	1.87E-11	-1.845	5.83E-15	1.79E-13	861
DCLK1	9.06E-09	-1.892	5.86E-15	1.79E-13	862
ISM1	3.92E-07	-1.193	6.55E-15	1.98E-13	871
DPT	4.52E-13	-3.210	6.76E-15	2.04E-13	873
HPDL	8.86E-10	-1.859	6.95E-15	2.10E-13	874
HSD17B6	1.79E-09	2.225	7.15E-15	2.15E-13	875
INPP4B	2.62E-08	1.120	7.47E-15	2.24E-13	878
CXCL9	1.10E-09	3.019	7.79E-15	2.33E-13	881
CYP26B1	3.91E-06	1.000	7.90E-15	2.36E-13	882
FAIM2	1.49E-10	-2.316	8.47E-15	2.52E-13	886
C1orf115	3.10E-09	-2.562	8.65E-15	2.56E-13	890
KMO	1.09E-05	1.261	8.82E-15	2.61E-13	892
CXCR2	3.70E-07	-1.821	9.82E-15	2.88E-13	898
CLDN11	6.63E-10	-2.504	9.86E-15	2.89E-13	899
CNTNAP3B	2.15E-10	-1.429	1.09E-14	3.17E-13	905
IL2RA	7.21E-08	1.990	1.13E-14	3.29E-13	906
C10orf55	6.11E-11	1.776	1.34E-14	3.88E-13	914
CHST1	8.83E-07	1.827	1.47E-14	4.23E-13	917
TCF3	1.06E-10	1.000	1.50E-14	4.29E-13	920
SLC2A6	8.82E-10	1.636	1.51E-14	4.33E-13	922
CDC20	2.69E-09	2.475	1.55E-14	4.43E-13	925
SYDE2	4.63E-08	-1.635	1.56E-14	4.44E-13	926
RFC4	1.79E-10	1.300	1.70E-14	4.82E-13	931
NEFL	2.31E-08	4.086	1.73E-14	4.90E-13	932
DRAM1	1.15E-08	1.470	1.80E-14	5.05E-13	937
GLS2	9.51E-09	-1.668	1.94E-14	5.44E-13	941
BARX2	8.63E-08	-2.164	2.01E-14	5.59E-13	947
LMF1	1.78E-09	-1.047	2.02E-14	5.61E-13	949
COL22A1	7.79E-07	4.143	2.04E-14	5.67E-13	950
GNLY	7.58E-09	2.772	2.05E-14	5.70E-13	951
KIF14	9.47E-10	2.487	2.17E-14	6.01E-13	952

LILRB4	2.51E-06	1.644	2.22E-14	6.12E-13	955
APOC1	6.48E-07	1.876	2.34E-14	6.41E-13	960
TCP11L2	3.61E-10	-1.399	2.73E-14	7.43E-13	969
HSPB8	2.77E-07	-1.877	2.73E-14	7.43E-13	971
SNED1	4.83E-11	-1.796	3.06E-14	8.25E-13	978
PKIB	1.11E-06	-1.859	3.08E-14	8.30E-13	979
ECHDC2	3.27E-11	-1.870	3.18E-14	8.55E-13	980
TGM2	8.32E-05	1.870	3.29E-14	8.80E-13	984
CD80	1.67E-11	2.929	3.45E-14	9.20E-13	989
ITGA5	8.93E-08	1.492	3.47E-14	9.23E-13	990
CFD	4.68E-12	-3.052	3.55E-14	9.44E-13	992
KLF15	3.13E-11	-4.367	3.58E-14	9.49E-13	995
DNAH17	5.78E-10	3.036	3.69E-14	9.74E-13	1000
SIGLEC7	4.92E-06	1.379	3.92E-14	1.03E-12	1006
MELK	6.52E-09	2.304	3.94E-14	1.03E-12	1009
MLANA	1.32E-07	-2.251	3.99E-14	1.04E-12	1010
SLC44A3	1.66E-04	-1.593	4.21E-14	1.10E-12	1012
NRP2	4.25E-08	1.180	4.21E-14	1.10E-12	1013
TRAM2	8.48E-10	1.435	4.24E-14	1.10E-12	1015
LAP3	4.67E-09	1.236	4.47E-14	1.15E-12	1022
BORA	1.62E-10	1.288	4.59E-14	1.18E-12	1024
RNF213	1.47E-11	1.387	4.80E-14	1.23E-12	1027
FAM107A	4.25E-12	-2.949	4.84E-14	1.24E-12	1028
PRELP	4.94E-11	-2.484	4.85E-14	1.24E-12	1029
SYT8	4.53E-06	-3.035	4.89E-14	1.25E-12	1031
TDRD6	5.65E-11	2.359	5.11E-14	1.30E-12	1037
TNFRSF19	6.08E-07	-1.828	5.11E-14	1.30E-12	1038
CCDC109B	9.16E-09	1.237	5.16E-14	1.31E-12	1039
ARHGAP20	1.74E-14	-2.110	5.33E-14	1.35E-12	1043
RUVBL1	7.39E-11	1.046	5.34E-14	1.35E-12	1044
FCER1G	1.68E-06	1.417	5.61E-14	1.41E-12	1049
MDFI	6.50E-09	1.508	5.91E-14	1.48E-12	1053
PLSCR1	2.21E-11	1.386	6.40E-14	1.59E-12	1059
NDNF	1.85E-12	-2.934	6.42E-14	1.60E-12	1060
MANSC1	3.86E-09	-2.147	6.53E-14	1.62E-12	1061
SMTN	1.69E-06	1.108	6.86E-14	1.70E-12	1063
PXDN	3.21E-11	2.144	7.13E-14	1.77E-12	1064
DDX58	2.91E-09	1.181	7.21E-14	1.78E-12	1065
AGRN	1.34E-11	1.819	7.45E-14	1.84E-12	1067
CHADL	1.10E-12	-2.191	7.67E-14	1.89E-12	1069
OSBPL3	1.24E-08	1.242	8.44E-14	2.06E-12	1080
LAMP3	2.21E-07	2.076	8.68E-14	2.12E-12	1082

KRT17	1.31E-07	2.390	8.95E-14	2.18E-12	1083
AMY2B	1.29E-08	-1.045	9.84E-14	2.38E-12	1088
NID1	5.77E-09	1.669	1.02E-13	2.45E-12	1092
EFNB1	7.25E-10	1.611	1.02E-13	2.45E-12	1093
POP1	5.03E-08	1.221	1.08E-13	2.58E-12	1099
CREB3L4	2.59E-05	-2.773	1.23E-13	2.92E-12	1109
ELF4	4.41E-09	1.190	1.24E-13	2.93E-12	1110
SAMD5	4.12E-05	-2.104	1.28E-13	3.02E-12	1113
GKAP1	5.10E-09	-2.405	1.28E-13	3.02E-12	1114
SCNN1D	5.55E-05	2.726	1.29E-13	3.06E-12	1116
HENMT1	2.20E-10	1.586	1.32E-13	3.12E-12	1117
CLYBL	8.72E-11	-1.721	1.32E-13	3.12E-12	1118
DOPEY2	2.31E-07	-1.139	1.33E-13	3.12E-12	1119
NEIL1	4.10E-11	-1.921	1.38E-13	3.25E-12	1121
ADCY5	5.39E-12	-2.454	1.50E-13	3.50E-12	1128
MATN3	4.53E-09	2.253	1.54E-13	3.58E-12	1134
CDC45	1.70E-10	1.986	1.55E-13	3.60E-12	1135
DLG2	3.87E-13	-2.642	1.57E-13	3.64E-12	1137
KALRN	1.08E-05	-1.183	1.58E-13	3.66E-12	1138
PGR	7.49E-08	-2.342	1.59E-13	3.68E-12	1139
GLTSCR2	1.34E-13	-1.059	1.66E-13	3.83E-12	1142
FEN1	4.62E-11	1.494	1.68E-13	3.87E-12	1144
COL6A3	1.76E-09	1.534	1.87E-13	4.28E-12	1150
ZNF677	7.96E-11	-2.008	1.89E-13	4.32E-12	1152
PNPLA7	8.15E-11	-3.150	1.96E-13	4.48E-12	1155
CTPS1	8.47E-09	1.002	1.98E-13	4.51E-12	1156
MFAP4	9.06E-12	-2.793	2.05E-13	4.66E-12	1157
SPARC	5.07E-09	1.147	2.05E-13	4.67E-12	1158
SYP	9.47E-10	-1.354	2.09E-13	4.73E-12	1162
TMEM220	5.26E-10	-1.776	2.11E-13	4.77E-12	1164
MPC1	1.94E-08	-1.492	2.14E-13	4.83E-12	1166
PRX	5.57E-10	-2.185	2.15E-13	4.85E-12	1167
FZD7	2.76E-07	-1.087	2.29E-13	5.16E-12	1169
ALDH3B1	6.42E-06	-1.159	2.37E-13	5.33E-12	1174
KIF2C	2.38E-10	2.209	2.38E-13	5.34E-12	1175
IL2RB	1.10E-07	1.646	2.39E-13	5.35E-12	1176
FLRT2	8.65E-07	1.687	2.43E-13	5.45E-12	1177
BEND6	1.69E-09	1.901	2.49E-13	5.56E-12	1181
NPNT	1.03E-03	1.363	2.56E-13	5.70E-12	1183
ACADSB	3.58E-10	-2.298	2.56E-13	5.70E-12	1184
C18orf54	1.15E-10	1.295	2.69E-13	5.97E-12	1189
TREM2	2.48E-10	2.731	2.74E-13	6.05E-12	1191

MEIS2	7.54E-08	-1.579	2.74E-13	6.05E-12	1192
TPX2	1.68E-09	2.314	2.77E-13	6.11E-12	1196
FAM19A5	5.62E-07	-1.037	2.80E-13	6.16E-12	1199
WDHD1	4.78E-10	1.515	2.96E-13	6.48E-12	1202
ATOH8	2.33E-07	-2.506	3.00E-13	6.57E-12	1203
GALNS	4.13E-10	1.079	3.05E-13	6.67E-12	1204
TNC	1.21E-10	2.424	3.16E-13	6.90E-12	1208
COL16A1	1.94E-10	1.610	3.17E-13	6.91E-12	1210
HMGB3	1.12E-09	1.687	3.34E-13	7.23E-12	1218
TNFRSF4	1.23E-07	1.836	3.38E-13	7.31E-12	1219
TTYH3	7.59E-13	1.555	3.43E-13	7.40E-12	1221
CCL28	2.20E-03	-4.161	3.50E-13	7.54E-12	1224
TTPAL	1.39E-08	1.228	3.71E-13	7.97E-12	1229
CPEB3	2.72E-10	-1.615	4.19E-13	8.94E-12	1236
GMNN	1.00E-09	1.328	4.35E-13	9.24E-12	1240
LYN	1.16E-07	1.167	4.35E-13	9.25E-12	1241
SGIP1	6.59E-10	1.478	4.89E-13	1.03E-11	1250
PPM1L	3.84E-12	-2.125	5.00E-13	1.05E-11	1253
PYCARD	1.38E-07	1.143	5.03E-13	1.06E-11	1254
CD38	1.14E-04	1.460	5.20E-13	1.09E-11	1258
RAI2	1.17E-10	-1.842	5.50E-13	1.15E-11	1260
SDPR	5.44E-09	-1.945	5.76E-13	1.20E-11	1263
TRIP13	6.32E-10	2.178	5.80E-13	1.21E-11	1264
DNMT1	4.09E-10	1.180	5.81E-13	1.21E-11	1265
FOXO4	2.04E-10	-1.165	6.27E-13	1.30E-11	1269
RTKN	6.55E-10	1.129	6.47E-13	1.34E-11	1273
AADAT	4.31E-06	-1.012	6.55E-13	1.36E-11	1274
ARHGAP6	3.71E-10	-1.970	6.73E-13	1.39E-11	1275
ABI3BP	7.04E-12	-2.637	6.82E-13	1.41E-11	1276
GJC1	2.56E-11	2.265	7.12E-13	1.46E-11	1282
METTL24	1.02E-11	-2.045	7.22E-13	1.48E-11	1285
CA13	1.51E-09	-1.813	7.29E-13	1.49E-11	1286
PER2	5.69E-11	-1.123	7.47E-13	1.53E-11	1288
MCM10	4.00E-09	1.951	7.51E-13	1.54E-11	1289
KIF23	5.73E-09	2.190	7.91E-13	1.61E-11	1296
PDZRN3	1.07E-07	-1.566	8.15E-13	1.66E-11	1298
MOB3B	8.08E-10	1.373	8.17E-13	1.66E-11	1299
TMEM229B	1.20E-06	1.722	8.26E-13	1.67E-11	1301
CHEK1	8.38E-10	1.725	8.36E-13	1.69E-11	1302
CSRNP3	4.00E-09	-2.201	8.41E-13	1.70E-11	1303
ACOT7	2.85E-09	1.557	8.46E-13	1.71E-11	1304
SLC4A3	1.03E-08	1.419	8.67E-13	1.75E-11	1309

APLF	1.51E-12	-1.181	9.49E-13	1.90E-11	1315
L3MBTL4	2.56E-11	-2.066	9.65E-13	1.93E-11	1319
DBF4B	5.19E-09	1.218	1.04E-12	2.06E-11	1331
SPAG5	2.72E-09	1.737	1.05E-12	2.08E-11	1332
ATAD2	9.01E-08	1.434	1.08E-12	2.13E-11	1336
CTSF	9.55E-09	-2.029	1.10E-12	2.17E-11	1337
FAM189A2	1.27E-10	-2.739	1.19E-12	2.32E-11	1350
NFIC	1.46E-10	-1.262	1.22E-12	2.37E-11	1355
COL27A1	1.59E-10	1.644	1.22E-12	2.37E-11	1356
TOP1MT	3.26E-08	1.273	1.22E-12	2.38E-11	1357
PEG3	3.82E-11	-3.286	1.24E-12	2.40E-11	1358
APOD	9.86E-12	-3.259	1.29E-12	2.50E-11	1361
KRT14	4.18E-04	1.278	1.41E-12	2.71E-11	1368
LAG3	2.52E-09	2.558	1.43E-12	2.74E-11	1371
CENPI	3.01E-10	1.975	1.46E-12	2.81E-11	1374
LIPH	1.83E-03	-1.711	1.47E-12	2.82E-11	1377
MGLL	1.52E-10	-1.652	1.60E-12	3.04E-11	1381
COL1A1	5.25E-11	2.176	1.62E-12	3.09E-11	1383
DCLRE1B	8.67E-11	1.121	1.71E-12	3.24E-11	1390
ACSM3	2.66E-11	-2.590	1.74E-12	3.30E-11	1392
GCNT3	4.95E-04	-3.580	1.75E-12	3.32E-11	1393
NID2	4.29E-09	1.516	1.82E-12	3.43E-11	1398
GPR176	3.08E-11	2.096	1.95E-12	3.66E-11	1405
COTL1	5.27E-10	1.321	1.99E-12	3.73E-11	1408
SLC16A14	1.14E-11	-2.045	2.11E-12	3.93E-11	1415
GLI3	6.32E-09	1.329	2.24E-12	4.15E-11	1423
SULF1	1.47E-07	2.379	2.25E-12	4.17E-11	1424
IL17D	5.82E-03	-1.592	2.36E-12	4.36E-11	1428
MARCKSL1	9.22E-10	1.347	2.39E-12	4.39E-11	1432
IGFBP5	1.49E-10	-2.084	2.43E-12	4.47E-11	1436
MSRB1	4.63E-08	1.082	2.79E-12	5.09E-11	1445
TMEM232	1.76E-10	-2.075	2.84E-12	5.17E-11	1446
ABCC2	1.90E-04	1.731	2.85E-12	5.19E-11	1448
ABCA4	1.42E-09	2.810	2.97E-12	5.38E-11	1453
SLAMF7	3.71E-06	1.386	3.00E-12	5.44E-11	1454
PMFBP1	3.00E-06	1.411	3.01E-12	5.45E-11	1456
PKDCC	3.17E-09	-3.236	3.07E-12	5.55E-11	1457
HADH	4.71E-09	-1.593	3.16E-12	5.68E-11	1464
HJURP	4.15E-09	2.110	3.16E-12	5.68E-11	1465
RTP4	3.29E-07	1.778	3.25E-12	5.82E-11	1471
SLC7A7	3.00E-06	1.283	3.26E-12	5.84E-11	1472
PPL	1.93E-03	-1.288	3.26E-12	5.84E-11	1473

TANC2	4.64E-09	1.323	3.31E-12	5.92E-11	1475
RGS20	5.04E-07	2.179	3.34E-12	5.95E-11	1477
BTN3A3	1.83E-06	1.114	3.41E-12	6.07E-11	1480
LILRB1	8.36E-07	1.775	3.50E-12	6.22E-11	1486
ARSJ	4.70E-08	1.671	3.54E-12	6.27E-11	1488
CHN1	1.71E-11	1.998	3.59E-12	6.35E-11	1492
FIBIN	2.25E-05	-1.352	3.67E-12	6.48E-11	1495
ICA1	5.64E-08	-2.585	3.97E-12	6.96E-11	1503
KPNA2	8.39E-11	1.523	4.01E-12	7.01E-11	1506
SOD3	4.50E-11	-1.829	4.02E-12	7.03E-11	1508
ACTN1	2.33E-11	1.309	4.08E-12	7.13E-11	1510
CYBRD1	1.30E-08	-1.273	4.14E-12	7.20E-11	1515
ADAMTS7	5.66E-09	1.619	4.17E-12	7.25E-11	1517
CXCL10	3.92E-10	3.458	4.27E-12	7.41E-11	1520
TLR2	2.41E-07	1.355	4.40E-12	7.61E-11	1524
MYH11	8.68E-10	-3.584	4.57E-12	7.89E-11	1527
PCED1B	8.21E-09	1.349	4.64E-12	7.99E-11	1529
ZNF420	4.12E-12	-1.666	4.88E-12	8.39E-11	1535
IRF1	2.23E-06	1.029	4.91E-12	8.42E-11	1536
BRCA1	8.62E-11	1.339	4.95E-12	8.50E-11	1537
NLGN4X	4.59E-04	2.649	5.04E-12	8.63E-11	1539
TIGIT	2.04E-06	1.551	5.10E-12	8.73E-11	1541
CDC25A	2.26E-08	1.331	5.26E-12	8.99E-11	1544
IL12RB1	6.66E-07	1.856	5.66E-12	9.63E-11	1551
MYLIP	8.39E-11	-1.237	5.68E-12	9.64E-11	1553
POSTN	1.95E-10	3.209	5.81E-12	9.84E-11	1557
OGFRL1	1.68E-09	-1.185	6.25E-12	1.05E-10	1564
BRCA2	4.52E-08	1.525	6.31E-12	1.06E-10	1567
CKAP2L	4.99E-08	2.060	6.45E-12	1.08E-10	1569
IFI27	1.13E-09	1.687	6.55E-12	1.10E-10	1575
FXYD5	8.56E-12	1.709	6.65E-12	1.11E-10	1576
TRPC1	1.94E-09	-1.316	6.85E-12	1.14E-10	1578
ABCA8	2.31E-11	-2.972	6.99E-12	1.16E-10	1582
SETMAR	1.08E-11	-1.163	7.01E-12	1.17E-10	1584
KIAA1644	4.62E-08	1.886	7.27E-12	1.21E-10	1587
CHST2	1.75E-07	2.042	7.67E-12	1.27E-10	1594
NDRG1	8.40E-05	1.122	7.70E-12	1.27E-10	1595
SLC16A6	2.62E-05	-1.948	7.81E-12	1.29E-10	1596
NCAPH	2.32E-09	1.918	7.96E-12	1.31E-10	1599
ADAMTS15	7.38E-06	1.330	8.01E-12	1.32E-10	1600
KIF4A	3.15E-09	2.062	8.02E-12	1.32E-10	1601
AOC3	4.52E-10	-2.453	8.05E-12	1.33E-10	1602

GZMB	8.00E-09	2.387	8.16E-12	1.34E-10	1603
COL8A1	1.87E-09	2.141	8.22E-12	1.35E-10	1604
FOXM1	1.07E-08	2.083	8.76E-12	1.43E-10	1611
ORC6	1.95E-11	1.866	8.78E-12	1.44E-10	1612
UBE2C	4.48E-09	2.188	8.89E-12	1.45E-10	1614
TSPAN10	5.91E-07	2.438	9.09E-12	1.48E-10	1615
COL13A1	2.65E-08	1.641	9.56E-12	1.55E-10	1622
THBS2	4.84E-11	1.989	9.70E-12	1.57E-10	1626
PLK1	1.00E-09	2.410	9.75E-12	1.58E-10	1629
RCAN2	5.10E-07	-2.370	9.86E-12	1.59E-10	1632
MARC1	3.23E-10	-2.510	1.02E-11	1.63E-10	1638
ZBP1	8.50E-06	1.903	1.06E-11	1.70E-10	1645
GFI1	1.23E-08	1.784	1.17E-11	1.85E-10	1664
ZNF483	1.93E-08	-1.797	1.20E-11	1.90E-10	1666
COL5A1	4.55E-11	2.558	1.24E-11	1.96E-10	1670
FBLN1	1.68E-09	-1.252	1.26E-11	1.98E-10	1674
ACVR2A	1.02E-11	-1.234	1.37E-11	2.15E-10	1687
SLAMF8	8.21E-09	1.898	1.40E-11	2.19E-10	1690
ETV2	9.98E-04	-1.003	1.43E-11	2.23E-10	1692
SLC9A4	2.46E-08	-1.457	1.44E-11	2.24E-10	1693
KLF8	1.36E-04	-1.083	1.46E-11	2.28E-10	1694
SDK2	7.26E-07	1.715	1.51E-11	2.34E-10	1701
PPAP2B	3.56E-11	-1.826	1.58E-11	2.44E-10	1706
RAB31	1.63E-11	1.520	1.62E-11	2.50E-10	1709
C1QB	1.02E-05	1.195	1.64E-11	2.53E-10	1711
SLC24A3	1.01E-05	-1.269	1.66E-11	2.56E-10	1713
FAM63A	7.21E-10	-1.190	1.73E-11	2.65E-10	1719
VDR	1.20E-06	1.189	1.78E-11	2.72E-10	1727
CACNA2D2	4.24E-06	-1.883	1.79E-11	2.72E-10	1729
FAM229B	5.58E-07	-1.597	1.82E-11	2.77E-10	1732
TMEM132B	6.82E-09	-1.476	1.85E-11	2.81E-10	1734
TP53INP2	8.66E-06	-1.148	1.97E-11	2.98E-10	1743
PYGO1	3.81E-07	-1.900	1.98E-11	3.00E-10	1744
PTGS2	6.67E-04	1.281	1.99E-11	3.00E-10	1745
DCBLD2	8.82E-06	1.395	2.00E-11	3.02E-10	1746
SCUBE2	2.57E-04	-1.107	2.09E-11	3.14E-10	1752
FZD6	2.59E-08	1.402	2.15E-11	3.23E-10	1759
RAVER2	5.53E-09	-1.351	2.19E-11	3.27E-10	1765
PIF1	2.56E-10	1.986	2.20E-11	3.28E-10	1766
NBEA	1.23E-08	-2.947	2.21E-11	3.30E-10	1767
COL17A1	2.84E-04	1.467	2.27E-11	3.38E-10	1771
FADD	4.62E-07	1.449	2.37E-11	3.51E-10	1780

ADAD2	1.00E-03	-1.475	2.43E-11	3.60E-10	1783
CAV1	2.48E-06	1.259	2.45E-11	3.62E-10	1784
THY1	2.24E-10	1.507	2.46E-11	3.63E-10	1787
TGFB1	3.25E-09	1.525	2.55E-11	3.75E-10	1793
PSRC1	5.25E-08	1.585	2.60E-11	3.81E-10	1796
ANGPTL1	1.96E-12	-3.206	2.68E-11	3.92E-10	1805
CPXM1	1.43E-10	2.431	2.91E-11	4.24E-10	1811
ELF3	2.53E-02	-1.258	2.93E-11	4.26E-10	1812
IL21R	4.91E-09	2.060	2.96E-11	4.30E-10	1815
EDNRB	1.45E-08	-1.333	3.17E-11	4.58E-10	1827
DKK2	2.27E-07	-1.485	3.33E-11	4.79E-10	1834
KAZALD1	2.38E-06	-1.915	3.48E-11	4.97E-10	1844
RASGEF1A	2.10E-04	2.390	3.48E-11	4.98E-10	1845
SLC14A1	3.32E-08	-2.228	3.66E-11	5.20E-10	1854
ACPP	7.36E-06	-1.834	3.66E-11	5.21E-10	1855
B3GNT4	6.48E-06	1.523	3.69E-11	5.24E-10	1856
CCNF	2.45E-10	1.679	3.69E-11	5.24E-10	1857
VWA2	1.66E-04	-1.034	3.96E-11	5.59E-10	1869
CD300A	5.87E-05	1.125	4.07E-11	5.73E-10	1874
CACNA1D	2.01E-07	-2.231	4.08E-11	5.73E-10	1875
SYBU	1.59E-08	-1.992	4.12E-11	5.78E-10	1879
UBL3	1.86E-12	-1.239	4.20E-11	5.88E-10	1882
NYNRIN	6.73E-09	-1.485	4.25E-11	5.95E-10	1883
DLX1	7.30E-06	1.541	4.25E-11	5.95E-10	1884
MX1	2.15E-10	1.759	4.35E-11	6.07E-10	1889
SBSPON	1.28E-08	-1.741	4.48E-11	6.24E-10	1895
NPR1	5.77E-08	-1.660	4.60E-11	6.39E-10	1899
RNF150	7.76E-10	-2.618	4.75E-11	6.58E-10	1903
FHIT	4.30E-11	-2.141	4.78E-11	6.61E-10	1905
AOX1	1.00E-09	-2.724	4.83E-11	6.68E-10	1907
SOCS1	2.76E-09	1.671	4.94E-11	6.81E-10	1910
ZNF554	2.51E-10	-1.020	5.17E-11	7.10E-10	1921
PRLR	8.52E-10	-2.728	5.17E-11	7.10E-10	1922
SLIT3	2.67E-11	-2.433	5.21E-11	7.14E-10	1924
IL7R	1.22E-08	1.987	5.51E-11	7.53E-10	1931
MYH10	1.17E-07	1.081	5.70E-11	7.76E-10	1937
ZC3H6	5.61E-11	-1.174	5.74E-11	7.81E-10	1938
LIMS2	1.10E-09	-1.621	5.84E-11	7.93E-10	1943
ALDH3A1	2.34E-05	-2.434	6.11E-11	8.27E-10	1947
FCER1A	1.00E-09	-2.042	6.23E-11	8.43E-10	1949
PGM5	2.12E-09	-2.837	6.26E-11	8.46E-10	1950
TMEM150B	2.25E-05	1.484	6.30E-11	8.51E-10	1952

CORO2B	2.88E-08	-1.413	6.56E-11	8.84E-10	1956
SLAMF6	3.91E-04	1.307	6.64E-11	8.94E-10	1958
NCAPG	1.33E-09	1.976	6.73E-11	9.05E-10	1959
KIF18A	1.59E-09	2.088	7.08E-11	9.49E-10	1968
LAMA1	1.20E-08	2.575	7.32E-11	9.77E-10	1975
AMOT	1.88E-09	-3.393	7.51E-11	1.00E-09	1979
SEMA7A	1.53E-08	1.657	8.04E-11	1.07E-09	1988
HELZ2	7.21E-12	1.756	8.21E-11	1.09E-09	1991
TPPP3	7.63E-04	-1.154	8.24E-11	1.09E-09	1992
AMPD3	4.09E-07	1.124	8.27E-11	1.09E-09	1993
F2RL2	9.52E-08	1.858	8.75E-11	1.15E-09	1997
IRX5	1.73E-06	-1.229	8.92E-11	1.18E-09	2001
ABLIM2	1.59E-03	-1.301	8.96E-11	1.18E-09	2003
CDH11	4.97E-08	1.598	9.02E-11	1.19E-09	2004
PALM	2.36E-11	-2.152	9.07E-11	1.19E-09	2006
CA2	3.53E-04	1.627	9.07E-11	1.19E-09	2007
AK8	5.89E-08	-1.285	9.53E-11	1.25E-09	2013
TRIL	2.54E-09	-1.807	9.56E-11	1.25E-09	2014
MST1R	2.04E-05	1.393	9.96E-11	1.30E-09	2021
DAGLA	1.10E-07	-1.721	1.01E-10	1.32E-09	2025
MTFR2	3.70E-09	1.721	1.02E-10	1.32E-09	2026
IL15RA	3.22E-09	1.128	1.12E-10	1.44E-09	2035
COL10A1	3.26E-08	2.959	1.12E-10	1.45E-09	2036
DTL	4.09E-10	1.845	1.14E-10	1.48E-09	2038
MAL	1.48E-08	-3.729	1.15E-10	1.48E-09	2039
PTGR1	2.72E-04	-1.074	1.16E-10	1.50E-09	2040
GSG2	1.93E-10	1.750	1.17E-10	1.51E-09	2042
ICA1L	5.25E-11	-1.671	1.18E-10	1.52E-09	2043
BUB1	7.20E-09	2.001	1.20E-10	1.55E-09	2045
CYP4F3	2.25E-05	-1.565	1.23E-10	1.58E-09	2051
PIK3CD	2.95E-09	1.272	1.28E-10	1.64E-09	2057
PODN	1.72E-09	-2.329	1.31E-10	1.67E-09	2065
KIAA1147	2.54E-06	-1.234	1.39E-10	1.77E-09	2079
SP140	2.05E-05	1.482	1.45E-10	1.83E-09	2085
CARD11	8.80E-07	1.918	1.45E-10	1.83E-09	2087
ITM2A	5.14E-10	-1.705	1.46E-10	1.85E-09	2088
MSC	7.24E-05	1.259	1.48E-10	1.87E-09	2090
EPS8	1.92E-08	-1.032	1.53E-10	1.92E-09	2097
LRRK2	8.08E-10	-1.500	1.55E-10	1.94E-09	2099
RAD51AP1	1.10E-09	1.787	1.59E-10	2.00E-09	2104
GPRC5B	1.15E-07	-2.197	1.68E-10	2.09E-09	2116
MX2	4.58E-09	1.874	1.71E-10	2.12E-09	2120

MYBL2	2.58E-08	2.409	1.79E-10	2.22E-09	2126
KIAA1549L	2.11E-03	1.410	1.94E-10	2.40E-09	2132
LAMB3	1.99E-05	1.776	1.95E-10	2.41E-09	2134
ADCY6	2.08E-10	-1.392	2.05E-10	2.52E-09	2140
FPR3	1.15E-07	1.634	2.07E-10	2.55E-09	2144
ACSS3	7.22E-09	-2.777	2.07E-10	2.55E-09	2145
SLC13A4	1.16E-07	-1.482	2.11E-10	2.59E-09	2148
TRANK1	3.32E-08	1.103	2.15E-10	2.64E-09	2151
CD86	3.03E-07	1.416	2.27E-10	2.77E-09	2161
SKA3	1.16E-08	1.709	2.38E-10	2.90E-09	2167
SERTAD4	9.63E-08	-1.330	2.41E-10	2.93E-09	2169
ASCL2	9.55E-09	1.738	2.41E-10	2.93E-09	2170
SGOL2	5.52E-09	1.473	2.44E-10	2.96E-09	2173
PPP1R14C	2.92E-06	1.637	2.59E-10	3.13E-09	2181
EGFL6	7.75E-09	2.187	2.60E-10	3.14E-09	2182
RBP1	2.83E-07	2.617	2.62E-10	3.16E-09	2184
FYB	1.75E-05	1.250	2.71E-10	3.26E-09	2188
RECQL4	1.47E-10	1.595	2.75E-10	3.31E-09	2189
CDCA3	9.12E-09	1.964	2.78E-10	3.35E-09	2191
NDC80	1.08E-09	2.020	2.83E-10	3.40E-09	2195
CASC5	8.91E-08	1.824	2.86E-10	3.43E-09	2199
MGP	7.45E-10	-2.393	2.88E-10	3.45E-09	2201
SIRPB1	3.15E-06	1.303	3.00E-10	3.57E-09	2212
BHMT2	1.23E-08	-2.306	3.03E-10	3.60E-09	2215
MYO7A	1.97E-06	1.152	3.04E-10	3.61E-09	2218
ZNF570	3.86E-11	-1.410	3.16E-10	3.74E-09	2230
BICD1	8.00E-09	1.058	3.18E-10	3.75E-09	2232
DLGAP4	5.09E-08	1.114	3.31E-10	3.90E-09	2236
ZNF423	8.87E-07	-1.542	3.38E-10	3.98E-09	2240
LRP3	6.98E-10	-1.664	3.49E-10	4.10E-09	2244
LUM	7.48E-08	1.218	3.54E-10	4.15E-09	2247
BOP1	2.72E-07	1.203	3.56E-10	4.17E-09	2248
CBX7	7.79E-10	-1.850	3.65E-10	4.27E-09	2255
LMOD1	1.51E-09	-2.790	3.66E-10	4.28E-09	2256
ZNF135	1.53E-09	-1.909	3.71E-10	4.32E-09	2259
TBKBP1	3.32E-08	-1.468	3.73E-10	4.35E-09	2261
PIP5KL1	6.49E-05	1.444	3.80E-10	4.42E-09	2267
EOMES	2.28E-05	1.416	3.82E-10	4.44E-09	2268
JAK3	4.68E-05	1.115	3.84E-10	4.46E-09	2270
ABCA3	4.28E-10	-2.223	3.86E-10	4.48E-09	2271
LIPG	8.04E-07	1.522	3.91E-10	4.54E-09	2273
SMIM5	3.64E-07	-1.636	3.94E-10	4.57E-09	2276

FAM111B	2.64E-07	1.720	4.15E-10	4.79E-09	2284
FAM83A	1.62E-05	2.347	4.21E-10	4.84E-09	2289
MAP1LC3A	1.32E-11	-1.809	4.39E-10	5.05E-09	2294
SSC5D	3.58E-07	-1.463	4.41E-10	5.06E-09	2295
CENPL	1.09E-10	1.140	4.47E-10	5.13E-09	2298
PRRT3	1.00E-07	-1.133	4.52E-10	5.18E-09	2299
KBTBD3	9.65E-07	-1.265	4.61E-10	5.28E-09	2302
FREM1	3.67E-11	-2.065	4.77E-10	5.45E-09	2306
MITF	2.31E-08	-2.088	4.77E-10	5.46E-09	2307
DLGAP5	2.28E-08	2.163	5.15E-10	5.84E-09	2325
CCNB2	3.12E-09	1.958	5.32E-10	6.00E-09	2334
HSF2BP	5.19E-09	1.737	5.44E-10	6.13E-09	2336
CAPN14	1.72E-05	-2.434	5.67E-10	6.37E-09	2345
FANCI	4.21E-09	1.577	5.94E-10	6.64E-09	2358
FMNL3	1.69E-11	1.321	5.96E-10	6.66E-09	2361
IL27RA	9.67E-07	1.075	6.02E-10	6.71E-09	2365
PRC1	5.46E-09	1.551	6.31E-10	7.00E-09	2376
ANK2	5.29E-10	-2.887	6.58E-10	7.28E-09	2383
FGF2	5.48E-08	-1.332	6.59E-10	7.29E-09	2384
COL3A1	1.30E-10	1.705	6.78E-10	7.48E-09	2389
RPS6KA4	2.27E-09	1.384	7.66E-10	8.37E-09	2413
ZNF571	1.32E-10	-1.421	7.79E-10	8.50E-09	2418
SLC25A23	4.72E-12	-1.590	7.90E-10	8.60E-09	2423
SIGLEC10	2.83E-07	1.421	8.02E-10	8.72E-09	2425
HAVCR2	2.03E-06	1.514	8.05E-10	8.74E-09	2426
RUNX1T1	1.38E-09	-1.493	8.06E-10	8.76E-09	2427
PKMYT1	2.68E-11	2.127	8.14E-10	8.83E-09	2429
LPIN1	2.28E-04	-1.074	8.49E-10	9.16E-09	2443
ISG20	9.04E-06	1.333	8.66E-10	9.35E-09	2444
PER3	2.51E-08	-1.273	8.68E-10	9.36E-09	2445
BMP8A	1.11E-09	1.981	8.92E-10	9.60E-09	2451
KIF20A	3.00E-09	2.122	8.93E-10	9.60E-09	2452
HOXD10	4.05E-08	2.396	9.35E-10	1.00E-08	2465
GNAZ	6.85E-09	-1.572	9.47E-10	1.01E-08	2466
LYVE1	8.83E-09	-2.049	9.67E-10	1.03E-08	2471
CCL5	9.82E-08	1.657	9.87E-10	1.05E-08	2475
ETV7	4.66E-07	1.814	9.88E-10	1.05E-08	2476
POU2F2	4.62E-06	1.335	1.01E-09	1.07E-08	2484
PPARG	1.22E-10	-2.459	1.09E-09	1.15E-08	2495
LGI4	6.00E-09	-1.732	1.09E-09	1.15E-08	2499
APBB1	6.85E-07	-1.440	1.14E-09	1.20E-08	2509
DBNDD1	2.92E-09	-2.005	1.14E-09	1.20E-08	2510

SNAPC1	3.99E-08	1.045	1.24E-09	1.30E-08	2528
C20orf197	4.30E-03	2.621	1.27E-09	1.32E-08	2531
TACC3	1.00E-09	1.312	1.30E-09	1.35E-08	2536
RDH16	2.76E-07	1.206	1.32E-09	1.38E-08	2538
GGT6	2.02E-02	-1.357	1.33E-09	1.38E-08	2539
ZNF717	1.38E-10	-1.913	1.33E-09	1.38E-08	2541
DSE	2.39E-08	1.203	1.41E-09	1.46E-08	2548
SHCBP1	3.70E-10	1.999	1.42E-09	1.47E-08	2551
PITX1	1.80E-05	-1.382	1.44E-09	1.48E-08	2553
PPP1R3C	3.00E-09	-3.739	1.44E-09	1.49E-08	2558
HKR1	2.44E-07	-1.122	1.46E-09	1.50E-08	2559
ZNF469	1.69E-10	2.172	1.46E-09	1.50E-08	2560
STRA6	1.54E-05	2.010	1.48E-09	1.52E-08	2564
RAD51	1.36E-10	1.875	1.53E-09	1.57E-08	2572
NTN4	1.00E-08	-1.275	1.54E-09	1.57E-08	2573
KCNAB1	4.11E-09	-1.257	1.60E-09	1.63E-08	2586
SEMA3G	1.21E-07	-1.581	1.60E-09	1.63E-08	2587
GRIK5	4.57E-06	-1.076	1.67E-09	1.69E-08	2596
RUNDC3A	1.01E-04	2.190	1.71E-09	1.73E-08	2603
DNA2	8.70E-09	1.116	1.74E-09	1.76E-08	2608
CDKN3	1.20E-11	1.951	1.82E-09	1.84E-08	2617
ELN	1.98E-07	-1.823	1.89E-09	1.90E-08	2624
REEP2	4.74E-09	-2.100	1.96E-09	1.96E-08	2630
NPTXR	3.93E-09	-2.225	1.96E-09	1.96E-08	2634
KREMEN2	6.06E-06	2.191	1.98E-09	1.97E-08	2637
COBL	1.71E-09	-2.647	2.05E-09	2.05E-08	2649
SYT12	7.48E-08	2.574	2.07E-09	2.06E-08	2651
POLQ	1.01E-07	1.485	2.08E-09	2.07E-08	2652
NPDC1	1.57E-08	-2.170	2.15E-09	2.13E-08	2662
CPNE5	5.23E-03	1.010	2.58E-09	2.52E-08	2698
PAQR8	5.20E-04	-1.156	2.60E-09	2.54E-08	2699
MAB21L2	3.16E-08	-1.972	2.60E-09	2.54E-08	2701
ZNF514	1.75E-09	-1.041	2.65E-09	2.58E-08	2708
TMPRSS11A	1.05E-05	-2.689	2.72E-09	2.64E-08	2716
MMP17	7.48E-08	1.738	2.86E-09	2.76E-08	2727
TCEA3	3.78E-09	-3.163	2.86E-09	2.77E-08	2729
BEND7	4.94E-07	-2.544	2.90E-09	2.79E-08	2733
ZFP14	2.38E-07	-1.157	2.93E-09	2.82E-08	2737
ANLN	2.94E-07	2.057	3.10E-09	2.97E-08	2750
RIBC1	5.67E-08	-1.079	3.15E-09	3.01E-08	2755
HIST1H2BO	1.32E-08	1.902	3.18E-09	3.04E-08	2760
PLS1	8.33E-03	-1.155	3.26E-09	3.11E-08	2768

NFIB	1.11E-07	-1.520	3.30E-09	3.14E-08	2770
LAX1	6.85E-03	1.232	3.30E-09	3.14E-08	2771
NR4A2	8.74E-07	-1.888	3.42E-09	3.25E-08	2780
ZNF287	3.48E-11	-1.788	3.81E-09	3.58E-08	2804
OAS1	8.32E-08	1.388	3.86E-09	3.62E-08	2811
JAKMIP1	1.38E-04	1.307	3.93E-09	3.68E-08	2817
PAIP2B	5.47E-11	-2.401	4.05E-09	3.78E-08	2822
TACR1	4.63E-10	-2.022	4.21E-09	3.92E-08	2833
GPR171	6.41E-05	1.392	4.40E-09	4.08E-08	2842
ERCC6L	4.15E-09	1.517	4.74E-09	4.38E-08	2854
COL7A1	2.51E-07	1.692	4.76E-09	4.40E-08	2856
SMAD9	5.90E-07	-2.717	4.98E-09	4.58E-08	2869
SERINC2	1.71E-06	1.231	5.16E-09	4.73E-08	2878
LCK	5.58E-05	1.175	5.17E-09	4.74E-08	2879
C16orf59	2.32E-09	1.865	5.25E-09	4.80E-08	2884
PIGZ	2.14E-09	-1.289	5.38E-09	4.91E-08	2888
BIRC5	1.63E-08	2.035	5.38E-09	4.91E-08	2889
CRY2	1.00E-09	-1.347	5.53E-09	5.03E-08	2895
VNN1	1.46E-02	1.591	5.57E-09	5.07E-08	2898
EPHX1	9.74E-11	-1.236	5.59E-09	5.08E-08	2901
SHC3	5.23E-06	-1.199	5.64E-09	5.12E-08	2905
SPAG16	8.47E-09	-1.687	5.77E-09	5.23E-08	2910
SLC10A1	3.45E-11	-1.483	6.14E-09	5.54E-08	2921
PRADC1	2.06E-09	-1.276	6.25E-09	5.64E-08	2924
IL2RG	2.38E-04	1.035	6.55E-09	5.87E-08	2940
FGF12	1.53E-04	-1.773	7.05E-09	6.28E-08	2959
KLHL6	2.84E-05	1.175	7.23E-09	6.43E-08	2965
TGFA	3.99E-06	1.464	7.35E-09	6.53E-08	2967
PPM1H	1.49E-03	-2.734	7.43E-09	6.60E-08	2968
ZNF682	3.22E-09	-1.465	7.53E-09	6.68E-08	2971
PLOD2	1.18E-04	1.578	8.05E-09	7.10E-08	2987
TSPAN12	2.38E-06	-2.984	8.05E-09	7.11E-08	2988
MAGI2	8.48E-09	-1.843	8.28E-09	7.29E-08	2994
MOCOS	2.60E-07	1.265	8.44E-09	7.42E-08	2999
BMP2	2.00E-05	1.256	8.62E-09	7.55E-08	3007
GM2A	4.81E-06	1.054	8.69E-09	7.61E-08	3009
PTPRZ1	5.69E-05	1.829	8.86E-09	7.75E-08	3013
KRBA2	1.08E-07	-1.520	9.18E-09	8.00E-08	3024
ZNF345	3.45E-10	-1.345	9.61E-09	8.36E-08	3031
ZWINT	2.04E-07	1.413	9.84E-09	8.55E-08	3034
LMNB2	1.42E-08	1.174	1.09E-08	9.37E-08	3062
EEPD1	2.89E-07	-1.132	1.10E-08	9.46E-08	3066

DIAPH3	1.08E-06	1.558	1.17E-08	1.00E-07	3083
CERCAM	1.10E-10	1.336	1.17E-08	1.00E-07	3085
KLHDC1	1.00E-09	-1.714	1.20E-08	1.02E-07	3092
POLR3G	3.43E-05	1.119	1.20E-08	1.02E-07	3093
LTC4S	2.73E-09	-1.235	1.21E-08	1.03E-07	3098
ADAM19	3.46E-07	1.678	1.24E-08	1.05E-07	3106
GFRA1	1.06E-10	-3.021	1.25E-08	1.06E-07	3107
FAM184A	6.85E-10	-2.605	1.26E-08	1.07E-07	3108
LAIR1	3.05E-05	1.221	1.40E-08	1.18E-07	3132
CD247	1.51E-06	1.411	1.45E-08	1.21E-07	3141
ZNF358	4.81E-06	-1.123	1.49E-08	1.25E-07	3154
MPP7	9.55E-06	-1.041	1.59E-08	1.32E-07	3171
HSD11B2	9.28E-04	-1.133	1.69E-08	1.40E-07	3190
PCYT1A	4.26E-07	1.033	1.74E-08	1.44E-07	3195
ADTRP	8.97E-08	2.172	1.79E-08	1.47E-07	3200
RNASEH2A	4.13E-10	1.251	1.84E-08	1.51E-07	3205
MYH14	8.08E-10	-2.574	1.91E-08	1.57E-07	3214
SUCNR1	2.19E-06	1.515	2.10E-08	1.70E-07	3240
SESN1	9.50E-07	-1.231	2.11E-08	1.72E-07	3243
CDH24	4.65E-08	1.335	2.25E-08	1.82E-07	3263
SQLE	3.06E-05	1.440	2.37E-08	1.91E-07	3276
CNTNAP3	2.06E-04	-1.119	2.38E-08	1.92E-07	3277
RACGAP1	8.54E-08	1.206	2.46E-08	1.97E-07	3287
TMEM47	7.74E-07	-1.584	2.48E-08	1.99E-07	3288
ZNF354C	1.59E-10	-1.531	2.52E-08	2.02E-07	3294
EPHB2	5.17E-09	2.068	2.63E-08	2.10E-07	3306
SLC7A2	1.10E-09	-2.633	2.64E-08	2.11E-07	3309
CARNS1	3.24E-04	-3.231	2.69E-08	2.14E-07	3313
SH3D21	1.79E-04	1.271	2.77E-08	2.20E-07	3318
RANBP17	1.05E-03	-2.461	2.81E-08	2.23E-07	3320
EDIL3	6.38E-08	1.483	2.82E-08	2.24E-07	3321
APOBEC3A	1.33E-04	1.048	2.86E-08	2.27E-07	3323
HAGHL	7.26E-08	1.182	2.90E-08	2.30E-07	3325
RIN1	1.56E-06	1.612	2.90E-08	2.30E-07	3326
RASL12	1.53E-09	-1.803	2.92E-08	2.31E-07	3328
ZNF774	1.18E-06	-1.292	2.98E-08	2.36E-07	3330
PTPN7	1.56E-05	1.233	3.00E-08	2.37E-07	3332
AJAP1	1.76E-04	2.563	3.04E-08	2.40E-07	3338
LY6K	1.47E-07	2.723	3.09E-08	2.44E-07	3346
BFSP1	2.79E-05	1.217	3.16E-08	2.48E-07	3355
MKI67	3.99E-07	1.740	3.24E-08	2.54E-07	3360
CD2	1.13E-04	1.249	3.27E-08	2.56E-07	3365

CAMK4	1.58E-05	1.418	3.33E-08	2.60E-07	3369
SSBP2	5.13E-10	-1.261	3.47E-08	2.70E-07	3383
LOX	2.54E-06	1.661	3.60E-08	2.80E-07	3390
CLEC7A	4.67E-07	1.170	3.64E-08	2.83E-07	3398
ASAP3	1.24E-10	-1.566	3.70E-08	2.86E-07	3403
ZBTB7C	8.15E-11	-2.238	3.80E-08	2.93E-07	3412
ZNF433	6.89E-11	-1.573	3.83E-08	2.96E-07	3414
MACROD1	1.17E-06	-2.223	3.94E-08	3.03E-07	3425
PPIF	1.02E-04	1.011	4.04E-08	3.10E-07	3435
KIF20B	3.29E-08	1.219	4.07E-08	3.12E-07	3437
MCM4	3.67E-08	1.296	4.16E-08	3.18E-07	3444
FCGBP	4.12E-04	-2.937	4.16E-08	3.18E-07	3445
CETP	8.06E-11	1.430	4.20E-08	3.21E-07	3448
F8	2.45E-05	-1.269	4.25E-08	3.24E-07	3453
ZNF569	2.00E-09	-1.367	4.36E-08	3.32E-07	3462
B4GALNT1	3.75E-05	1.344	4.54E-08	3.45E-07	3469
RAI14	4.67E-06	1.267	4.60E-08	3.50E-07	3471
ZNF667	4.14E-07	-2.044	4.64E-08	3.52E-07	3475
CELSR3	4.58E-09	2.153	4.80E-08	3.64E-07	3481
IQGAP3	2.44E-07	1.488	4.88E-08	3.69E-07	3485
NDRG2	2.02E-07	-2.476	4.92E-08	3.72E-07	3488
GDPD2	5.11E-06	2.817	5.00E-08	3.78E-07	3492
DLEU7	2.31E-09	1.550	5.09E-08	3.83E-07	3496
CDCA8	1.44E-08	1.792	5.10E-08	3.85E-07	3497
HERC6	1.21E-06	1.493	5.29E-08	3.98E-07	3505
CENPK	2.16E-07	1.609	5.48E-08	4.11E-07	3517
ZCWPW2	2.24E-09	-2.280	5.61E-08	4.20E-07	3526
CEACAM1	1.40E-06	-1.991	5.66E-08	4.23E-07	3530
F3	1.27E-02	1.040	5.85E-08	4.36E-07	3540
EBI3	2.38E-05	1.405	6.61E-08	4.86E-07	3584
CCM2L	5.79E-06	-1.079	6.76E-08	4.97E-07	3589
IL18BP	1.36E-07	1.451	6.87E-08	5.04E-07	3598
SLA2	1.00E-05	1.432	7.41E-08	5.40E-07	3617
EPHB6	4.89E-06	-1.286	7.43E-08	5.41E-07	3618
ZNF626	5.61E-11	-2.078	7.44E-08	5.42E-07	3619
MYB	1.65E-05	-1.186	7.54E-08	5.48E-07	3623
WSCD1	1.06E-07	-2.187	7.59E-08	5.51E-07	3627
CAPS	2.21E-06	-1.273	7.64E-08	5.54E-07	3632
ENDOU	1.66E-04	-2.279	8.00E-08	5.79E-07	3645
PATL2	4.27E-04	1.329	8.22E-08	5.93E-07	3655
RASL11B	1.64E-06	1.497	8.71E-08	6.25E-07	3675
OLFML2B	3.47E-09	1.469	8.89E-08	6.37E-07	3679

CDK1	1.00E-08	1.681	8.96E-08	6.41E-07	3685
LAPTM5	1.00E-05	1.053	9.11E-08	6.50E-07	3691
AMIGO1	4.30E-08	-1.875	9.14E-08	6.52E-07	3692
CLIC6	2.21E-07	-2.105	9.40E-08	6.70E-07	3699
PRSS27	2.62E-03	-1.493	9.67E-08	6.88E-07	3706
FERMT1	6.90E-06	1.598	9.77E-08	6.94E-07	3712
GYLTL1B	5.95E-04	1.508	1.02E-07	7.23E-07	3725
PLEKHG2	1.49E-10	1.190	1.02E-07	7.24E-07	3727
ZC2HC1C	2.49E-09	-1.316	1.03E-07	7.27E-07	3730
IL1A	8.65E-06	1.783	1.05E-07	7.44E-07	3737
SUSD4	5.22E-08	-1.921	1.06E-07	7.49E-07	3742
FAM134B	1.01E-04	-1.849	1.12E-07	7.86E-07	3760
CBR3	3.62E-05	-1.150	1.13E-07	7.89E-07	3762
CDC7	4.22E-09	1.302	1.13E-07	7.94E-07	3766
SLC1A1	9.92E-09	-1.449	1.14E-07	7.97E-07	3769
FAM110D	3.70E-06	-1.115	1.20E-07	8.36E-07	3786
MCM2	1.21E-09	1.349	1.24E-07	8.60E-07	3802
CYP2J2	2.90E-10	-2.155	1.25E-07	8.64E-07	3805
H2AFX	6.37E-08	1.207	1.25E-07	8.67E-07	3806
ACHE	4.95E-05	-2.947	1.26E-07	8.72E-07	3808
CDK18	2.76E-05	1.352	1.26E-07	8.73E-07	3809
TK1	1.36E-08	1.687	1.35E-07	9.29E-07	3824
NOSTRIN	3.07E-06	-2.279	1.38E-07	9.47E-07	3835
BNC2	3.09E-07	-2.427	1.38E-07	9.48E-07	3836
CCR7	1.97E-05	1.463	1.41E-07	9.67E-07	3841
ZNF615	6.10E-09	-1.160	1.45E-07	9.92E-07	3854
ZNF528	3.90E-10	-1.637	1.57E-07	1.07E-06	3884
ZNF573	1.92E-08	-1.192	1.62E-07	1.10E-06	3896
ZNF582	4.25E-08	-1.539	1.69E-07	1.14E-06	3912
NEGR1	1.69E-06	-1.440	1.71E-07	1.15E-06	3915
PYHIN1	5.65E-04	1.201	1.81E-07	1.22E-06	3933
PLK4	2.38E-07	1.353	1.83E-07	1.23E-06	3937
SPRY2	2.91E-10	-1.397	1.98E-07	1.32E-06	3966
NRIP2	7.35E-08	-1.423	1.98E-07	1.32E-06	3967
EDNRA	1.69E-07	1.305	2.04E-07	1.35E-06	3980
THSD4	1.01E-04	-1.010	2.11E-07	1.39E-06	3997
CD3D	8.87E-05	1.240	2.12E-07	1.40E-06	3998
ALOX12	5.59E-07	-2.820	2.18E-07	1.44E-06	4005
CDCA2	4.99E-08	1.771	2.23E-07	1.46E-06	4012
RUNX3	1.37E-07	1.217	2.23E-07	1.46E-06	4013
MYO3B	1.99E-05	2.073	2.25E-07	1.48E-06	4016
SVIP	7.14E-10	-2.088	2.58E-07	1.67E-06	4068

ZNF396	1.17E-05	-1.696	2.74E-07	1.77E-06	4090
IL18R1	2.16E-06	1.269	2.75E-07	1.77E-06	4092
RRM2	1.03E-06	1.738	2.86E-07	1.84E-06	4102
STK39	6.18E-04	-1.062	2.86E-07	1.84E-06	4103
ZNF273	1.18E-08	-1.167	2.88E-07	1.85E-06	4108
DEPDC1	5.84E-08	1.736	2.91E-07	1.87E-06	4113
SKA1	5.03E-08	2.008	3.04E-07	1.94E-06	4130
SYNPO2	8.82E-07	-2.280	3.22E-07	2.04E-06	4152
BBS7	4.71E-10	1.193	3.29E-07	2.08E-06	4159
ANKRD35	1.21E-07	-1.452	3.31E-07	2.10E-06	4164
MYEF2	9.72E-08	-1.629	3.39E-07	2.14E-06	4171
CXCR3	1.81E-04	1.374	3.46E-07	2.18E-06	4182
HIST1H2AK	1.85E-07	1.420	3.53E-07	2.22E-06	4191
FOSB	1.74E-06	-1.949	3.59E-07	2.25E-06	4199
HSPA12B	1.75E-05	-1.075	3.65E-07	2.29E-06	4207
PIEZO2	7.76E-07	1.258	3.65E-07	2.29E-06	4208
PIK3R1	1.41E-07	-1.030	4.01E-07	2.49E-06	4246
HIST1H2BF	1.13E-07	1.571	4.33E-07	2.68E-06	4264
ORC1	5.71E-08	1.785	4.46E-07	2.75E-06	4275
SPEF2	2.56E-09	-1.720	4.49E-07	2.77E-06	4277
JAM2	1.60E-07	-1.106	4.49E-07	2.77E-06	4278
ATP6V0E2	7.88E-08	-2.029	4.50E-07	2.77E-06	4280
WBSCR17	3.55E-04	-1.266	4.53E-07	2.79E-06	4286
GBP4	1.00E-09	2.921	4.56E-07	2.80E-06	4292
CCL18	2.26E-03	1.031	4.58E-07	2.81E-06	4296
KRT16	3.58E-04	1.348	4.70E-07	2.87E-06	4317
PRKG2	1.03E-06	-1.571	4.74E-07	2.89E-06	4321
TP63	1.55E-04	1.094	4.74E-07	2.89E-06	4322
KIAA1549	1.45E-03	-1.381	4.99E-07	3.03E-06	4342
GAMT	1.56E-07	-2.853	5.18E-07	3.14E-06	4353
RIMS3	1.07E-02	1.158	5.22E-07	3.16E-06	4358
C17orf53	2.29E-08	1.399	5.22E-07	3.16E-06	4360
ITIH5	1.30E-08	-1.553	5.39E-07	3.25E-06	4370
CES1	2.82E-09	-2.095	5.52E-07	3.32E-06	4381
CCR4	7.13E-04	1.211	5.58E-07	3.35E-06	4389
ADHFE1	4.70E-09	-3.111	5.63E-07	3.38E-06	4396
HELLS	1.08E-06	1.225	5.81E-07	3.47E-06	4409
RELN	1.42E-08	-2.169	6.09E-07	3.63E-06	4428
SOBP	1.13E-06	-2.091	6.35E-07	3.77E-06	4442
SPATA18	1.15E-08	-1.633	6.36E-07	3.78E-06	4443
RYR1	1.06E-02	-2.664	6.37E-07	3.78E-06	4444
FAM198A	8.00E-09	-1.185	6.71E-07	3.96E-06	4467

EDA	7.39E-09	-1.239	7.11E-07	4.17E-06	4494
TC2N	1.57E-06	-1.254	7.34E-07	4.29E-06	4512
LRP6	2.34E-08	-1.216	7.43E-07	4.34E-06	4519
BCAS4	4.78E-06	1.114	7.68E-07	4.46E-06	4533
CLMN	4.73E-05	-1.357	7.80E-07	4.53E-06	4543
HIST1H2BN	1.99E-06	1.277	8.08E-07	4.67E-06	4559
GHR	1.21E-07	-1.571	8.10E-07	4.68E-06	4561
GPRASP1	5.36E-11	-2.050	8.30E-07	4.78E-06	4572
ZNF491	4.10E-08	-1.887	8.54E-07	4.91E-06	4583
MCOLN2	5.18E-04	1.155	8.55E-07	4.92E-06	4584
FAM171A1	2.50E-08	-1.354	8.92E-07	5.11E-06	4605
GPRIN2	6.76E-04	-1.771	8.92E-07	5.11E-06	4606
RGS11	1.58E-11	-1.941	8.95E-07	5.12E-06	4607
NETO2	3.50E-07	1.409	9.08E-07	5.19E-06	4615
PPP1R12B	1.43E-07	-2.101	9.49E-07	5.40E-06	4635
RERG	1.12E-09	-1.964	9.58E-07	5.44E-06	4640
CD3G	9.31E-05	1.213	9.86E-07	5.59E-06	4650
SCARA3	2.48E-08	-1.304	1.10E-06	6.19E-06	4694
CYP4X1	1.38E-09	-2.883	1.11E-06	6.23E-06	4696
HIST1H2AM	4.23E-07	1.443	1.14E-06	6.40E-06	4706
TTK	4.12E-07	1.624	1.17E-06	6.54E-06	4716
TMX4	1.17E-06	-1.069	1.20E-06	6.71E-06	4728
ZNF208	2.72E-09	-1.474	1.21E-06	6.76E-06	4735
SLC7A11	6.76E-05	1.162	1.23E-06	6.84E-06	4742
KCNIP3	2.83E-07	-1.147	1.27E-06	7.04E-06	4757
MAP6	1.32E-07	-1.538	1.27E-06	7.04E-06	4759
PALMD	9.34E-09	-1.547	1.29E-06	7.13E-06	4767
CLDN5	5.36E-06	-1.679	1.30E-06	7.18E-06	4770
ATP1B3	2.46E-07	1.108	1.31E-06	7.22E-06	4774
RLTPR	4.83E-04	1.014	1.32E-06	7.29E-06	4775
NNAT	1.90E-06	-1.162	1.33E-06	7.35E-06	4780
GPR68	1.55E-08	1.236	1.35E-06	7.45E-06	4787
BGN	1.58E-10	1.372	1.38E-06	7.58E-06	4798
PHYHD1	7.93E-13	-2.944	1.41E-06	7.71E-06	4807
REPS2	7.83E-05	-1.279	1.46E-06	7.99E-06	4824
NR4A1	5.65E-06	-2.008	1.46E-06	8.00E-06	4825
METTL25	5.99E-10	-1.047	1.48E-06	8.07E-06	4830
PKNOX2	8.42E-07	-1.963	1.52E-06	8.26E-06	4842
SPIN3	7.58E-07	-1.116	1.59E-06	8.64E-06	4857
SECTM1	1.44E-04	1.002	1.63E-06	8.84E-06	4870
SSPN	2.21E-06	-1.671	1.65E-06	8.94E-06	4877
TOP2A	3.89E-07	1.664	1.67E-06	9.01E-06	4880

CHTF18	3.34E-08	1.257	1.67E-06	9.04E-06	4881
SOX17	1.71E-04	-1.341	1.70E-06	9.16E-06	4886
UHRF1	2.32E-08	1.887	1.71E-06	9.24E-06	4892
WDR76	1.53E-08	1.321	1.73E-06	9.31E-06	4898
CD1A	1.39E-03	-1.047	1.84E-06	9.84E-06	4926
SLC7A5	1.55E-06	1.337	1.88E-06	1.00E-05	4935
DUSP1	1.59E-06	-1.083	1.88E-06	1.00E-05	4937
CCDC80	3.43E-05	-1.143	1.91E-06	1.02E-05	4944
SLC38A5	8.30E-06	1.933	2.01E-06	1.07E-05	4956
KRT13	7.01E-05	-2.545	2.10E-06	1.11E-05	4978
ZNF577	2.05E-08	-1.031	2.13E-06	1.13E-05	4984
DYRK1B	1.63E-08	-2.188	2.19E-06	1.16E-05	4996
POC1A	6.16E-08	1.293	2.20E-06	1.16E-05	4998
LPL	1.06E-08	-2.637	2.21E-06	1.17E-05	5001
PDZD4	1.08E-10	-1.560	2.31E-06	1.21E-05	5024
DKK3	3.09E-05	1.173	2.37E-06	1.24E-05	5034
ANGPTL7	1.18E-06	-2.357	2.43E-06	1.27E-05	5051
PDLIM3	4.81E-05	-2.309	2.53E-06	1.32E-05	5077
CRAT	2.79E-08	-2.311	2.58E-06	1.34E-05	5082
ANO2	7.76E-08	-1.372	2.65E-06	1.37E-05	5093
CD8A	5.87E-04	1.299	2.65E-06	1.37E-05	5095
SEMA6C	6.50E-04	-3.088	2.69E-06	1.39E-05	5099
WNT3	1.10E-04	1.174	2.76E-06	1.42E-05	5114
FANCA	1.65E-08	1.217	2.86E-06	1.47E-05	5128
GPAM	7.75E-06	-1.475	2.89E-06	1.48E-05	5138
EPPK1	9.10E-04	1.066	3.06E-06	1.56E-05	5159
ABCC6	3.93E-06	-1.665	3.10E-06	1.58E-05	5166
KIF18B	1.68E-07	1.762	3.11E-06	1.58E-05	5168
CAND2	4.32E-07	-3.918	3.15E-06	1.60E-05	5173
RNF157	1.33E-03	-2.762	3.17E-06	1.62E-05	5176
AAMDC	1.21E-05	-1.224	3.18E-06	1.62E-05	5177
PTCD3	1.77E-05	-1.178	3.27E-06	1.66E-05	5188
KLF9	3.79E-06	-1.167	3.39E-06	1.72E-05	5200
APOBEC3G	1.19E-05	1.256	3.51E-06	1.78E-05	5217
ZNF790	1.42E-11	-1.459	3.55E-06	1.79E-05	5221
CP	1.35E-06	-2.530	3.74E-06	1.88E-05	5257
RBP7	1.23E-10	-2.014	3.84E-06	1.92E-05	5266
TONSL	4.48E-09	1.449	3.97E-06	1.98E-05	5283
SHC2	7.66E-08	-1.525	3.99E-06	1.99E-05	5288
NIPAL4	3.01E-04	1.268	4.06E-06	2.02E-05	5296
LRP8	5.41E-07	1.166	4.33E-06	2.14E-05	5323
HIST1H3C	1.93E-07	1.605	4.35E-06	2.15E-05	5328

CYP3A5	8.91E-05	-1.460	4.40E-06	2.17E-05	5336
TOX	2.86E-05	1.109	4.40E-06	2.18E-05	5337
C16orf86	2.92E-07	-1.338	4.44E-06	2.19E-05	5341
MAN1C1	5.48E-07	-1.328	4.47E-06	2.21E-05	5344
TMEM200C	2.10E-03	-2.121	4.63E-06	2.28E-05	5361
COX7A1	2.71E-07	-3.460	4.66E-06	2.29E-05	5368
ZNF502	1.56E-07	-1.084	4.67E-06	2.29E-05	5371
PID1	1.80E-05	-1.034	4.92E-06	2.40E-05	5394
ZNF91	3.07E-11	-2.220	5.06E-06	2.47E-05	5414
RTN4R	4.70E-05	1.180	5.09E-06	2.48E-05	5416
ANK1	3.38E-02	-3.505	5.12E-06	2.49E-05	5419
CYP27A1	5.52E-09	-2.186	5.18E-06	2.52E-05	5425
ZNF415	1.13E-06	-2.146	5.32E-06	2.58E-05	5435
SPINK5	1.29E-02	-1.812	5.36E-06	2.60E-05	5437
SCML2	3.18E-08	-1.231	5.39E-06	2.61E-05	5442
LONRF3	9.12E-08	-2.187	5.63E-06	2.72E-05	5464
PRKAR2B	9.17E-07	-2.033	5.78E-06	2.79E-05	5474
USHBP1	1.37E-04	-1.285	6.31E-06	3.01E-05	5516
RGMA	7.13E-08	-1.475	6.35E-06	3.03E-05	5524
ADAM22	1.44E-08	-1.341	6.57E-06	3.13E-05	5536
CENPF	1.56E-07	1.542	6.94E-06	3.29E-05	5564
RSPO3	1.35E-07	-2.298	6.96E-06	3.30E-05	5566
LYRM9	6.66E-08	-1.568	7.01E-06	3.32E-05	5570
PHGDH	1.26E-07	-1.412	7.07E-06	3.34E-05	5575
CD207	8.68E-04	-1.533	8.09E-06	3.78E-05	5642
HIST1H2BJ	5.52E-09	1.400	8.34E-06	3.89E-05	5652
HIST1H2BH	7.58E-07	1.399	8.35E-06	3.89E-05	5654
DNER	1.44E-02	1.735	8.87E-06	4.12E-05	5681
ASB9	2.96E-05	-2.110	8.89E-06	4.12E-05	5685
C1QTNF9B-AS1	2.74E-05	-1.463	8.89E-06	4.12E-05	5686
ZNF254	3.86E-11	-1.791	9.59E-06	4.41E-05	5725
AKAP12	1.10E-09	-1.688	9.91E-06	4.55E-05	5743
PTTG1	3.00E-06	1.271	1.05E-05	4.78E-05	5778
PTPRO	3.39E-04	1.025	1.06E-05	4.84E-05	5787
TBX15	3.94E-04	-2.905	1.10E-05	4.99E-05	5803
HIST1H4I	1.52E-06	1.574	1.12E-05	5.06E-05	5814
LNX1	3.29E-08	-1.188	1.16E-05	5.23E-05	5838
SGCD	1.48E-04	-1.400	1.21E-05	5.46E-05	5857
SULF2	8.96E-07	1.589	1.30E-05	5.81E-05	5893
GNA14	6.52E-07	-1.299	1.30E-05	5.83E-05	5897
PTGER3	3.68E-07	-1.543	1.36E-05	6.07E-05	5920

PDK4	2.05E-08	-3.124	1.37E-05	6.09E-05	5925
MAP2K6	3.18E-07	-1.566	1.44E-05	6.38E-05	5954
ADORA3	2.73E-07	1.161	1.47E-05	6.51E-05	5968
ZNF493	1.37E-09	-1.311	1.53E-05	6.75E-05	5985
IL17RD	4.78E-10	-1.726	1.55E-05	6.80E-05	5991
OIP5	1.06E-07	1.431	1.55E-05	6.83E-05	5995
GNE	2.22E-04	-1.279	1.55E-05	6.83E-05	5996
MAB21L1	1.63E-08	-2.125	1.57E-05	6.90E-05	6008
SAMD9	1.25E-08	1.360	1.63E-05	7.11E-05	6023
TYMS	7.12E-07	1.416	1.69E-05	7.37E-05	6045
ITGA11	1.38E-04	1.269	1.71E-05	7.47E-05	6049
OGN	3.30E-10	-2.429	1.72E-05	7.48E-05	6050
ARHGAP11A	7.86E-08	1.406	1.76E-05	7.67E-05	6062
ITGA7	6.78E-06	-2.305	1.78E-05	7.72E-05	6068
CCL24	1.12E-03	2.003	1.89E-05	8.16E-05	6105
TRPS1	1.03E-05	-1.015	1.92E-05	8.27E-05	6108
ETV4	1.48E-04	1.150	1.94E-05	8.36E-05	6117
EMCN	9.77E-07	-1.171	1.95E-05	8.40E-05	6121
AKAP7	2.65E-08	-1.277	1.98E-05	8.54E-05	6125
SLC16A7	6.07E-09	-1.327	2.10E-05	8.99E-05	6148
CCDC149	2.47E-05	-1.253	2.10E-05	9.02E-05	6151
SLC25A27	9.91E-09	-1.198	2.11E-05	9.04E-05	6154
FHL1	1.96E-05	-2.790	2.24E-05	9.56E-05	6182
FZD4	1.32E-06	-1.707	2.28E-05	9.72E-05	6193
FAM110B	1.13E-07	-1.989	2.29E-05	9.76E-05	6196
IL31RA	9.76E-06	1.741	2.35E-05	9.97E-05	6208
LRRC4	3.21E-04	-1.298	2.36E-05	1.00E-04	6210
ASPM	1.33E-06	1.439	2.39E-05	1.02E-04	6213
CXCL1	6.50E-09	2.319	2.47E-05	1.05E-04	6235
CD34	1.49E-07	-1.192	2.50E-05	1.05E-04	6238
ZBTB47	3.12E-06	-1.478	2.50E-05	1.06E-04	6239
SORCS2	1.03E-08	1.900	2.71E-05	1.13E-04	6289
TMEM86A	7.12E-05	1.154	2.73E-05	1.14E-04	6293
CEACAM5	1.13E-02	-1.129	2.79E-05	1.17E-04	6304
CLCA2	1.81E-03	1.281	2.97E-05	1.24E-04	6335
COL21A1	2.12E-09	-2.060	3.19E-05	1.32E-04	6370
IVD	2.77E-06	-1.366	3.20E-05	1.32E-04	6373
CPD	1.75E-03	-1.320	3.23E-05	1.33E-04	6378
CXCL12	3.94E-08	-1.543	3.49E-05	1.43E-04	6425
GPRC5C	3.56E-04	-1.158	3.62E-05	1.48E-04	6459
PDE2A	1.32E-06	-1.300	3.67E-05	1.50E-04	6470
LANCL3	6.18E-07	-1.428	3.68E-05	1.50E-04	6471

SMARCD3	1.98E-07	-2.152	3.73E-05	1.52E-04	6481
HIST1H2AB	4.34E-09	1.547	4.14E-05	1.67E-04	6543
HAP1	5.68E-04	2.023	4.23E-05	1.70E-04	6555
COL14A1	6.68E-08	-1.296	4.25E-05	1.71E-04	6556
GPR143	3.07E-04	-1.443	4.26E-05	1.71E-04	6560
COL5A3	8.30E-07	1.783	4.51E-05	1.81E-04	6591
CYP2R1	2.49E-09	-1.171	4.58E-05	1.83E-04	6600
PPP1R3F	3.77E-06	-1.439	4.69E-05	1.87E-04	6614
ALG1L	1.45E-06	1.986	4.74E-05	1.89E-04	6625
CEACAM19	5.42E-04	1.220	5.07E-05	2.01E-04	6655
C15orf62	2.01E-07	-1.107	5.44E-05	2.14E-04	6706
GPR82	6.62E-04	1.025	5.60E-05	2.20E-04	6725
DENND2A	2.91E-06	-1.096	5.62E-05	2.20E-04	6728
PDE1A	1.59E-07	-1.788	5.82E-05	2.27E-04	6747
TOR4A	2.04E-07	1.326	6.07E-05	2.36E-04	6771
NCAM2	8.96E-08	-1.590	6.20E-05	2.41E-04	6784
HIST1H2BL	1.46E-06	1.458	6.21E-05	2.41E-04	6786
LRRC6	7.63E-09	-1.611	6.52E-05	2.52E-04	6811
NUSAP1	1.45E-09	1.426	6.55E-05	2.53E-04	6817
SLC19A3	2.26E-05	-1.427	6.61E-05	2.56E-04	6821
EFHD1	2.57E-06	-1.777	7.11E-05	2.73E-04	6869
KL	3.48E-06	-1.038	7.27E-05	2.78E-04	6884
TMEM98	4.07E-05	-1.013	7.46E-05	2.85E-04	6907
ERVMER34-1	6.07E-05	1.227	7.75E-05	2.95E-04	6937
EBF1	7.37E-07	-1.143	8.06E-05	3.05E-04	6964
HOPX	6.57E-04	-1.243	8.10E-05	3.06E-04	6967
TET1	8.35E-07	-1.948	8.43E-05	3.18E-04	6997
OCLN	2.45E-05	-1.372	9.37E-05	3.50E-04	7058
SGOL1	4.67E-06	1.101	1.11E-04	4.09E-04	7186
NLGN3	3.02E-08	-1.201	1.19E-04	4.35E-04	7230
ZC3H12B	4.62E-08	-1.305	1.21E-04	4.40E-04	7238
SEC14L2	1.24E-05	1.146	1.24E-04	4.50E-04	7256
RGAG4	9.39E-08	-1.276	1.25E-04	4.54E-04	7265
ZSCAN16	2.38E-08	-1.119	1.32E-04	4.76E-04	7293
FMO5	5.10E-05	-2.525	1.34E-04	4.85E-04	7307
CRYAB	2.23E-07	-2.202	1.38E-04	4.96E-04	7324
GATS	5.03E-05	-1.704	1.44E-04	5.15E-04	7355
RTN2	1.10E-02	-1.848	1.51E-04	5.40E-04	7383
ZNF681	5.31E-09	-2.275	1.52E-04	5.41E-04	7384
LYPD5	9.59E-04	1.350	1.57E-04	5.59E-04	7412
MTUS1	4.96E-07	-1.296	1.66E-04	5.88E-04	7448
NEK2	1.05E-06	1.646	1.69E-04	5.97E-04	7459

S100A12	9.49E-03	1.300	1.71E-04	6.05E-04	7469
IGFBP3	2.19E-04	1.380	1.73E-04	6.10E-04	7481
ARMCX2	7.18E-06	-1.660	1.78E-04	6.26E-04	7500
LMTK3	4.48E-03	1.037	1.81E-04	6.34E-04	7513
ACYP2	6.77E-05	-1.189	1.90E-04	6.62E-04	7553
DPF3	2.52E-02	-2.134	2.08E-04	7.18E-04	7619
CD36	6.37E-05	-1.382	2.12E-04	7.30E-04	7636
SGK223	8.63E-06	1.121	2.41E-04	8.23E-04	7722
TMEM143	1.06E-04	-2.001	2.55E-04	8.64E-04	7771
BMPER	4.25E-05	-1.574	2.66E-04	8.97E-04	7816
XDH	3.26E-06	1.824	2.67E-04	9.00E-04	7818
C16orf45	2.47E-06	-1.478	2.67E-04	9.01E-04	7820
MTAP	3.39E-05	1.251	3.05E-04	1.01E-03	7932
TMEM170B	2.70E-04	-1.379	3.19E-04	1.05E-03	7979
CDK20	3.16E-08	-1.091	3.20E-04	1.06E-03	7984
NUPR1	1.13E-07	-1.215	3.34E-04	1.10E-03	8011
LRIG1	2.02E-06	-1.415	3.35E-04	1.10E-03	8017
SATB1	1.33E-05	-1.124	3.56E-04	1.16E-03	8059
RNASE7	1.52E-04	1.543	3.67E-04	1.20E-03	8079
ADAM32	4.56E-07	-1.554	3.70E-04	1.21E-03	8091
ULBP3	9.44E-06	1.630	3.76E-04	1.22E-03	8105
EBF2	3.40E-05	-1.444	4.20E-04	1.35E-03	8185
UBXN10	4.39E-06	-2.108	4.21E-04	1.35E-03	8187
CCSER1	4.77E-07	-2.034	4.45E-04	1.42E-03	8237
BRIP1	1.32E-06	1.371	4.47E-04	1.43E-03	8243
SNTB1	5.56E-04	-1.171	4.54E-04	1.45E-03	8250
PDGFD	5.18E-08	-1.770	4.72E-04	1.50E-03	8285
SEPP1	1.94E-07	-1.091	4.98E-04	1.58E-03	8330
SLCO5A1	1.01E-03	-2.196	5.27E-04	1.66E-03	8382
THBS4	1.36E-06	-1.490	5.66E-04	1.77E-03	8440
ADAMTSL1	4.62E-06	-1.129	5.75E-04	1.79E-03	8455
GJA1	1.02E-04	1.066	5.77E-04	1.80E-03	8463
FOS	5.56E-04	-1.165	5.88E-04	1.83E-03	8482
C2orf88	3.37E-09	-2.042	6.76E-04	2.07E-03	8602
KIT	3.95E-06	-1.016	6.91E-04	2.11E-03	8619
FAM13C	1.84E-07	-1.554	7.23E-04	2.20E-03	8664
ME3	2.99E-07	-1.780	7.28E-04	2.21E-03	8669
GINS4	1.02E-08	1.452	7.33E-04	2.23E-03	8677
SNCA	8.23E-04	-1.085	7.40E-04	2.25E-03	8682
C4orf19	7.48E-04	-3.026	7.43E-04	2.25E-03	8685
TMEM178B	1.08E-06	-2.735	7.45E-04	2.26E-03	8689
KRT6B	1.43E-03	1.097	8.10E-04	2.43E-03	8767

LAMA2	2.13E-05	-1.634	8.68E-04	2.59E-03	8834
MYCN	8.23E-04	-2.365	8.79E-04	2.62E-03	8842
PRSS53	2.14E-05	1.489	9.02E-04	2.68E-03	8869
GPT2	1.36E-08	-1.512	9.27E-04	2.75E-03	8894
P2RY6	2.00E-09	1.816	1.03E-03	3.01E-03	8987
AQP1	7.23E-07	-1.103	1.05E-03	3.06E-03	9010
PARM1	2.67E-05	-1.884	1.07E-03	3.13E-03	9035
CHRNA10	4.35E-04	-3.179	1.09E-03	3.18E-03	9052
SLC27A5	7.71E-09	-1.157	1.13E-03	3.28E-03	9075
POPDC2	3.37E-03	-3.093	1.14E-03	3.32E-03	9095
FBLN5	5.10E-06	-1.165	1.16E-03	3.37E-03	9112
FBXO2	1.54E-03	1.121	1.25E-03	3.60E-03	9170
TESC	5.80E-04	-2.798	1.30E-03	3.72E-03	9213
EGR1	2.89E-05	-1.536	1.32E-03	3.77E-03	9229
GCNT2	7.49E-08	-1.720	1.46E-03	4.12E-03	9352
DNALI1	4.88E-06	-1.700	1.47E-03	4.14E-03	9361
HEPHL1	6.78E-07	1.751	1.48E-03	4.17E-03	9368
SIM2	1.34E-06	-2.798	1.51E-03	4.25E-03	9381
MIPOL1	3.38E-06	-1.253	1.52E-03	4.28E-03	9390
SLC2A10	1.48E-02	-1.591	1.54E-03	4.31E-03	9400
MROH8	5.25E-06	-1.263	1.54E-03	4.32E-03	9403
ZNF610	2.36E-06	-1.535	1.56E-03	4.36E-03	9413
PREX2	9.85E-08	-1.233	1.57E-03	4.40E-03	9417
LCA5L	1.67E-04	-1.317	1.61E-03	4.49E-03	9442
ADSSL1	8.09E-06	-3.392	1.64E-03	4.57E-03	9468
OVGP1	6.15E-08	-1.889	1.74E-03	4.82E-03	9528
FBXL8	9.56E-07	-1.061	1.77E-03	4.88E-03	9546
FGF13	4.66E-07	-2.441	1.81E-03	4.99E-03	9566
AFF3	2.42E-06	-1.820	1.82E-03	5.01E-03	9569
PHKA1	2.42E-05	-1.569	1.82E-03	5.02E-03	9570
CDT1	5.86E-07	1.416	1.89E-03	5.20E-03	9613
C8orf58	7.08E-06	-1.071	1.91E-03	5.23E-03	9619
CMYA5	2.69E-04	-3.215	1.98E-03	5.40E-03	9664
SERINC4	8.21E-05	-1.010	2.06E-03	5.60E-03	9702
C3orf18	3.65E-05	-2.163	2.07E-03	5.62E-03	9704
ESCO2	3.27E-06	1.406	2.32E-03	6.24E-03	9815
ZMAT1	6.13E-08	-1.751	2.61E-03	6.92E-03	9950
FAM64A	8.27E-06	1.453	2.86E-03	7.51E-03	10050
CADM1	3.54E-05	-1.323	2.90E-03	7.60E-03	10064
ZBTB20	1.01E-05	-1.155	2.94E-03	7.68E-03	10077
FNDC1	5.14E-05	2.008	3.00E-03	7.84E-03	10103
ICAM5	2.45E-05	1.628	3.01E-03	7.85E-03	10105

PDE9A	4.84E-05	-1.550	3.14E-03	8.15E-03	10152
IGDCC4	4.06E-04	-1.890	3.56E-03	9.12E-03	10309
ZNF583	5.05E-07	-1.235	3.57E-03	9.12E-03	10316
KIF11	1.99E-06	1.264	3.79E-03	9.62E-03	10392
WDR78	4.14E-08	-1.610	3.86E-03	9.76E-03	10413
PI3	3.30E-04	1.124	3.95E-03	9.97E-03	10449
FAM13A	8.82E-06	-1.096	4.03E-03	1.02E-02	10466
SLC25A25	2.36E-06	-1.178	4.33E-03	1.08E-02	10544
SPC24	4.80E-08	1.654	4.54E-03	1.13E-02	10599
CMA1	5.58E-07	-1.370	4.60E-03	1.14E-02	10614
SPTB	1.18E-02	-1.537	4.62E-03	1.15E-02	10618
MMRN1	1.45E-08	-1.878	5.21E-03	1.28E-02	10778
C8orf4	8.08E-03	-1.357	5.25E-03	1.28E-02	10783
SVIL	2.21E-05	-1.663	5.55E-03	1.35E-02	10843
ATF3	9.00E-04	-1.200	5.88E-03	1.42E-02	10904
KIF5C	2.20E-05	-1.331	6.61E-03	1.58E-02	11037
NTRK2	7.23E-07	-1.624	7.02E-03	1.67E-02	11104
ALDH1A1	3.29E-08	-3.081	7.07E-03	1.68E-02	11116
C14orf159	3.16E-06	-1.393	7.13E-03	1.69E-02	11129
LIMCH1	1.15E-05	-1.659	7.26E-03	1.71E-02	11158
TENM1	1.47E-07	-1.151	7.50E-03	1.77E-02	11192
BSPRY	4.01E-03	-1.109	7.68E-03	1.80E-02	11224
PLA2G16	2.03E-04	-1.556	7.99E-03	1.87E-02	11277
GLIS1	9.08E-04	1.744	8.00E-03	1.87E-02	11282
HPGD	4.69E-09	-1.349	8.17E-03	1.90E-02	11317
ENPP4	1.51E-05	-2.163	8.25E-03	1.92E-02	11333
ZDHHC2	7.60E-05	-1.493	8.81E-03	2.03E-02	11425
NPHP1	3.14E-02	-1.308	8.85E-03	2.04E-02	11430
FAM228B	9.79E-08	-1.765	8.99E-03	2.07E-02	11448
ZNF385D	6.21E-07	-1.309	9.44E-03	2.16E-02	11517
SFRP1	8.08E-05	-1.189	1.02E-02	2.31E-02	11618
CLCN4	3.54E-06	-2.891	1.06E-02	2.39E-02	11666
WIPF3	7.19E-05	-2.188	1.13E-02	2.54E-02	11771
ECHDC3	2.38E-07	-1.786	1.15E-02	2.57E-02	11788
DGCR6	1.85E-04	-2.396	1.19E-02	2.65E-02	11830
MEF2C	1.61E-02	-1.264	1.25E-02	2.77E-02	11905
OBSCN	2.23E-04	-3.678	1.27E-02	2.81E-02	11927
RBMS3	6.19E-05	-1.038	1.29E-02	2.84E-02	11943
NEB	1.11E-03	-3.233	1.31E-02	2.89E-02	11965
FRZB	1.04E-05	-2.432	1.32E-02	2.90E-02	11973
BMX	3.70E-06	-1.296	1.43E-02	3.13E-02	12058
CACNA2D1	9.99E-08	-2.539	1.44E-02	3.15E-02	12069

SLC43A1	1.37E-04	-1.692	1.52E-02	3.31E-02	12130
MPDZ	6.49E-05	-1.203	1.60E-02	3.46E-02	12205
PGM1	2.60E-04	-1.558	1.77E-02	3.78E-02	12343
PFKFB1	4.76E-06	-3.557	1.77E-02	3.78E-02	12344
SVEP1	3.15E-06	-1.182	1.80E-02	3.84E-02	12369
MSS51	3.38E-03	-2.663	1.98E-02	4.17E-02	12508
CDCA7	2.12E-05	1.212	1.99E-02	4.18E-02	12519
HIST1H2AI	1.59E-06	1.343	2.06E-02	4.32E-02	12562
DMD	2.94E-04	-1.860	2.10E-02	4.41E-02	12595
PRPH2	1.48E-05	-2.104	2.26E-02	4.70E-02	12705
PRUNE2	1.93E-04	-2.285	2.36E-02	4.87E-02	12768

Supplemental Table S6: Genes potentially associated with progression of normal tissue to tumor in OSCC-GB

Gene symbol	log2(fold-change)		Results from three-group comparison (TCC)		
	Leukoplakia vs. Normal	Tumor vs. Leukoplakia	p-value	FDR (q-value)	Rank
MMP11	1.020216	3.044026	9.96E-51	1.31E-46	2
PLAU	1.492088	1.956864	1.61E-44	1.41E-40	3
MFAP2	1.148249	2.139568	1.22E-42	6.46E-39	5
LAMC2	1.358986	2.712633	5.36E-40	1.77E-36	8
C1QTNF6	1.273701	2.038755	3.65E-39	1.07E-35	9
ROR1	-1.13635	-1.91844	4.50E-36	9.13E-33	13
TLE2	-1.27637	-1.73529	2.14E-35	3.33E-32	17
COL4A1	1.311001	1.817753	3.32E-35	4.60E-32	19
SERPINE1	2.113255	2.547044	5.52E-35	7.28E-32	20
PCDH17	1.313873	1.660199	4.98E-33	5.47E-30	24
TNFRSF12A	1.919537	1.373264	1.41E-32	1.43E-29	25
MAOB	-1.97398	-2.06088	2.85E-32	2.59E-29	29
MLPH	-2.63087	-1.24517	9.14E-32	8.04E-29	30
STC2	2.030967	1.821287	1.01E-31	8.29E-29	32
ID4	-1.32665	-1.60926	1.86E-31	1.40E-28	35
WISP1	1.245438	1.718123	1.04E-30	6.68E-28	41
MAMDC2	-1.79956	-2.0751	8.98E-30	5.15E-27	46
DDX60L	1.105062	1.444429	1.96E-29	9.61E-27	53
PDPN	1.121459	1.860763	5.74E-29	2.44E-26	62
SYT15	-1.2184	-1.50138	1.23E-28	4.64E-26	70
ADAM12	1.41965	1.480162	3.83E-28	1.28E-25	79
COL4A6	1.793996	1.444543	5.68E-28	1.83E-25	82
FAP	1.066883	1.138368	1.54E-27	4.67E-25	87
C6orf141	1.499558	1.076121	6.13E-27	1.76E-24	92
PLA2G7	3.011632	1.394949	1.14E-26	3.01E-24	100
CYP27B1	1.782263	2.327256	4.25E-26	1.00E-23	112
CTTNBP2	-1.9219	-1.11003	2.81E-25	5.96E-23	124
AIM2	2.653616	1.33279	3.30E-25	6.91E-23	126
HTR7	2.624898	1.026586	3.94E-25	8.18E-23	127
RBM20	-2.44693	-1.17481	6.47E-25	1.27E-22	134
DFNA5	1.255451	1.298117	1.86E-24	3.47E-22	141
GPR39	1.511163	1.561995	1.46E-23	2.28E-21	169
GULP1	-1.54553	-1.35863	2.96E-23	4.36E-21	179
DDX60	1.466277	1.165074	6.98E-23	9.63E-21	191
FCGR3A	2.238692	1.616327	7.07E-23	9.66E-21	193
ZNF471	-1.14891	-1.3337	1.09E-22	1.44E-20	200

OAS2	1.514064	1.074924	1.10E-22	1.44E-20	201
PTHLH	1.95829	1.846266	1.80E-22	2.30E-20	207
PBX1	-1.07633	-1.38112	6.14E-22	7.00E-20	231
STX1A	1.049654	1.006088	6.26E-22	7.11E-20	232
FEZ1	1.052462	1.262162	6.50E-22	7.36E-20	233
TMC7	1.593191	1.113555	1.22E-21	1.31E-19	246
CCRL2	1.272044	1.183083	1.90E-21	1.98E-19	254
SLC2A9	1.064155	1.108806	2.17E-21	2.22E-19	257
SPRY4	1.37231	1.242704	8.01E-21	7.44E-19	284
BATF2	3.584407	1.287817	1.29E-20	1.14E-18	300
PPARGC1A	-1.88749	-1.60338	1.37E-20	1.20E-18	301
RAG1	1.210665	1.8836	1.61E-20	1.40E-18	304
GBP1	1.938725	1.08538	1.79E-20	1.54E-18	307
PLEKHG6	-1.63234	-1.55571	2.58E-20	2.15E-18	317
COL4A2	1.059865	1.436562	3.00E-20	2.46E-18	321
ISG15	3.050087	1.101574	5.29E-20	4.16E-18	335
HERC5	1.656524	1.120313	7.29E-20	5.51E-18	349
GPX3	-1.39979	-1.76356	8.16E-20	6.04E-18	356
CPAMD8	-1.78747	-1.35687	1.74E-19	1.23E-17	374
ANKRD6	-1.43382	-1.10851	1.78E-19	1.25E-17	376
SH3BGRL2	-1.96072	-1.59941	4.56E-19	3.01E-17	399
RNF180	-1.01989	-1.03586	4.67E-19	3.07E-17	401
CDH3	1.819924	1.038488	4.69E-19	3.07E-17	402
TPPP	-1.05333	-1.94662	7.76E-19	4.98E-17	411
USP18	1.44039	1.221511	1.45E-18	8.86E-17	430
SORBS2	-1.40017	-1.30996	1.46E-18	8.89E-17	432
ARHGEF26	-1.02015	-1.9573	1.82E-18	1.09E-16	440
WARS	1.528625	1.272952	1.86E-18	1.11E-16	441
GATM	-1.15077	-1.46109	2.38E-18	1.40E-16	449
ZNF229	-1.1373	-1.22593	5.74E-18	3.15E-16	480
KIAA1211L	-1.79444	-1.25195	7.00E-18	3.83E-16	482
IL34	-1.31027	-1.51175	7.06E-18	3.85E-16	483
PTN	-2.09714	-1.48704	8.00E-18	4.32E-16	488
IDO1	3.490267	1.093208	9.81E-18	5.22E-16	495
PGF	1.651285	1.024393	1.14E-17	5.99E-16	502
IFIT1	1.878339	1.239607	1.19E-17	6.20E-16	505
RSAD2	3.015707	1.184023	1.47E-17	7.48E-16	517
NR3C2	-1.0097	-1.6928	1.96E-17	9.82E-16	527
MMP12	3.376507	1.654515	2.05E-17	1.02E-15	528
PMEL	-1.44765	-3.40154	4.91E-17	2.27E-15	570
PLLP	-1.09051	-1.43973	1.21E-16	5.17E-15	618
FMO2	-1.43941	-2.72551	1.96E-16	8.09E-15	639

CD274	1.378462	1.365084	2.48E-16	1.01E-14	648
LDOC1	-1.71015	-1.20616	4.45E-16	1.72E-14	681
CD300LF	1.880194	1.136528	1.02E-15	3.64E-14	740
CMPK2	2.117229	1.202512	1.12E-15	3.97E-14	743
NEFL	2.327225	1.109878	1.73E-14	4.90E-13	932
GNLY	1.771648	1.027418	2.05E-14	5.70E-13	951
CD80	1.987893	1.190141	3.45E-14	9.20E-13	989
TDRD6	2.29233	1.027	5.11E-14	1.30E-12	1037
SCNN1D	2.565009	1.175888	1.29E-13	3.06E-12	1116
DLG2	-1.09327	-1.38734	1.57E-13	3.64E-12	1137
PNPLA7	-1.03976	-1.29655	1.96E-13	4.48E-12	1155
TREM2	1.530065	1.273309	2.74E-13	6.05E-12	1191
METTL24	-1.04939	-1.71764	7.22E-13	1.48E-11	1285
CXCL10	2.934973	1.129482	4.27E-12	7.41E-11	1520
TLR2	1.085519	1.082118	4.40E-12	7.61E-11	1524
SLC16A6	-1.08776	-1.21117	7.81E-12	1.29E-10	1596
MARC1	-1.17975	-1.24544	1.02E-11	1.63E-10	1638
NBEA	-1.37032	-1.01289	2.21E-11	3.30E-10	1767
PRLR	-1.62191	-1.1419	5.17E-11	7.10E-10	1922
ALDH3A1	-1.63556	-1.17365	6.11E-11	8.27E-10	1947
COBL	-1.04106	-1.15423	2.05E-09	2.05E-08	2649
TMPRSS11A	-1.79703	-1.07716	2.72E-09	2.64E-08	2716
SPAG16	-1.30855	-1.00785	5.77E-09	5.23E-08	2910
CES1	-2.36804	-1.06102	5.52E-07	3.32E-06	4381
RGS11	-1.03156	-1.05609	8.95E-07	5.12E-06	4607
KRT13	-1.24068	-1.51772	2.10E-06	1.11E-05	4978

Supplemental Table S7: Comparison of 8 types of immune cells included in LM22 signature found significantly differentially infiltrated in tumor compared to normal tissue microenvironments in leukoplakia vs. normal (LvN), and tumor vs. leukoplakia (TvL) tissue microenvironments

LM22 immune cells	Tumor vs. Normal (n=72)[1]				Leukoplakia vs. Normal (n=25)[1]		Tumor vs. Leukoplakia (n=25)[1]	
	t-statistic	p-value	Corrected p-value	log2FC	t-statistic	p-value[2]	t-statistic	p-value[2]
B cells naive	2.591	0.015	0.044	-0.122	0.783	0.447	-1.035	0.321
Plasma cells	2.983	0.005	0.017	-1.800	2.978	0.008	0.336	0.742
T cells CD4 memory activated	-4.221	0.002	0.009	2.916	-1.581	0.212	0.687	0.509
NK cells activated	-3.779	0.002	0.010	1.978	-0.767	0.523	1.669	0.237
Monocytes	4.186	0.000	0.001	-1.650	0.272	0.789	-0.414	0.685
Macrophages M1	-7.153	0.000	0.000	1.728	-2.030	0.058	-2.783	0.011
Mast cells activated	-2.265	0.030	0.080	1.690	-1.547	0.262	-3.077	0.012
Neutrophils	3.513	0.002	0.009	-1.072	1.111	0.382	-1.160	0.298

[1]All comparisons were done using paired sample t-tests.
[2]Significant p-values from the LvN & TvL comparisons are marked in red.
