# Supplementary material for: Genes and pathways monotonically dysregulated during progression from normal through leukoplakia to gingivo-buccal oral cancer
Source: NPJ Genom Med. 2021 May 12;6:32. doi: 10.1038/s41525-021-00195-8 (PMC8115176; doi:10.1038/s41525-021-00195-8)
Supplement: Supplementary file 2 — Reporting Summary [file 41525_2021_195_MOESM2_ESM.pdf]

nature research

Corresponding author(s): Partha P. Majumder, Debodipta Das

Last updated by author(s): Mar 7, 2021

Reporting Summary

Nature Research wishes to improve the reproducibility of the work that we publish. This form provides structure for consistency and transparency in reporting. For further information on Nature Research policies, see our Editorial Policies and the Editorial Policy Checklist.

Statistics

For all statistical analyses, confirm that the following items are present in the figure legend, table legend, main text, or Methods section.

n/a	Confirmed	
☐	☒	The exact sample size (n) for each experimental group/condition, given as a discrete number and unit of measurement
☒	☐	A statement on whether measurements were taken from distinct samples or whether the same sample was measured repeatedly
☐	☒	The statistical test(s) used AND whether they are one- or two-sided<br>*Only common tests should be described solely by name; describe more complex techniques in the Methods section.*
☒	☐	A description of all covariates tested
☐	☒	A description of any assumptions or corrections, such as tests of normality and adjustment for multiple comparisons
☐	☒	A full description of the statistical parameters including central tendency (e.g. means) or other basic estimates (e.g. regression coefficient) AND variation (e.g. standard deviation) or associated estimates of uncertainty (e.g. confidence intervals)
☐	☒	For null hypothesis testing, the test statistic (e.g. F, t, r) with confidence intervals, effect sizes, degrees of freedom and P value noted<br>*Give P values as exact values whenever suitable.*
☒	☐	For Bayesian analysis, information on the choice of priors and Markov chain Monte Carlo settings
☒	☐	For hierarchical and complex designs, identification of the appropriate level for tests and full reporting of outcomes
☒	☐	Estimates of effect sizes (e.g. Cohen's d, Pearson's r), indicating how they were calculated

Our web collection on statistics for biologists contains articles on many of the points above.

Software and code

Policy information about availability of computer code

Data collection	NOT APPLICABLE
Data analysis	Standard open source packages, R and Bioconductor have been used. These are clearly mentioned in the manuscript.

For manuscripts utilizing custom algorithms or software that are central to the research but not yet described in published literature, software must be made available to editors and reviewers. We strongly encourage code deposition in a community repository (e.g. GitHub). See the Nature Research guidelines for submitting code & software for further information.

Data

Policy information about availability of data

All manuscripts must include a data availability statement. This statement should provide the following information, where applicable:

- Accession codes, unique identifiers, or web links for publicly available datasets
- A list of figures that have associated raw data
- A description of any restrictions on data availability

Provide your data availability statement here.

Field-specific reporting

Please select the one below that is the best fit for your research. If you are not sure, read the appropriate sections before making your selection.

☒ Life sciences ☐ Behavioural & social sciences ☐ Ecological, evolutionary & environmental sciences

For a reference copy of the document with all sections, see nature.com/documents/nr-reporting-summary-flat.pdf

Life sciences study design

All studies must disclose on these points even when the disclosure is negative.

Sample size	Disclosed in manuscript
Data exclusions	Not applicable
Replication	Validation done; mehods have been isclosed in manuscript
Randomization	Not applicable
Blinding	Not applicable

Reporting for specific materials, systems and methods

We require information from authors about some types of materials, experimental systems and methods used in many studies. Here, indicate whether each material, system or method listed is relevant to your study. If you are not sure if a list item applies to your research, read the appropriate section before selecting a response.

Materials & experimental systems

n/a	Involved in the study	
☒	☐	Antibodies
☒	☐	Eukaryotic cell lines
☒	☐	Palaeontology and archaeology
☒	☐	Animals and other organisms
☐	☒	Human research participants
☐	☒	Clinical data
☒	☐	Dual use research of concern

Methods

n/a	Involved in the study	
☒	☐	ChIP-seq
☒	☐	Flow cytometry
☒	☐	MRI-based neuroimaging

Human research participants

Policy information about studies involving human research participants

Population characteristics	Seventy-two patients suffering from OSCC-GB who reported for treatment to ACTREC, RADCH or CNCI were recruited into this study. Patient details have been provided in Table 1 of the manuscript
Recruitment	Seventy-two patients suffering from OSCC-GB who reported for treatment to ACTREC, RADCH or CNCI were recruited into this study. These werer consecutive patients, unless there was refusal to provide informed consent by the patient.
Ethics oversight	This study was approved by the Research Ethics Committees of Advanced Centre for Treatment, Research and Education in Cancer (ACTREC), Dr. R. Ahmed Dental College & Hospital (RADCH), Chittaranjan National Cancer Institute (CNCI), National Institute of Biomedical Genomics (NIBMG), and Indian Statistical Institute (ISI). A signed informed consent was obtained from each participant before their enrolment into this study.

Note that full information on the approval of the study protocol must also be provided in the manuscript.

Clinical data

Policy information about clinical studies

All manuscripts should comply with the ICMJE guidelines for publication of clinical research and a completed CONSORT checklist must be included with all submissions.

Clinical trial registration	n/a
Study protocol	This study protocol was approved by the Research Ethics Committees of Advanced Centre for Treatment, Research and Education in Cancer (ACTREC), Dr. R. Ahmed Dental College & Hospital (RADCH), Chittaranjan National Cancer Institute (CNCI), National Institute

	of Biomedical Genomics (NIBMG), and Indian Statistical Institute (ISI). A mid-course report on adherence to study protocol was provided to the Ethics committee of NIBMG.
Data collection	Hospitals in the city of Kolkata, India
Outcomes	Seventy-two patients suffering from OSCC-GB who reported for treatment to ACTREC, RADCH or CNCI were recruited into this study. When examination carried out by an oncologist revealed the presence of clinical features relevant to this study, the patient was recruited into the study and the clinical and other relevant data were collected.
